# Total Synthesis of (±)-Stemocurtisine via Catalytic Carbonylation

**DOI:** 10.1021/jacs.6c12593

**Published:** 2026-08-25

**Authors:** Mingyu Zhang, Hailey Yu, Xiao Huang, Mingji Dai

**Affiliations:** † Department of Chemistry, 1371 Emory University, Atlanta, Georgia 30322, United States; ‡ Department of Pharmacology and Chemical Biology, School of Medicine, 1371 Emory University, Atlanta, Georgia 30322, United States

## Abstract

We report herein the first total synthesis of (±)-stemocurtisine, a rare insecticidal *Stemona* alkaloid natural product possessing a cage-like polycyclic skeleton with five *O*/*N*-heterocycles including a characteristic pyrido­[1,2-*a*]­azepine ring system and a highly oxidized tetrasubstituted double bond. Our synthesis features a palladium-catalyzed cyclopropanol ring-opening carbonylative lactonization to build a fused bicyclic lactone as a platform to construct the rest of the ring systems and an unusual bis­(iodozincio)­methane-mediated cyclopropanation of an α,β-epoxyketone to prepare the key cyclopropanol substrate. Two complementary approaches were developed to build the pyrido­[1,2-*a*]­azepine ring system. The first one is based on conventional nitrogen alkylation chemistry using S_*N*_2 reactions. The second one features modern transition metal-catalyzed borrowing hydrogen chemistry to form two C–N bonds intramolecularly between a free amine and two primary alcohols under redox neutral conditions, which is more concise and atom-economical. Lastly, the tetrasubstituted double bond was installed using a Horner–Wadsworth–Emmons olefination protocol followed by oxidation state adjustment. Overall, these enabling transformations led to (±)-stemocurtisine in 15 or 17 steps from two known and readily available starting materials.

## Graphical abstract

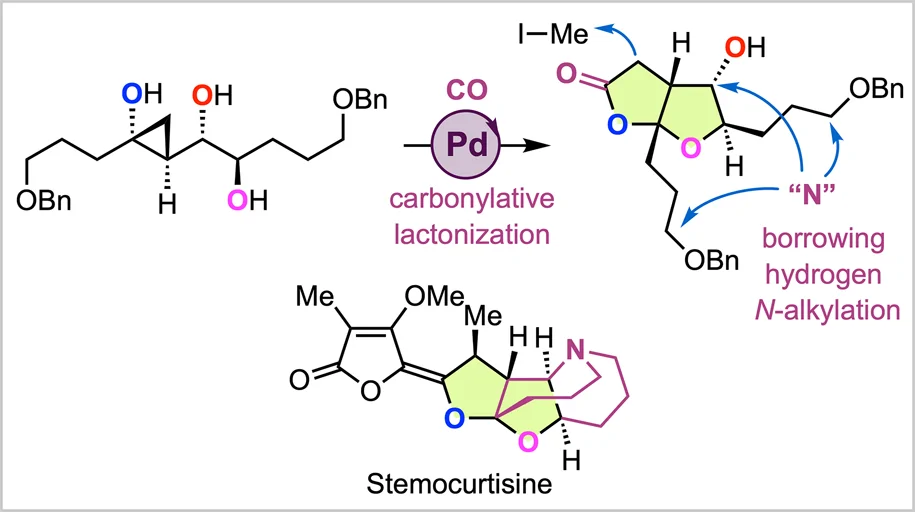

The *Stemona* alkaloids have attracted a significant amount of attention from both the synthetic and medicinal communities due to their diverse, novel, and complex structures and broad-spectrum biological activities.^1^ Within this family, stemocurtisine (**1**, Figure 1A, also named pyridostemin) and a few closely related family members including stemocurtisinol (**2**) and oxystemokerrin (**3**), isolated from a root extract of *Stemona curtisii*, represent unusual architectural outliers.^2^ While many *Stemona* alkaloids are built on a pyrrolo­[1,2-*a*]­azepine platform and have been the focus of the recent total synthesis practices,^3^ stemocurtisine (**1**) and its analogs (**2** and **3**) feature a unique pyrido­[1,2-*a*]­azepine moiety and had not been synthesized before despite several attempts.^4^ Their pyrido­[1,2-*a*]­azepine moiety is embedded in a cage-like polycyclic ring system, which contains a hydrofuro­[2,3-*b*]­furan moiety and is further connected with a γ-butenolide with a highly oxygenated tetrasubstituted double bond. These structural features render them among the most complex *Stemona* alkaloids isolated so far and significantly enhance the difficulty level for their total synthesis. In addition, stemocurtisine (**1**) and stemocurtisinol (**2**) were reported to possess potent insecticidal activity on mosquito larvae with 18 and 39 ppm LC_50_ values, respectively.^2b^ Stemocurtisine (**1**) and oxystemokerrin (**3**) were also discovered to have a synergistic growth inhibitory effect with a chemotherapeutic agent such as vinblastine and can increase the chemosensitivity of KB-V1 cells (multidrug resistance human cervical carcinoma with P-gp expression) to vinblastine at a noncytotoxic concentration.^5^ Overall, their unique and complex architectures in combination with their promising biological activities and isolation burden make stemocurtisine (**1**) and its natural congeners challenging but appealing target molecules for total synthesis.

**Figure 1:**
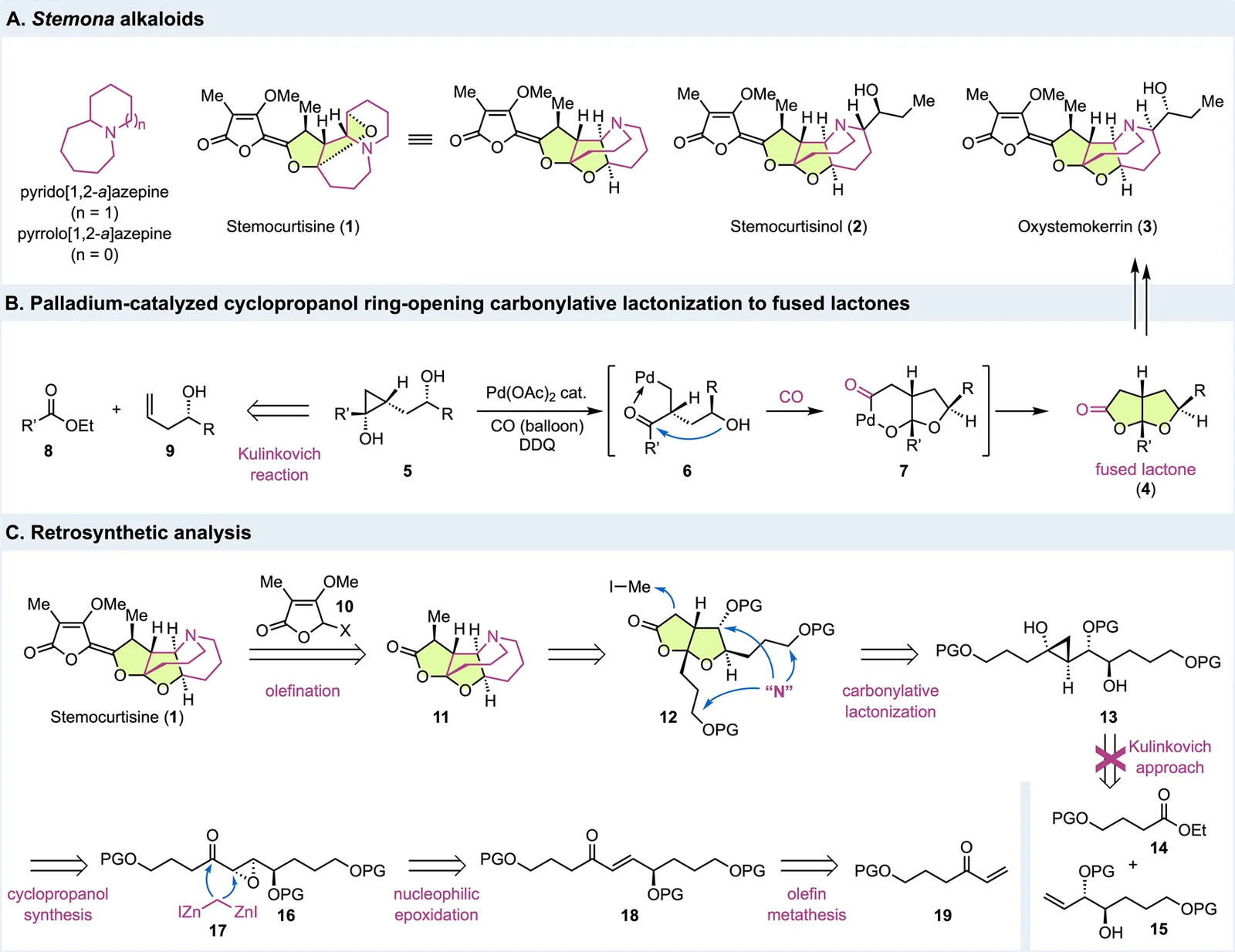
Structures, previous carbonylation methodology, and retrosynthetic analysis.

In continuation of our interest in developing novel catalytic carbonylation strategies and methodologies to facilitate total synthesis,^6^ in 2020, we reported a palladium-catalyzed cyclopropanol ring-opening carbonylative lactonization method to synthesize fused bicyclic lactones, especially tetrahydrofuro­[2,3-*b*]­furanone (cf. **5** → **4**, Figure 1B).^7^ The corresponding cyclopropanol substrate can be prepared from an alkene and an ester via a modified Kulinkovich protocol developed by Cha and co-workers.^8^ Pattern recognition analysis^9^ and our long-term interest in the *Stemona* alkaloid family^10^ led us to stemocurtisine (**1**) and its analogs. The tetrahydrofuro­[2,3-*b*]­furanone product obtained from our cyclopropanol ring-opening carbonylative lactonization can be mapped onto the hydrofuro­[2,3-*b*]­furan structure (highlighted in green) hidden in the polycyclic skeleton of stemocurtisine and could be used as a platform to build the pyrido­[1,2-*a*]­azepine moiety. The carbonyl group of the lactone could be used as a handle to link with the γ-butenolide moiety. Retrosynthetically, inspired by several elegant prior arts of the *Stemona* alkaloid syntheses,^11^ we proposed that stemocurtisine (**1**) could be synthesized from γ-butenolide derivative **10** and advanced intermediate **11** via an olefination protocol (Figure 1C). Intermediate **11** could be derived from bicyclic lactone **12** via a convex face α-methylation and a formal triple nitrogen alkylation. Compound **12** could be generated from cyclopropanol **13** using the palladium-catalyzed carbonylative lactonization method we developed previously. In principle, cyclopropanol **13** could be synthesized in a convergent manner from ester **14** and terminal olefin **15** using a Kulinkovich reaction, but our various early attempts to realize such Kulinkovich cyclopropanol synthesis were unsuccessful.^12^ We learned that neither oxygen nor nitrogen functionalities at the allylic position of **15** can be tolerated. Therefore, an alternative cyclopropanol synthesis would be needed. Cyclopropanol **13** was then traced back to α,β-epoxyketone **16**. We envisioned a cascade process involving a stereoselective 1,2-addition followed by a stereospecific nucleophilic epoxide ring opening using bis­(iodozincio)­methane **17**, a method developed by Matsubara and co-workers but was not used in any total synthesis before, to synthesize cyclopropanol **13**.^13^ α,β-Epoxyketone **16** could be prepared via nucleophilic epoxidation of α,β-unsaturated ketone **18**, which could be synthesized from simple enone **19** using an olefin metathesis strategy.

Our synthesis started with known enone **20** and allylic alcohol **21** (Scheme 1).^14^ Cross metathesis between **20** and **21** with Hoveyda-Grubbs II catalyst gave γ-hydroxy enone **22** in 86% yield. The secondary alcohol was then protected as either a TBDPS ether (**23a**) or MOM ether (**23b**) to explore the protecting group effect on the following nucleophilic epoxidation, which turned out to be nontrivial. The commonly used nucleophilic epoxidation conditions including H_2_O_2_ or Ph_3_COOH with bases either gave a 1:1 mixture of diastereomers or failed to deliver the epoxidation product. Eventually, we identified a method developed by Yamamoto et al. using diethylzinc and oxygen^15^ to afford the desired product in good yield. Interestingly, the protecting group on the secondary alcohol exerted a significant influence on the stereochemical outcome of the epoxidation (see the Supporting Information). High diastereoselectivity (dr > 20:1) was obtained with the MOM protected substrate, but the selectivity dropped to 3.3:1 with the TBDPS protected one. We next focused on the cyclopropanol synthesis using the Matsubara protocol.^13^ While the MOM protecting group helped to give high diastereoselectivity in the epoxidation step, it was not effective for the cyclopropanol synthesis with freshly generated bis­(iodozincio)­methane. Only less than 5% of **25b** was produced from **24b**. On the other hand, treatment of the TBDPS protected substrate **24a** (dr 3.3:1) with bis­(iodozincio)­methane delivered a 3.1:1 mixture of cyclopropanols in 73% yield with **25a** as the major product, indicating the importance of a nonchelating protecting group for the cyclopropanol formation. At this stage, to avoid protecting group manipulations, we decided not to protect the newly generated C10a (numbering of stemocurtisine) secondary alcohol despite the potential competing pathways it could generate in the following carbonylation step. Removal of the TBDPS group gave triol **26** in 85% yield. Due to the existence of the C10a free alcohol, we anticipated two potential pathways for the next cyclopropanol ring-opening carbonylative lactonization. Ideally, we were hoping the C1 alcohol (see **27**) would cyclize with the newly generated ketone from the cyclopropanol ring opening and then the hemiacetal intermediate would cyclize with the acyl palladium to form the desired product **29**. In addition, the C10a alcohol could undergo lactonization with the acyl palladium to lead to byproduct **28** or its ketone form, which may be further converted to **29** via a transesterification process. Indeed, when substrate **26** was subjected to our previously reported carbonylative lactonization conditions using Pd­(OAc)_2_ and DDQ under balloon pressure of carbon monoxide in benzene, **28** and **29** were produced initially as a 1.2:1 mixture slightly favoring the undesired product **28**. Further optimization identified that addition of triethylamine at the end of the carbonylation could partially isomerize **28** to **29** and afford overall 50% of **29** and 30% of **28** at gram scale. The isolated **28** could be further converted to **29** with triethylamine to improve the overall yield of **29**. The subsequent mesylate formation followed by S_*N*_2 substitution using sodium azide as the nucleophile introduced the desired nitrogen functionality at C10a and advanced **29** to **31**. Notably, using a one-step Mitsunobu protocol to convert **29** to **31** was unsuccessful due to the concave steric hindrance experienced by the secondary alcohol. Azide **31** underwent convex face α-methylation to give **32** in high yield.

**Scheme 1:**
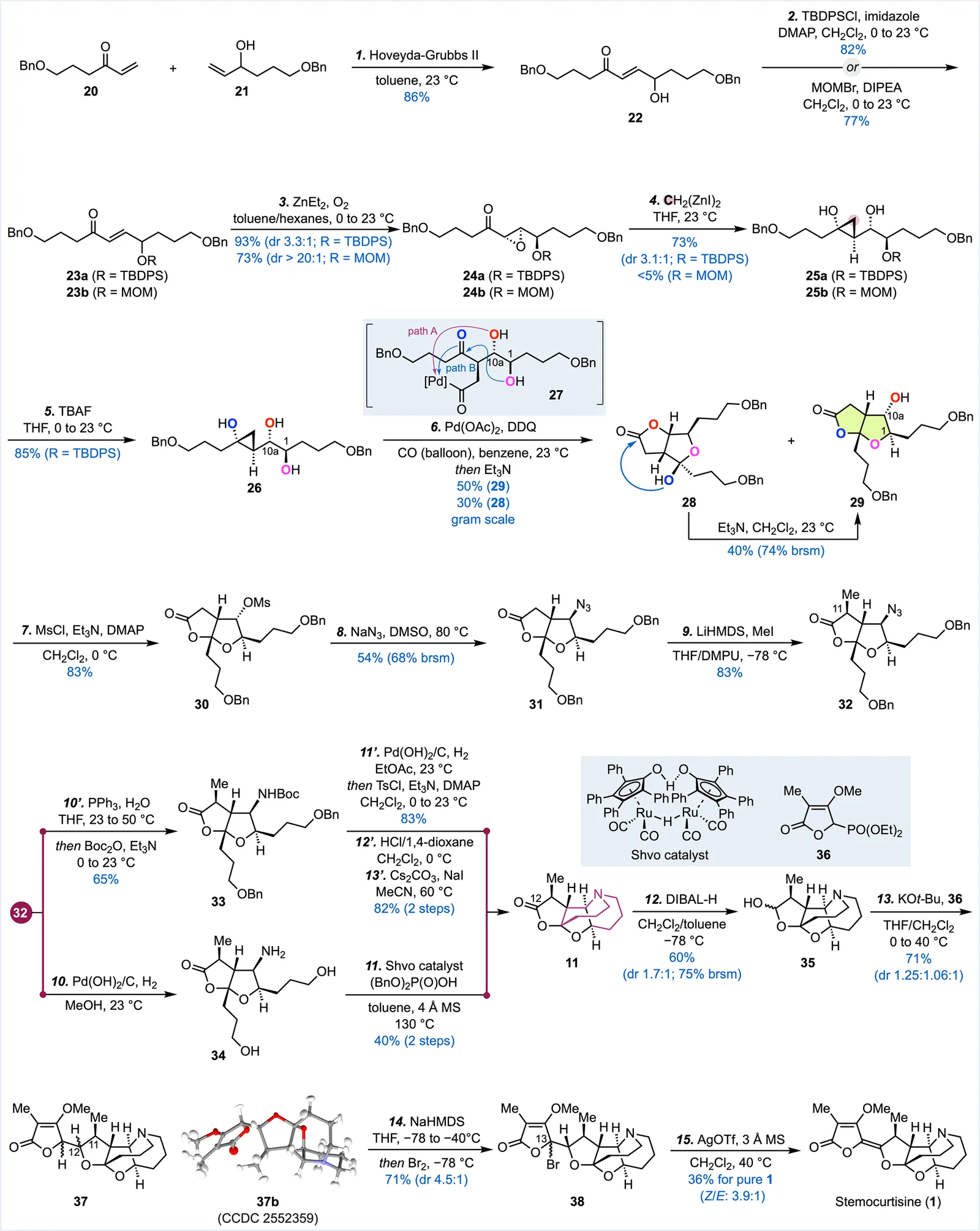
Total Synthesis of (±)-Stemocurtisine

With **32** in hand, we next focused on constructing the pyrido­[1,2-*a*]­azepine moiety by double intramolecular nitrogen alkylation. Two complementary approaches were developed for this purpose. The first one involves conventional S_*N*_2 nitrogen alkylation chemistry. This sequence starts with a one-pot Staudinger reduction of the azide with triphenylphosphine followed by protecting the resulting amine as Boc-carbamate. After removal of the two benzyl protecting groups via hydrogenolysis, the corresponding primary alcohols were activated as tosylates. After the Boc protecting group was removed under acidic conditions, a double nitrogen alkylation occurred by treating the resulting amine with Cs_2_CO_3_ and NaI. In this sequence, compound **11** was produced from **32** in 4 steps in 44% total yield. While the reactions involved here are straightforward and reliable for pushing through materials, this sequence is lengthy and not atom-economical due to nonstrategic steps for protection/deprotection and alcohol activation.^16^ We then investigated the possibility of using the recently developed transition metal-catalyzed borrowing hydrogen reaction^17^ to realize a more efficient and atom-economical process to prepare **11**. In this new approach, **32** was first converted to **34** via a one-pot azide reduction and benzyl group deprotection under the conditions of Pd­(OH)_2_/C and H_2_. This reaction was clean, and the crude product was used in the next step to avoid dealing with the purification problems associated with **34**, a highly polar compound with a basic free amine and two free primary alcohols. We then investigated various borrowing hydrogen reaction conditions to achieve a one-step conversion of **34** to **11**. Inspired by the cooperative catalyst systems developed by Barta, Zhao, and others, we employed a combination of the Shvo’s catalyst and dibenzyl phosphate to successfully deliver **11** in 40% yield in 2 steps.^17^

With tetracyclic product **11** in hand, we next focused on appending the γ-butenolide moiety on C12 by forming the required tetrasubstituted double bond. Such tetrasubstituted double bond formation has been shown to be very challenging in other related *Stemona* alkaloid syntheses such as stemofoline and protostemonine.^11^ After several failed attempts to form this tetrasubstituted double bond using γ-butyrolactone **11** directly, we reduced lactone **11** to lactol **35**. The latter was then treated with phosphonate **36** in the presence of potassium *tert*-butoxide to promote a Horner–Wadsworth–Emmons (HWE) olefination reaction,^11e^ which was followed by a spontaneous 1,6-addition to further convert the olefination product to **37** as a mixture of diastereomers (**37a**:**37b**:**37c** = 1.25:1.06:1). Products **37a** and **37b** have the C12 hydrogen atom *cis* to the C11 methyl group (see the Supporting Information). **37b** can be separated from this mixture and was unambiguously confirmed by X-ray crystallographic analysis (CCDC 2552359). γ-Bromination of the mixture of **37** using NaHMDS and Br_2_ afforded **38** as an inseparable mixture at C13 (dr 4.5:1) in 71% yield. The apparent stereoconvergence may arise from reversible ring opening to a transient C12–C13 unsaturated intermediate followed by substrate-controlled recyclization, consistent with Huang’s observations in their stemofoline synthesis.^11e^ A subsequent silver-promoted elimination^11h^ produced pure (±)-stemocurtisine (**1**) in 36% yield and a small amount of the corresponding double bond isomer (*Z*/*E* 3.9:1; see the Supporting Information). In addition, enantioenriched **21** can be prepared using a literature reported procedure^14c^ or a Ni-catalyzed asymmetric 1,2-reduction of enone **20** (see the Supporting Information).^18^ It can be further advanced to **24a** with high stereochemical fidelity, indicating the feasibility of adapting the current approach for asymmetric total synthesis of stemocurtisine.

In summary, the first total synthesis of (±)-stemocurtisine was completed. Our synthesis features a palladium-catalyzed cyclopropanol ring-opening carbonylative lactonization to build a bicyclic lactone hidden in the tetracyclic skeleton of stemocurtisine. The cyclopropanol substrate was prepared using the Matsubara cyclopropanol synthesis with bis­(iodozincio)­methane as a dicarbanion equivalent to react with an α,β-epoxyketone. The latter was prepared via the Yamamoto nucleophilic epoxidation protocol, which uses a combination of diethylzinc and oxygen. Two approaches were developed to form the characteristic pyrido­[1,2-*a*]­azepine of stemocurtisine. One involves conventional S_*N*_2 nitrogen alkylation chemistry. The other one uses a redox neutral transition metal-catalyzed borrowing hydrogen chemistry to form two C–N bonds in one step from an amine and two alcohols, which is more concise and atom-economical. Toward the end, the tetrasubstituted double bond was formed via a sequence of HWE olefination, bromination, and silver-promoted elimination. Overall, (±)-stemocurtisine was prepared in 15 or 17 steps from known compounds **20** and **21**. This synthesis highlights the synthetic efficiency of our previously developed cyclopropanol ring opening carbonylative lactonization chemistry and the potential of the borrowing hydrogen C–N bond formation chemistry in complex natural product total synthesis.

## Supplementary Material



## Notes

Experimental procedures and NMR spectra for all new compounds (PDF)

## Notes

This paper is dedicated to Professor Huw M. L. Davies on the occasion of his 70th birthday.

## Abbreviations

Ac: acetyl
Bn: benzyl
Boc: *tert*-butoxycarbonyl
DDQ: 2,3-dichloro-5,6-dicyano-1,4-benzoquinone
DIBAL-H: diisobutylaluminum hydride
DIPEA: *N*,*N*-diisopropylethylamine
DMAP: 4-(dimethylamino)­pyridine
DMPU: *N*,*N*′-dimethylpropyleneurea
DMSO: dimethyl sulfoxide
LiHMDS: lithium bis­(trimethylsilyl)­amide
MOM: methoxymethyl
MS: molecular sieve
Ms: methanesulfonyl
NaHMDS: sodium bis­(trimethylsilyl)­amide
PG: protecting group
TBAF: tetra-*n*-butylammonium fluoride
TBDPS: *tert*-butyldiphenylsilyl
Tf: trifluoromethanesulfonyl
THF: tetrahydrofuran
Ts: *p*-toluenesulfonyl
