# Supplementary material for: Total Synthesis of (±)-Stemocurtisine via Catalytic Carbonylation
Source: J Am Chem Soc. 2026 Aug 25;148(35):37600–5. doi: 10.1021/jacs.6c12593 (PMC13564418; doi:10.1021/jacs.6c12593)
Supplement: Supplementary file 1 [file ja6c12593_si_001.pdf]

Supporting Information

Total Synthesis of (±)-Stemocurtisine via Catalytic Carbonylation

Mingyu Zhang[1], Hailey Yu[1], Xiao Huang[1], Mingji Dai[*,1,2]

[1] Department of Chemistry, Emory University, Atlanta, Georgia 30322, United States.

[2] Department of Pharmacology and Chemical Biology, School of Medicine, Emory University, Atlanta, Georgia 30322, United States.

Table of Contents

A. General Methods…………………………………………………………………………S3

B. Experimental Procedures and Characterization Data………………………………….…..S3

C. NMR Comparison Tables…………………………………………………….………...S27

D. X-ray Crystal Data and Experimental…………………………………………….…..S28

E. References………………………………………………………………………….S29

F. ^{1}H, ^{13}C and 2D NMR Spectra……………………………………………………...S30

A. General Methods.

NMR spectra were recorded on Bruker spectrometers (^{1}H at 400, 600 MHz and ^{13}C at 101, 151 MHz). Chemical shifts (δ) were given in ppm with reference to solvent signals [^{1}H NMR: $CDCl_3$ (7.26), MeOD (3.31); ^{13}C NMR: $CDCl_3$ (77.2), MeOD (49.0)]. ^{1}H NMR data are reported as follows: chemical shift (δ ppm), multiplicity (s = singlet, d = doublet, t = triplet, q = quartet, m = multiplet, br = broad, app = apparent), coupling constant (Hz), and integration. IR spectra were collected on a Nicolet iS10 FT-IR spectrometer. Mass spectra were taken on a Thermo Finnigan LTQFTMS spectrometer with APCI, ESI. All reactions were carried out under an inert argon atmosphere with dry solvents under anhydrous conditions unless otherwise stated. Tetrahydrofuran (THF), toluene (PhMe) and dichloromethane (DCM) were dried by passage through activated alumina columns under an argon atmosphere via PureProcessTechnology GS-SPS-5-CM system. All other solvents and reagents were used as obtained from Sigma Aldrich, Fisher Scientific, Oakwood Chemicals, Ambeed, and ChemScene without further purification. Reactions were monitored by thin layer chromatography (TLC), using UV light, anisaldehyde stain, phosphomolybdic acid (PMA) stain and/or Dragendorff's reagent as the visualizing agent. Column chromatography was performed on silica gel. Room temperature (r. t.) is around 23 °C.

B. Experimental Procedures and Characterization Data.

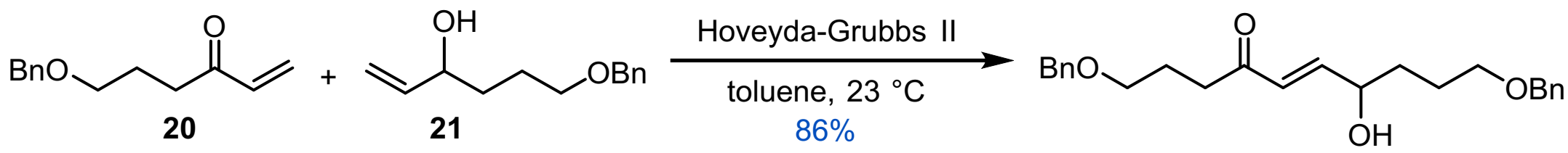

A round-bottom flask was charged with allylic alcohol **21** (1.0 equiv, 5.10 g, 24.72 mmol), enone **20** (2.86 equiv, 14.43 g, 70.64 mmol, synthesized according to literature-reported procedures[1,2]), and toluene (125 mL). The flask was equipped with an argon inlet and a bubbler, and the solution was sparged with argon for 45 min (~1 bubble per second). A solution of Hoveyda–Grubbs second-generation catalyst (0.025 equiv, 392.5 mg, 0.626 mmol) in CH_2Cl_2 (3 mL) was then added dropwise. The reaction mixture was stirred vigorously at room temperature for 2 h. Upon complete consumption of allylic alcohol **21**, the mixture was concentrated directly *in vacuo*. The residue was purified by silica gel chromatography (hexanes/EtOAc = 15:1 → 5:1 → 1:1) to afford *γ*-hydroxyenone **22** as a brown oil (8.12 g, 86%), along with recovered enone **20** (8.73 g).

Compound 22:

^{1}H NMR (400 MHz, $CDCl_3$) δ 7.39 – 7.26 (m, 10H), 6.79 (dd, J = 15.8, 4.6 Hz, 1H), 6.32 (dd, J = 15.8, 1.7

Hz, 1H), 4.53 (s, 2H), 4.49 (s, 2H), 4.36 – 4.27 (m, 1H), 3.52 (t, J = 5.5 Hz, 2H), 3.50 (t, J = 6.1 Hz, 2H), 3.17 (d, J = 4.5 Hz, 1H), 2.67 (t, J = 7.2 Hz, 2H), 1.99 – 1.89 (m, 2H), 1.84 – 1.71 (m, 3H), 1.68 – 1.59 (m, 1H).

^{13}C NMR (101 MHz, CDCl$_3$) δ 200.2, 148.0, 138.6, 137.9, 128.6, 128.5, 128.2, 128.0, 127.9, 127.8, 127.7, 73.4, 73.0, 70.9, 70.4, 69.5, 37.5, 34.5, 26.0, 24.2.

IR (thin film): 3426, 3030, 2929, 2858, 1671, 1632, 1452, 1362, 1100, 1029, 983, 740, 700 cm^{-1}.

HRMS (APCI+): *m/z* Calc. for $C_{24}H_{31}O_4$ $[M+H]^+$: 383.2217, found: 383.2219.

TLC: R_f = 0.36 (hexanes: EtOAc = 1:1, anisaldehyde, UV active).

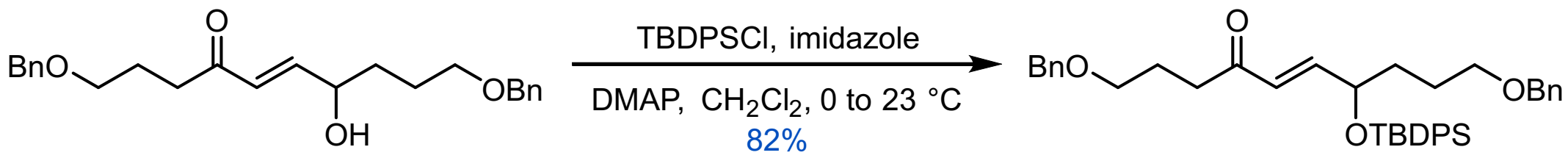

22 **23a**

A round-bottom flask was charged with γ-hydroxyenone **22** (1.0 equiv, 5.16 g, 13.49 mmol), imidazole (2.0 equiv, 1.86 g, 26.98 mmol), and 4-dimethylaminopyridine (0.15 equiv, 253.4 mg, 2.02 mmol). CH_2Cl_2 (27 mL) was added, and the mixture was stirred at room temperature until all solids were dissolved. The solution was cooled to 0 °C, and *tert*-butyldiphenylsilyl chloride (1.5 equiv, 5.3 mL, 20.24 mmol) was added dropwise. After completion of the addition, the reaction mixture was allowed to warm slowly to room temperature and stirred overnight. The reaction was quenched with *sat.* NH_4Cl aq. (54 mL), and water was added to dissolve all precipitates. The aqueous layer was extracted with CH_2Cl_2 (30 mL × 3), and the combined organic layers were dried over sodium sulfate, filtered, and concentrated *in vacuo*. The residue was purified by silica gel chromatography (hexanes/EtOAc = 12:1 → 10:1) to afford enone **23a** as a colorless oil (6.91 g, 82%).

Compound 23a:

^{1}H NMR (400 MHz, CDCl$_3$) δ 7.68 – 7.57 (m, 4H), 7.44 – 7.21 (m, 16H), 6.65 (dd, J = 15.9, 5.6 Hz, 1H), 6.05 (dd, J = 15.9, 1.5 Hz, 1H), 4.49 (s, 2H), 4.41 (s, 2H), 4.40 – 4.34 (m, 1H), 3.47 (t, J = 6.2 Hz, 2H), 3.39 – 3.26 (m, 2H), 2.64 – 2.44 (m, 2H), 1.94 – 1.80 (m, 2H), 1.68 – 1.48 (m, 4H), 1.08 (s, 9H).

^{13}C NMR (101 MHz, CDCl$_3$) δ 200.1, 147.7, 138.58, 138.56, 136.0, 135.9, 133.8, 133.6, 129.95, 129.90, 128.9, 128.49, 128.46, 127.8, 127.73, 127.71, 127.69 (2C), 127.6, 72.94, 72.89, 72.6, 70.2, 69.5, 36.9, 33.7, 27.1, 24.6, 24.1, 19.5.

IR (thin film): 2933, 2859, 1673, 1428, 1362, 1265, 1103, 981, 735, 700 cm^{-1}.

HRMS (APCI+): *m/z* Calc. for $C_{40}H_{49}O_4Si$ $[M+H]^+$: 621.3395, found: 621.3404.

TLC: R_f = 0.54 (hexanes: EtOAc = 4:1, anisaldehyde, UV active).

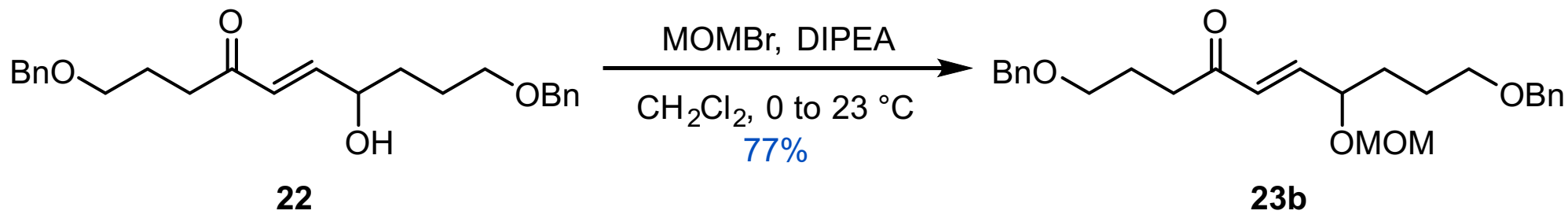

A round-bottom flask was charged with *γ*-hydroxyenone **22** (1.0 equiv, 335.0 mg, 0.876 mmol), *N*,*N*-diisopropylethylamine (10.0 equiv, 1.53 mL, 8.76 mmol), and CH_2Cl_2 (12 mL). The solution was cooled to 0 °C, and bromomethyl methyl ether (5.0 equiv, 90%, 0.40 mL, 4.38 mmol) was added dropwise. After completion of the addition, the reaction mixture was allowed to warm to room temperature and stirred for 8 h. Another portion of bromomethyl methyl ether (2.5 equiv, 90%, 0.20 mL, 2.19 mmol) was then added, and the mixture was stirred for 4 h. The reaction was quenched with *sat.* NH_4Cl aq. (30 mL). The aqueous layer was extracted with CH_2Cl_2 (30 mL × 3), and the combined organic layers were dried over sodium sulfate, filtered, and concentrated *in vacuo*. The residue was purified by silica gel chromatography (hexanes/EtOAc = 8:1 → 5:1) to afford enone **23b** as a pale-yellow oil (287.8 mg, 77%).

Compound 23b:

^{1}H NMR (600 MHz, $CDCl_3$) δ 7.36 – 7.25 (m, 10H), 6.67 (dd, *J* = 16.0, 6.4 Hz, 1H), 6.23 (dd, *J* = 16.0, 1.2 Hz, 1H), 4.62 (d, *J* = 6.9 Hz, 1H), 4.58 (d, *J* = 6.8 Hz, 1H), 4.50 (s, 2H), 4.49 (s, 2H), 4.24 – 4.17 (m, 1H), 3.53 – 3.47 (m, 4H), 3.36 (s, 3H), 2.69 (t, *J* = 7.2 Hz, 2H), 1.94 (tt, *J* = 7.2, 6.1 Hz, 2H), 1.77 – 1.66 (m, 4H).

^{13}C NMR (151 MHz, $CDCl_3$) δ 199.9, 145.5, 138.6, 138.5, 130.1, 128.48, 128.47, 127.72, 127.71, 127.67 (2C), 94.8, 75.4, 73.05, 72.95, 70.0, 69.4, 55.8, 37.2, 31.8, 25.6, 24.1.

IR (thin film): 3028, 2928, 2860, 1697, 1674, 1633, 1453, 1361, 1150, 1095, 1028, 983, 917, 737, 698 cm^{-1}.

HRMS (APCI−): *m/z* Calc. for $C_{26}H_{33}O_5$ $[M-H]^-$: 425.2334, found: 425.2331.

TLC: R_f = 0.29 (hexanes: EtOAc = 4:1, anisaldehyde, UV active).

Table S1. Condition and substrate optimization for the epoxidation of 23.

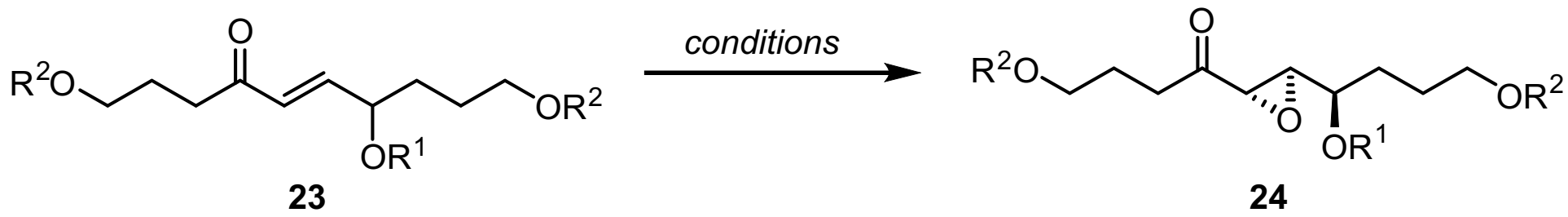

Entry	R^1	R^2	Condition	Yield[a]	dr[b]
1	TBDPS	Bn	H_2O_2, Triton B/NaOH, 0 to 23 °C	62%	1:1
2	TBDPS	Bn	Ph_3COOH, *n*-BuLi, THF, −78 to 23 °C	N.R.	/
3	Bn	TBS	$ZnEt_2$, O_2, toluene/hexanes, 0 to 23 °C	83%	7.8:1
4	MOM	TBS	$ZnEt_2$, O_2, toluene/hexanes, 0 to 23 °C	60%	>20:1
5	MOM	Bn	$ZnEt_2$, O_2, toluene/hexanes, 0 to 23 °C	73%	>20:1
6	TBDPS	Bn	$ZnEt_2$, O_2, toluene/hexanes, 0 to 23 °C	93%	3.3:1
7	TBS	Bn	$ZnEt_2$, O_2, toluene/hexanes, 0 to 23 °C	75%	3.6:1
8	TIPS	Bn	$ZnEt_2$, O_2, toluene/hexanes, 0 to 23 °C	83%	2.3:1

[a]Isolation yield after silica gel chromatography. [b]Ratio determined by ^{1}H NMR.

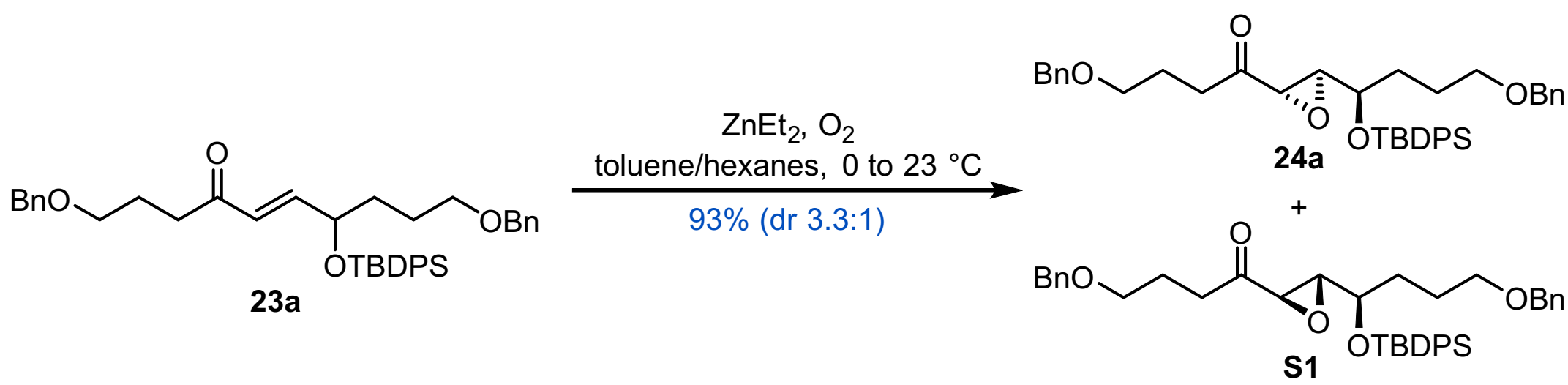

Under an oxygen atmosphere, diethylzinc (3.0 equiv, 1.0 M in hexanes, 138 mL, 138.0 mmol) was added *carefully* and dropwise at 0 °C to a round-bottom flask charged with a solution of enone **23a** (1.0 equiv, 28.57 g, 46.01 mmol) in toluene (225 mL).[3] After completion of the addition, the reaction mixture was directly warmed to room temperature and stirred vigorously for 24 h. The reaction was then quenched with *sat.* NH_4Cl aq. (800 mL). The aqueous layer was extracted with CH_2Cl_2 (600 mL × 3), and the combined organic layers were dried over sodium sulfate. The mixture was then filtered successively through a plug of Celite and silica gel using a fritted funnel, washed with CH_2Cl_2, and concentrated *in vacuo*. The residue was purified by silica gel chromatography (hexanes/EtOAc = 10:1) to afford a mixture of epoxide **24a** and diastereomer **S1** as a colorless gum (27.17 g, 93%, dr 3.3:1).

Safety note: The reaction flask and stir bar must be thoroughly cleaned before use. Any residual metal contamination may promote rapid decomposition of peroxide intermediates, which could result in violent decomposition and a potential explosion! Efficient cooling must be maintained throughout the addition of diethylzinc to prevent uncontrolled exotherm and peroxide decomposition. A suitable blast shield must be in place between the reaction apparatus and the operator throughout the reaction.

Compound 24a and S1 (inseparable mixture of diastereomers, dr 3.3:1)**:**

^{1}H NMR (400 MHz, CDCl$_3$) δ 7.73 – 7.66 (m, 4H), 7.43 – 7.28 (m, 16H), 4.48 (s, 2H), 4.44 (s, 2H), 3.64 (app q, J = 5.0 Hz, 1H), 3.48 – 3.41 (m, 2H), 3.40 – 3.29 (m, 2H), 3.24 (d, J = 2.1 Hz, 0.23H), 3.15 (dd, J = 6.1, 2.0 Hz, 0.23H), 3.14 (d, J = 2.0 Hz, 0.77H), 3.08 (dd, J = 5.1, 1.9 Hz, 0.77H), 2.38 – 2.22 (m, 2H), 1.90 – 1.77 (m, 2H), 1.74 – 1.52 (m, 4H), 1.10 (s, 9 × 0.19H), 1.05 (s, 9 × 0.81H).

^{13}C NMR (101 MHz, CDCl$_3$) δ 206.9*, 206.6, 138.7, 138.6*, 138.50, 138.48*, 136.1, 136.02*, 135.98, 134.9*, 133.8*, 133.49*, 133.47, 133.2, 130.1, 130.0, 129.9*, 128.51, 128.46, 127.9, 127.84, 127.76, 127.73*, 127.71, 127.69*, 127.67, 127.6, 73.6*, 73.0, 72.9, 71.4, 70.2, 70.0*, 69.18*, 69.15, 61.0*, 60.4, 57.6*, 57.4, 34.5, 33.9*, 31.8, 31.5*, 27.1*, 27.0, 26.7*, 25.1*, 24.5, 23.3, 19.6*, 19.5.

**Signals assigned to the minor diastereomer when resolved; additional minor resonances overlap with the major diastereomer.*

IR (thin film): 2933, 2858, 1712, 1428, 1362, 1265, 1103, 736, 702 cm^{-1}.

HRMS (APCI+): *m/z* Calc. for $C_{40}H_{49}O_5Si$ $[M+H]^+$: 637.3344, found: 637.3351.

TLC: R_f = 0.54 (hexanes: EtOAc = 4:1, anisaldehyde, UV active).

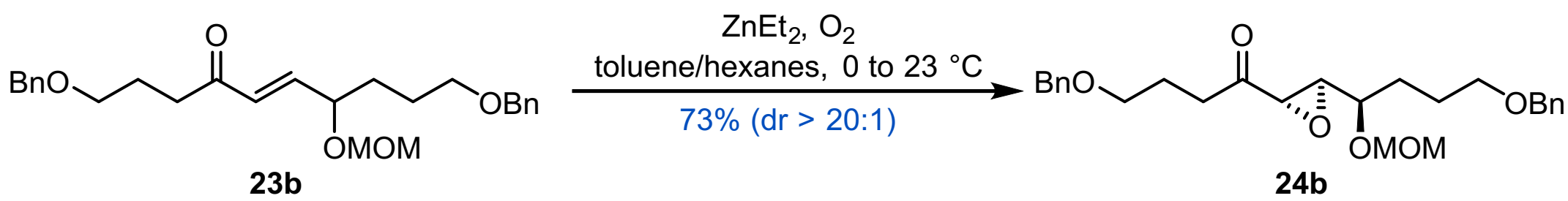

Under an oxygen atmosphere, diethylzinc (3.0 equiv, 1.0 M in hexanes, 0.62 mL, 0.621 mmol) was added *carefully* and dropwise at 0 °C to a round-bottom flask charged with a solution of enone **23b** (1.0 equiv, 88.3 mg, 0.207 mmol) in toluene (1 mL).[3] After completion of the addition, the reaction mixture was directly warmed to room temperature and stirred vigorously for 24 h. The reaction was then quenched with *sat.* NH_4Cl aq. (3 mL) and diluted with water (5 mL). The aqueous layer was extracted with CH_2Cl_2 (10 mL × 3), and the combined organic layers were dried over sodium sulfate. The mixture was then filtered successively through

plugs of Celite and silica gel using a funnel, washed with CH_2Cl_2, and concentrated in vacuo. The residue was purified by silica gel chromatography (hexanes/EtOAc = 8:1 → 5:1) to afford epoxide **24b** as a colorless oil (66.6 mg, 73%, dr >20:1).

Safety note: The reaction flask and stir bar must be thoroughly cleaned before use. Any residual metal contamination may promote rapid decomposition of peroxide intermediates, which could result in violent decomposition and a potential explosion! Efficient cooling must be maintained throughout the addition of diethylzinc to prevent uncontrolled exotherm and peroxide decomposition. A suitable blast shield must be in place between the reaction apparatus and the operator throughout the reaction.

Compound 24b:

^{1}H NMR (400 MHz, CDCl$_3$) δ 7.37 – 7.26 (m, 10H), 4.66 (d, *J* = 6.9 Hz, 1H), 4.57 (d, *J* = 6.9 Hz, 1H), 4.50 (s, 2H), 4.47 (s, 2H), 3.54 – 3.44 (m, 6H), 3.33 (s, 3H), 3.04 (dd, *J* = 5.1, 2.0 Hz, 1H), 2.61 – 2.42 (m, 2H), 1.91 (tt, *J* = 7.1, 6.0 Hz, 2H), 1.83 – 1.64 (m, 4H).

^{13}C NMR (101 MHz, CDCl$_3$) δ 206.8, 138.6, 138.4, 128.5 (2C), 127.8 (2C), 127.73, 127.70, 96.4, 75.4, 73.1, 73.0, 70.0, 69.2, 59.4, 57.8, 55.8, 34.4, 29.7, 25.4, 23.4.

IR (thin film): 3029, 2931, 2856, 1712, 1454, 1362, 1154, 1100, 1033, 994, 738, 700 cm^{-1}.

HRMS (APCI−): *m/z* Calc. for $C_{26}H_{33}O_6$ [M−H]$^-$: 441.2283, found: 441.2283.

TLC: R$_f$ = 0.32 (hexanes: EtOAc = 4:1, anisaldehyde, UV active).

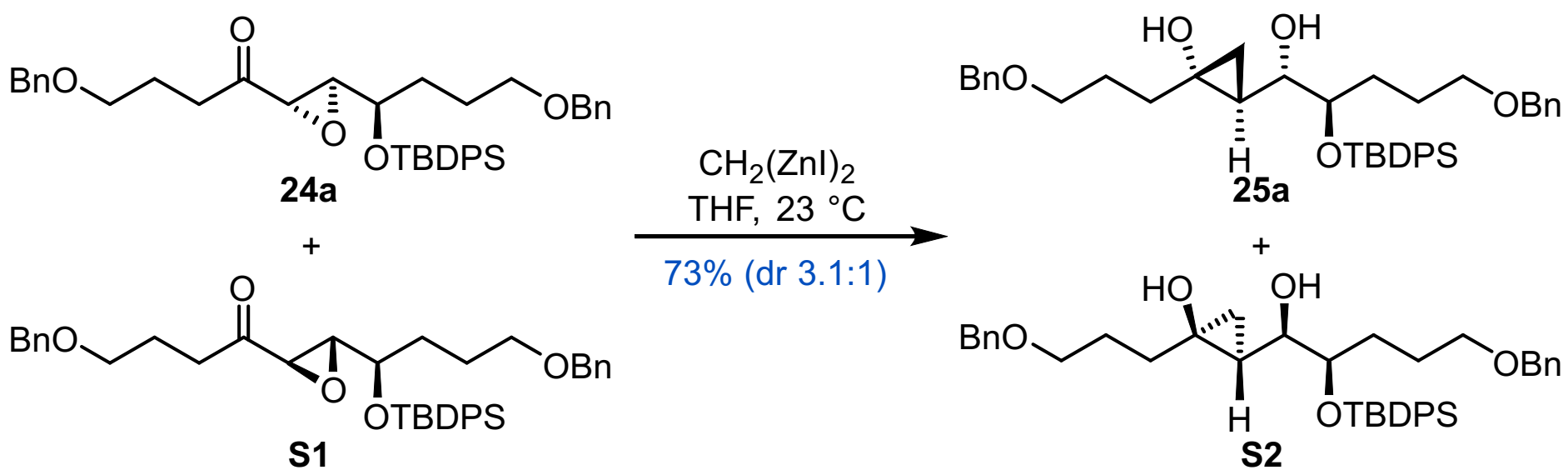

Preparation of bis(iodozincio)methane solution:[4]

A 20 mL microwave vial was charged with zinc powder (2.3 equiv, washed with HCl aq., 1.23 g, 18.75 mmol) and lead(II) chloride (0.0035 equiv, 8.0 mg, 0.029 mmol). THF (1.5 mL) and diiodomethane (0.1 equiv, 0.06 mL, 0.75 mmol) were then added sequentially. After the addition, the mixture was sonicated for 1 h in an ultrasonic cleaner bath under an argon atmosphere. A solution of diiodomethane (0.9 equiv, 0.60 mL, 7.5 mmol) in THF (15 mL) was then added slowly via syringe pump over 1.5 h at 0 °C with vigorous stirring. Upon

completion of the addition, the mixture was gradually warmed to room temperature and stirred overnight. After stirring was stopped, the reaction vessel was allowed to stand undisturbed for several hours. The resulting clear solution was titrated with iodine before use (normally 0.25–0.35 M).

Cyclopropanation of epoxide mixture of 24a and S1:

Under argon, a solution of bis(iodozincio)methane (3.0 equiv, average 0.306 M in THF, 27.0 mL, 8.25 mmol) was added to a round-bottom flask charged with a mixture of epoxide **24a** and **S1** (1.0 equiv, dr = 3.3:1, 1.75 g, 2.75 mmol). After 3 h, the reaction was carefully quenched with *sat.* $NaHCO_3$ aq. (30 mL) and water (30 mL). The resulting white suspension was extracted with CH_2Cl_2 (80 mL × 3), and the combined organic layers were dried over sodium sulfate, filtered, and concentrated *in vacuo*. Three additional batches were run in parallel (1.80 g, 1.39 g, 1.36 g). The crude products were combined and purified by silica gel chromatography (hexanes/EtOAc = 10:1 → 3:1 → 2:1, containing 1% triethylamine) to afford cyclopropanol **25a** (3.55 g, 55%) and **S2** (1.16 g, 18%), both as pale-yellow oils.

Compound 25a:

^{1}H NMR (400 MHz, $CDCl_3$) δ 7.76 – 7.65 (m, 4H), 7.43 – 7.24 (m, 16H), 4.52 (s, 2H), 4.37 (s, 2H), 3.86 (td, *J* = 5.7, 2.9 Hz, 1H), 3.67 – 3.43 (m, 4H), 3.24 (t, *J* = 5.7 Hz, 2H), 2.49 (br s, 1H), 1.82 (p, *J* = 6.7 Hz, 2H), 1.74 – 1.45 (m, 6H), 1.09 (s, 9H), 0.90 (td, *J* = 9.2, 6.2 Hz, 1H), 0.55 (dd, *J* = 9.3, 5.5 Hz, 1H), 0.45 (t, *J* = 5.9 Hz, 1H).

^{13}C NMR (101 MHz, $CDCl_3$) δ 138.7, 138.1, 136.1, 136.0, 134.1, 133.9, 129.82, 129.75, 128.5, 128.4, 127.85, 127.77, 127.7, 127.64, 127.60, 127.5, 76.5, 75.3, 73.1, 72.7, 70.5, 70.3, 57.5, 36.7, 29.1, 27.3, 26.6, 25.9, 24.7, 19.6, 18.5.

IR (thin film): 3388, 3032, 2932, 2856, 1453, 1427, 1362, 1105, 739, 703 cm^{-1}.

HRMS (APCI−): *m/z* Calc. for $C_{41}H_{51}O_5Si$ $[M-H]^-$: 651.3511, found: 651.3514.

TLC: R_f = 0.61 (hexanes: EtOAc = 1:1, anisaldehyde, UV active).

Compound S2:

^{1}H NMR (400 MHz, $CDCl_3$) δ 7.74 – 7.65 (m, 4H), 7.42 – 7.24 (m, 16H), 4.52 (d, *J* = 2.5 Hz, 2H), 4.35 (s, 2H), 3.83 (dt, *J* = 7.4, 3.9 Hz, 1H), 3.65 – 3.50 (m, 3H), 3.48 (dd, *J* = 9.0, 4.3 Hz, 1H), 3.25 – 3.12 (m, 2H), 2.65 (br s, 1H), 1.82 (p, *J* = 6.7 Hz, 2H), 1.71 – 1.44 (m, 6H), 1.08 (s, 9H), 0.84 (td, *J* = 9.2, 6.2 Hz, 1H), 0.55 (t, *J* = 5.9 Hz, 1H), 0.45 (dd, *J* = 9.3, 5.6 Hz, 1H).

^{13}C NMR (101 MHz, CDCl$_3$) δ 138.7, 138.2, 136.1, 136.0, 134.1, 133.7, 129.84, 129.79, 128.5, 128.4, 127.9, 127.76 (2C), 127.65, 127.59, 127.5, 76.7, 74.4, 73.1, 72.7, 70.4, 70.2, 58.3, 36.5, 30.2, 27.3, 26.9, 26.5, 25.3, 19.7, 18.3.

IR (thin film): 3390, 2932, 2856, 1453, 1428, 1362, 1104, 913, 736, 701 cm^{-1}.

HRMS (APCI−): *m/z* Calc. for $C_{41}H_{51}O_5Si$ [M−H]$^-$: 651.3511, found: 651.3511.

TLC: R$_f$= 0.57 (hexanes: EtOAc = 1:1, anisaldehyde, UV active).

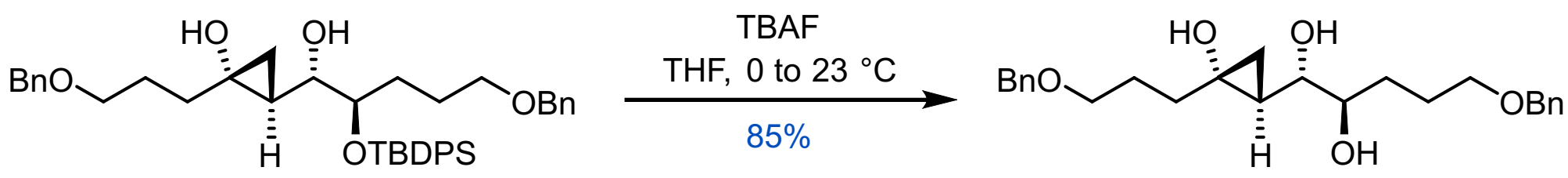

25a **26**

Under argon, tetra-*n*-butylammonium fluoride (1.25 equiv, 1.0 M in THF, 4.33 mL, 4.33 mmol) was added at 0 °C to a round-bottom flask charged with a solution of **25a** (1.0 equiv, 2.26 g, 3.46 mmol) in THF (69 mL). The reaction mixture was then allowed to warm gradually to room temperature. After 6 h, water (70 mL) was added, and the mixture was transferred to a separatory funnel. The aqueous layer was extracted with CH_2Cl_2 (100 mL × 3), and the combined organic layers were dried over sodium sulfate, filtered, and concentrated *in vacuo*. The residue was purified by silica gel chromatography (hexanes/EtOAc = 1:1 → EtOAc/MeOH = 100:1, containing 1% triethylamine) to afford triol **26** as a pale-yellow oil (1.22 g, 85%).

Compound 26:

^{1}H NMR (400 MHz, CDCl$_3$) δ 7.40 – 7.21 (m, 10H), 4.52 (s, 2H), 4.51 (s, 2H), 4.36 (br s, 1H), 3.75 (dt, *J* = 9.6, 3.3 Hz, 1H), 3.61 – 3.54 (m, 3H), 3.52 (t, *J* = 6.0 Hz, 2H), 3.43 (br s, 2H), 1.90 – 1.79 (m, 3H), 1.78 – 1.52 (m, 5H), 0.95 (td, *J* = 9.0, 6.3 Hz, 1H), 0.77 – 0.58 (m, 2H).

^{13}C NMR (101 MHz, CDCl$_3$) δ 138.3, 137.8, 128.5, 128.4, 127.9, 127.8, 127.7, 127.6, 75.0, 74.4, 73.1, 72.9, 70.52, 70.45, 57.3, 36.6, 29.2, 26.58, 26.56, 24.2, 18.0.

IR (thin film): 3386, 2923, 2857, 1451, 1363, 1205, 1096, 1026, 912, 737, 698 cm^{-1}.

HRMS (APCI−): *m/z* Calc. for $C_{25}H_{33}O_5$ [M−H]$^-$: 413.2334, found: 413.2340.

TLC: R$_f$= 0.05 (hexanes: EtOAc = 1:1, anisaldehyde, UV active).

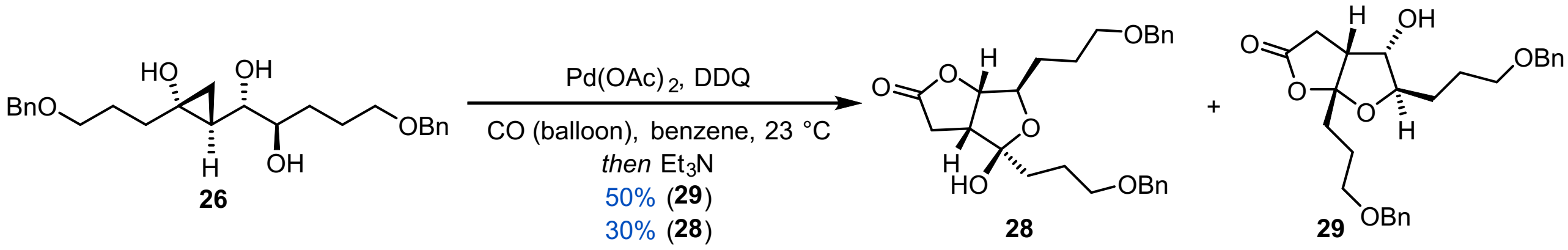

To a round-bottom flask charged with a solution of **26** (1.0 equiv, 1.59 g, 3.84 mmol) in benzene (390 mL) were rapidly added 2,3-dichloro-5,6-dicyano-1,4-benzoquinone (2.0 equiv, 1.74 g, 7.67 mmol) and palladium(II) acetate (10 mol%, 86.1 mg, 0.384 mmol).[5] The reaction mixture was stirred vigorously at room temperature overnight (~14 h). After complete consumption of the cyclopropanols, triethylamine (13.6 mL) was added, and the mixture was stirred for 1.5 h. The resulting black solution was filtered through a plug of Celite layered over silica gel. The plug was flushed with ethyl acetate (~1 L), and the combined filtrate was concentrated *in vacuo*. The residue was purified by silica gel column chromatography (hexanes/EtOAc = 3:1 → 2:1 → 1:1) to afford hemiacetal **28** as a yellow oil (507.4 mg, 30%) and alcohol **29** as a blue oil (0.85 g, 50%).

Compound 28:

^{1}H NMR (400 MHz, $CDCl_3$) δ 7.38 – 7.26 (m, 10H), 4.56 (dd, *J* = 8.0, 3.6 Hz, 1H), 4.53 (d, *J* = 4.8 Hz, 2H), 4.50 (s, 2H), 4.33 (d, *J* = 1.5 Hz, 1H), 4.28 – 4.20 (m, 1H), 3.57 – 3.44 (m, 4H), 2.76 – 2.68 (m, 1H), 2.66 (dd, *J* = 17.7, 1.7 Hz, 1H), 2.49 (dd, *J* = 17.7, 9.3 Hz, 1H), 1.92 – 1.70 (m, 8H).

^{13}C NMR (101 MHz, $CDCl_3$) δ 177.2, 138.5, 137.6, 128.6, 128.4, 127.9, 127.7, 127.6, 104.8, 87.4, 82.3, 73.1, 73.0, 70.2, 69.8, 47.3, 37.5, 30.1, 30.0, 25.8, 23.9.

IR (thin film): 3385, 2937, 2860, 1776, 1452, 1363, 1162, 1097, 953, 740, 700 cm^{-1}.

HRMS (APCI−): *m/z* Calc. for $C_{26}H_{31}O_6$ [M−H]$^-$: 439.2126, found: 439.2138.

TLC: R_f = 0.50 (hexanes: EtOAc = 1:1, anisaldehyde, UV active).

Compound 29:

^{1}H NMR (400 MHz, $CDCl_3$) δ 7.42 – 7.26 (m, 10H), 4.49 (s, 4H), 3.87 (td, *J* = 8.5, 2.8 Hz, 1H), 3.77 (dt, *J* = 8.3, 6.0 Hz, 1H), 3.62 – 3.47 (m, 4H), 3.11 (br s, 1H), 3.01 (dd, *J* = 18.9, 3.3 Hz, 1H), 2.84 (ddd, *J* = 10.6, 8.6, 3.3 Hz, 1H), 2.49 (dd, *J* = 19.0, 10.7 Hz, 1H), 2.00 – 1.89 (m, 2H), 1.87 – 1.68 (m, 6H).

^{13}C NMR (101 MHz, $CDCl_3$) δ 175.3, 138.5, 137.9, 128.7, 128.5, 128.1, 128.0, 127.82, 127.79, 116.9, 81.4, 74.2, 73.4, 73.2, 70.5, 69.8, 45.4, 34.9, 30.0, 28.9, 25.5, 24.0.

IR (thin film): 3424, 2861, 1770, 1452, 1363, 1270, 1205, 1102, 918, 775, 702 cm^{-1}.

HRMS (APCI−): *m/z* Calc. for $C_{26}H_{31}O_6$ [M−H]$^-$: 439.2126, found: 439.2128.

TLC: R_f = 0.25 (hexanes: EtOAc = 1:1, anisaldehyde, UV active).

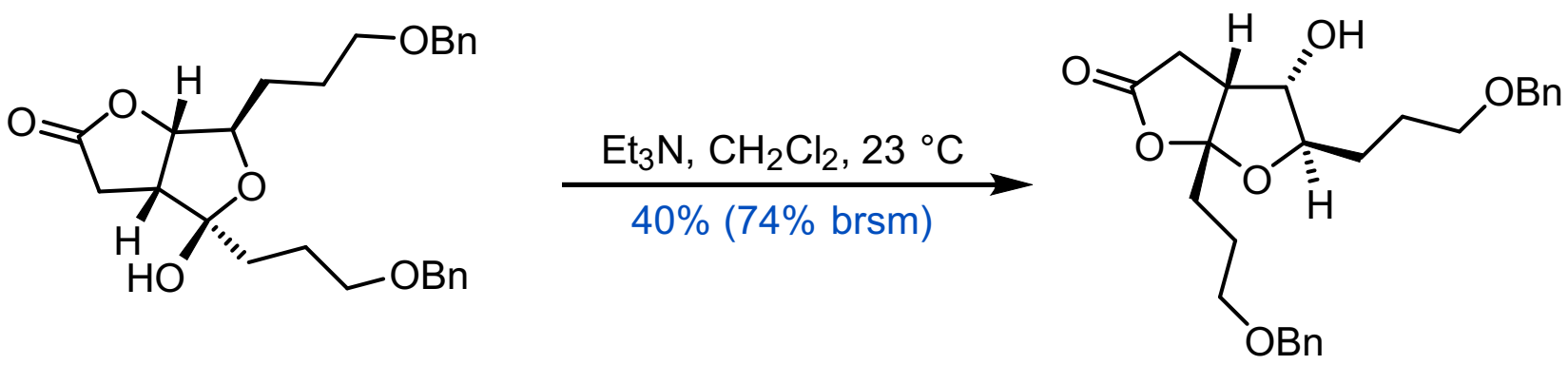

28 **29**

A round-bottom flask was charged with **28** (1.0 equiv, 829.7 mg, 1.88 mmol), CH_2Cl_2 (188 mL), and triethylamine (25.0 equiv, 6.6 mL, 47.08 mmol). The reaction mixture was stirred at room temperature overnight (~14 h). The solution was then concentrated *in vacuo*, and the residue was purified by silica gel column chromatography (hexanes/EtOAc = 3:1 → 1:1) to afford alcohol **29** as a yellow oil (331.7 mg, 40%), along with recovered hemiacetal **28** (382.7 mg, 46%).

Note: The analytical data matches the one described above.

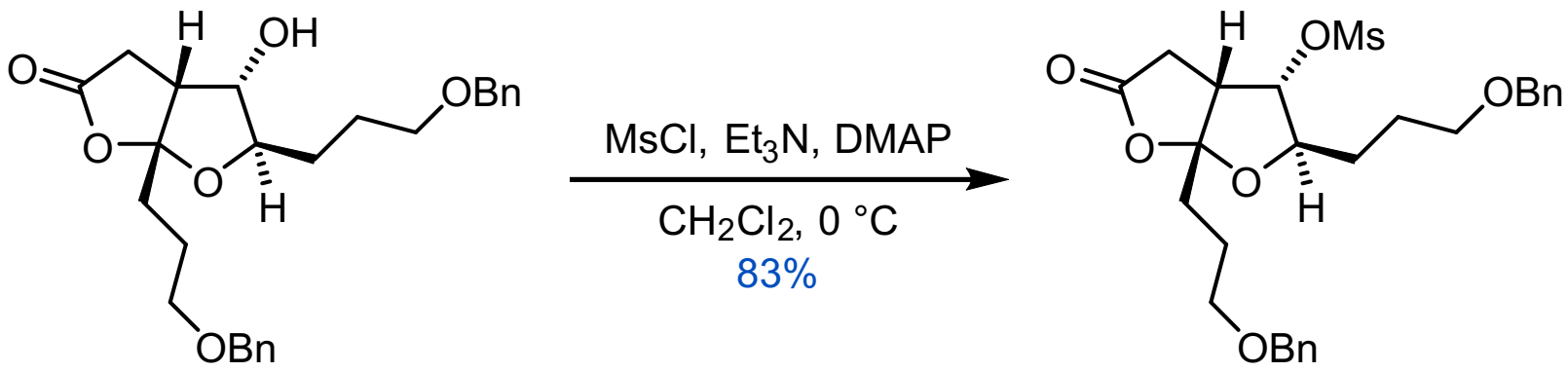

29 **30**

Under argon, methanesulfonyl chloride (2.0 equiv, 0.41 mL, 5.36 mmol) was added at 0 °C to a round-bottom flask charged with a solution of **29** (1.0 equiv, 1.18 g, 2.68 mmol), 4-dimethylaminopyridine (0.2 equiv, 65.4 mg, 0.536 mmol), and triethylamine (3.0 equiv, 1.12 mL, 8.04 mmol) in CH_2Cl_2 (54 mL). After 2 h, *sat.* $NaHCO_3$ aq. (108 mL) was added, and the mixture was transferred to a separatory funnel. The aqueous layer was extracted with CH_2Cl_2 (100 mL × 3), and the combined organic layers were dried over sodium sulfate, filtered, and concentrated *in vacuo*. The residue was purified by silica gel chromatography (hexanes/EtOAc = 3:1 → 2:1) to afford mesylate **30** as a yellow solid (1.16 g, 83%).

Compound 30:

^{1}H NMR (400 MHz, $CDCl_3$) δ 7.41 – 7.26 (m, 10H), 4.68 (t, J = 8.2 Hz, 1H), 4.49 (d, J = 1.3 Hz, 4H), 3.98 (td, J = 7.7, 3.8 Hz, 1H), 3.50 (td, J = 6.1, 1.7 Hz, 4H), 3.15 (ddd, J = 10.5, 8.5, 3.3 Hz, 1H), 3.04 (s, 3H), 2.94 (dd, J = 19.1, 3.3 Hz, 1H), 2.72 (dd, J = 19.1, 10.5 Hz, 1H), 2.05 – 1.65 (m, 8H).

^{13}C NMR (101 MHz, $CDCl_3$) δ 173.7, 138.4, 138.3, 128.54, 128.51, 127.8 (3C), 127.7, 116.4, 79.3, 78.8, 73.1,

73.0, 69.5 (2C), 44.2, 38.3, 34.8, 29.9, 28.5, 25.6, 23.8.

IR (thin film): 3028, 2935, 2860, 1777, 1452, 1360, 1264, 1178, 1029, 968, 931, 845, 744, 700 cm^{-1}.

HRMS (APCI−): *m/z* Calc. for $C_{27}H_{33}O_8S$ $[M-H]^-$: 517.1902, found: 517.1911.

TLC: R_f = 0.43 (hexanes: EtOAc = 1:1, anisaldehyde, UV active).

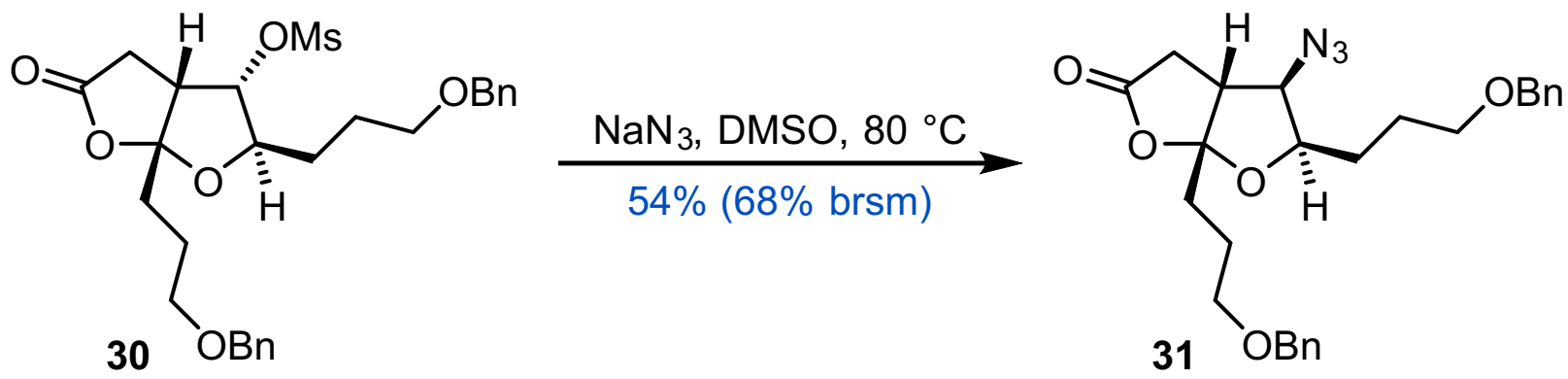

A round-bottom flask was charged with **30** (1.0 equiv, 3.02 g, 5.82 mmol), sodium azide (20.0 equiv, 7.57 g, 116.5 mmol), and dimethyl sulfoxide (100 mL). The suspension was then heated to 80 °C. After 48 h, the flask was allowed to cool to room temperature, and water (400 mL) was added. The mixture was extracted with ethyl acetate (300 mL × 3), and the combined organic layers were washed with brine (500 mL), dried over sodium sulfate, filtered, and concentrated *in vacuo*. The residue was purified by silica gel chromatography (hexanes/EtOAc = 4:1 → 2:1) to afford azide **31** as a colorless oil (1.47 g, 54%), along with recovered mesylate **30** (0.64 g, 21%).

Compound 31:

^{1}H NMR (400 MHz, $CDCl_3$) δ 7.40 – 7.26 (m, 10H), 4.50 (s, 2H), 4.49 (s, 2H), 4.09 (ddd, *J* = 7.4, 5.7, 3.3 Hz, 1H), 3.69 (d, *J* = 3.3 Hz, 1H), 3.58 – 3.47 (m, 4H), 3.02 – 2.90 (m, 2H), 2.49 – 2.36 (m, 1H), 2.11 – 1.97 (m, 2H), 1.92 – 1.65 (m, 6H).

^{13}C NMR (101 MHz, $CDCl_3$) δ 173.6, 138.5 (2C), 128.6, 128.5, 127.9, 127.83, 127.81, 127.76, 117.7, 80.6, 73.2, 73.1, 69.9, 69.8, 69.7, 48.7, 34.5, 34.3, 26.4, 26.1, 24.0.

IR (thin film): 2931, 2860, 2102, 1776, 1452, 1360, 1261, 1100, 959, 766 cm^{-1}.

HRMS (ESI+): *m/z* Calc. for $C_{26}H_{32}O_5N_3$ $[M+H]^+$: 466.2337, found: 466.2349.

TLC: R_f = 0.64 (hexanes: EtOAc = 1:1, anisaldehyde, UV active).

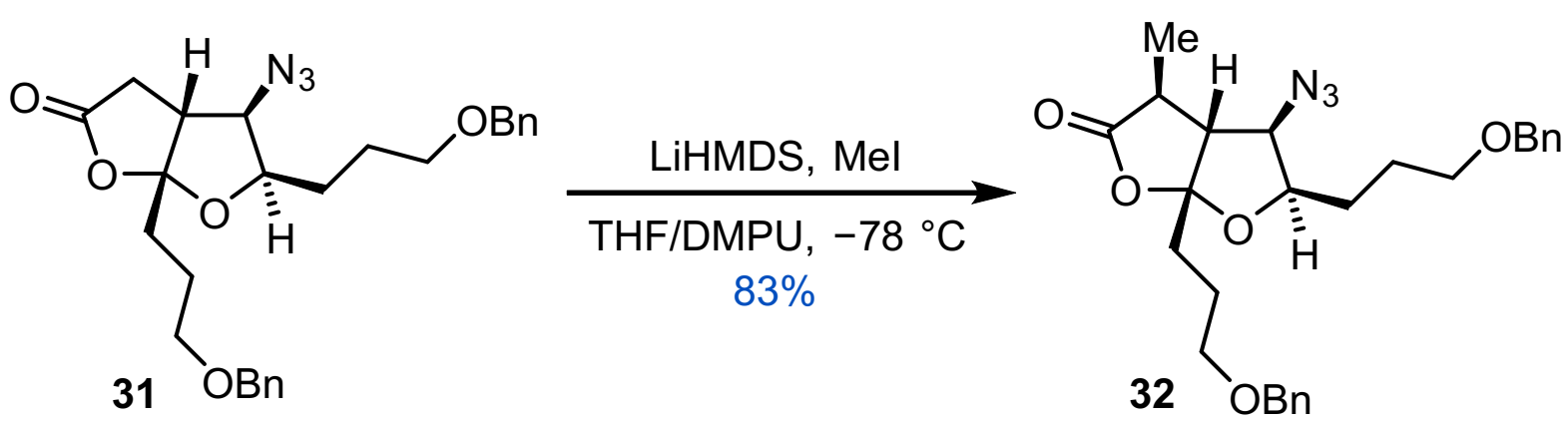

Under argon, lithium bis(trimethylsilyl)amide (1.2 equiv, 1.0 M in THF, 3.8 mL, 3.80 mmol) was added dropwise at −78 °C to a round-bottom flask charged with a solution of **31** (1.0 equiv, 1.47 g, 3.16 mmol) in THF (32 mL) and *N*,*N*′-dimethylpropyleneurea (5.3 mL). After 1 h, iodomethane (1.1 equiv, 0.22 mL, 3.47 mmol) was added, and the reaction mixture was stirred at this temperature for 4 h. *Sat.* NH_4Cl aq. (75 mL) and water (45 mL) were then added, and the mixture was extracted with ethyl acetate (100 mL × 3). The combined organic layers were dried over sodium sulfate, filtered, and concentrated *in vacuo*. The residue was purified by silica gel chromatography (hexanes/EtOAc = 5:1 → 4:1) to afford azide **32** as a colorless oil (1.24 g, 83%).

Compound 32:

^{1}H NMR (600 MHz, $CDCl_3$) δ 7.40 – 7.24 (m, 10H), 4.50 (s, 2H), 4.48 (d, *J* = 2.0 Hz, 2H), 4.10 (ddd, *J* = 7.2, 5.8, 3.0 Hz, 1H), 3.75 (d, *J* = 3.0 Hz, 1H), 3.55 – 3.49 (m, 4H), 2.55 (d, *J* = 5.0 Hz, 1H), 2.46 (qd, *J* = 7.5, 5.1 Hz, 1H), 2.06 – 1.96 (m, 2H), 1.88 – 1.68 (m, 6H), 1.36 (d, *J* = 7.7 Hz, 3H).

^{13}C NMR (151 MHz, $CDCl_3$) δ 176.9, 138.5, 138.4, 128.6, 128.5, 127.9, 127.80, 127.78, 127.7, 115.8, 80.9, 73.15, 73.06, 69.9, 69.8, 69.3, 56.5, 40.7, 35.3, 26.4, 26.2, 24.2, 17.6.

IR (thin film): 2935, 2861, 2101, 1773, 1453, 1360, 1271, 1202, 1101, 935, 777, 701 cm^{-1}.

HRMS (APCI+): *m/z* Calc. for $C_{27}H_{34}O_5N_3$ $[M+H]^+$: 480.2493, found: 480.2492.

TLC: R_f = 0.71 (hexanes: EtOAc = 1:1, anisaldehyde, UV active).

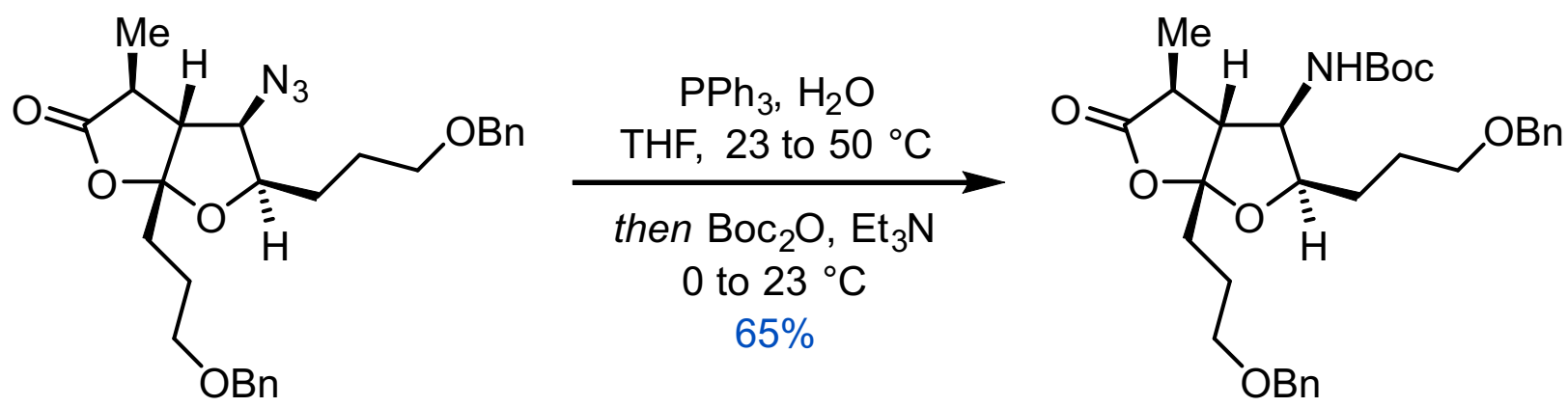

32 **33**

Under argon, triphenylphosphine (1.5 equiv, 411.8 mg, 1.57 mmol) was added at room temperature to a microwave vial charged with a solution of **32** (1.0 equiv, 502.0 mg, 1.05 mmol) in THF (10.5 mL). After 2 h, water (4.0 equiv, 75.5 *μ*L, 4.19 mmol) was added, and the vial was heated to 50 °C. Upon complete consumption of the *N*-ylide (~24 h), the mixture was cooled to 0 °C, and triethylamine (10.0 equiv, 1.46 mL, 10.47 mmol) and di-*tert*-butyl dicarbonate (6.0 equiv, 1.37 g, 6.28 mmol) were added. The reaction mixture was then allowed to warm slowly to room temperature and stirred overnight. *Sat.* $NaHCO_3$ aq. (20 mL) was then added, and the mixture was extracted with CH_2Cl_2 (25 mL × 3). The combined organic layers were dried over sodium sulfate, filtered, and concentrated *in vacuo*. The residue was purified by silica gel chromatography (hexanes/EtOAc =

6:1 → 4:1) to afford **33** as a colorless oil (376.9 mg, 65%).

Compound 33:

^{1}H NMR (400 MHz, $CDCl_3$) δ 7.39 – 7.26 (m, 10H), 4.74 (d, *J* = 9.0 Hz, 1H), 4.50 (s, 2H), 4.48 (s, 2H), 4.02 (td, *J* = 6.3, 3.4 Hz, 1H), 3.93 (dd, *J* = 9.3, 3.5 Hz, 1H), 3.59 – 3.43 (m, 4H), 2.64 – 2.50 (m, 1H), 2.39 (d, *J* = 4.7 Hz, 1H), 2.04 – 1.90 (m, 2H), 1.84 – 1.64 (m, 6H), 1.44 (s, 9H), 1.37 (d, *J* = 7.5 Hz, 3H).

^{13}C NMR (101 MHz, $CDCl_3$) δ 177.8, 155.3, 138.5, 138.4, 128.6, 128.5, 127.9, 127.8 (2C), 127.7, 115.3, 80.4, 79.9, 73.2, 73.0, 69.8 (2C), 59.4, 58.3, 41.2, 36.2, 28.5, 26.3, 25.6, 24.3, 17.7.

IR (thin film): 3345, 2934, 2862, 1772, 1707, 1501, 1455, 1366, 1251, 1166, 1102, 936, 768 cm^{-1}.

HRMS (APCI−): *m/z* Calc. for $C_{32}H_{43}O_7NCl$ $[M+Cl]^-$: 588.2734, found: 588.2731.

TLC: R_f = 0.79 (hexanes: EtOAc = 1:1, anisaldehyde, faintly UV active).

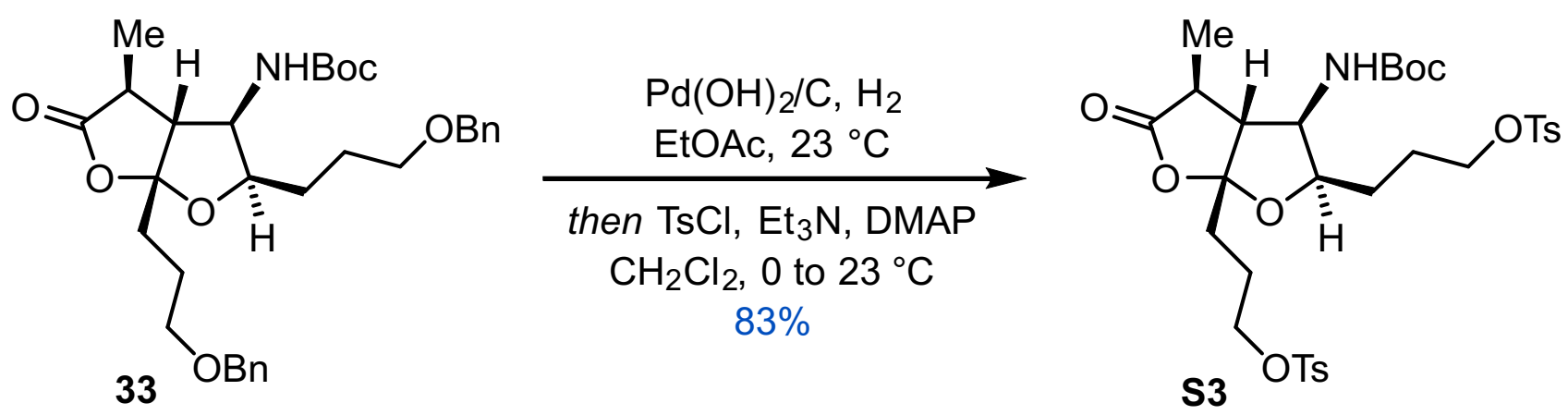

A microwave vial was charged with **33** (1.0 equiv, 376.9 mg, 0.68 mmol), palladium hydroxide on carbon (10 wt%, 10%, 72.2 mg), and ethyl acetate (5 mL) at room temperature. The atmosphere was then replaced with hydrogen using a balloon by five vacuum/backfill cycles, and the mixture was stirred vigorously at room temperature. After complete consumption of the starting material (~12 h), the atmosphere was switched back to argon, and the mixture was cooled to 0 °C. CH_2Cl_2 (35 mL), triethylamine (10.0 equiv, 0.95 mL, 6.81 mmol), 4-dimethylaminopyridine (0.3 equiv, 25.0 mg, 0.20 mmol), and *p*-toluenesulfonyl chloride (5.0 equiv, 649.2 mg, 3.41 mmol) were then added successively. The reaction mixture was allowed to warm slowly to room temperature and stirred overnight. *Sat.* $NaHCO_3$ aq. (30 mL) was then added, and the mixture was extracted with CH_2Cl_2 (30 mL × 3). The combined organic layers were dried over sodium sulfate, filtered, and concentrated *in vacuo*. The residue was purified by silica gel chromatography (hexanes/EtOAc = 4:1 → 2:1) to afford **S3** as a white foam (385.1 mg, 83%).

Compound S3:

^{1}H NMR (400 MHz, $CDCl_3$) δ 7.83 – 7.72 (m, 4H), 7.40 – 7.31 (m, 4H), 4.76 (d, *J* = 9.0 Hz, 1H), 4.19 – 4.01 (m, 4H), 3.96 – 3.84 (m, 2H), 2.58 (dq, *J* = 7.7, 4.1 Hz, 1H), 2.453 (s, 3H), 2.446 (s, 3H), 2.32 (d, *J* = 4.7 Hz,

1H), 1.95 – 1.67 (m, 8H), 1.47 (s, 9H), 1.38 (d, J = 7.6 Hz, 3H).

^{13}C NMR (101 MHz, CDCl$_3$) δ 177.3, 155.3, 145.1, 145.0, 133.08, 133.06, 130.11, 130.06, 128.0 (2C), 114.5, 80.7, 79.8, 70.1, 69.9, 59.4, 59.0, 41.1, 35.5, 28.5, 25.9, 24.8, 23.5, 21.79, 21.78, 17.8.

IR (thin film): 3381, 2973, 1773, 1704, 1511, 1456, 1358, 1250, 1175, 1097, 935, 792 cm^{-1}.

HRMS (APCI−): *m/z* Calc. for $C_{32}H_{43}O_{11}NClS_2$ [M+Cl]$^-$: 716.1972, found: 716.1949.

TLC: R_f = 0.58 (hexanes: EtOAc = 1:1, anisaldehyde, UV active).

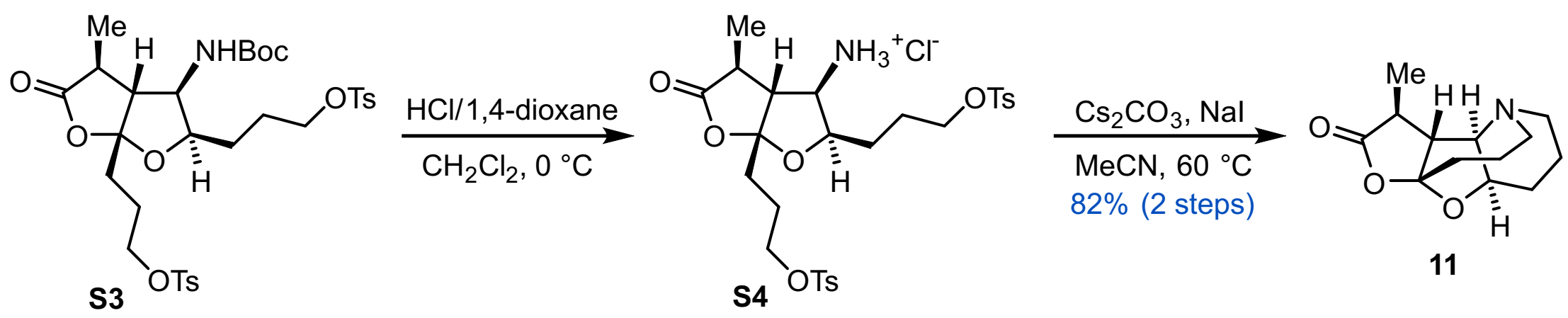

Under argon, hydrogen chloride solution (10.0 equiv, 4.0 M in 1,4-dioxane, 3.33 mL, 13.35 mmol) was added at room temperature to a round-bottom flask charged with a solution of **S3** (1.0 equiv, 0.91 g, 1.33 mmol) in CH_2Cl_2 (25 mL). After 1 h, the flask was transferred to a 4 °C refrigerator and stored overnight. The mixture was then concentrated directly *in vacuo* and dried under high vacuum to afford **S4** as a white foam. **S4** was used directly in the next step without further purification.

In a glovebox, cesium carbonate (6.0 equiv, 2.60 g, 7.98 mmol) and sodium iodide (10.0 equiv, 2.00 g, 13.35 mmol) were added to a round-bottom flask charged with a solution of **S4** (1.0 equiv, ~1.33 mmol) in MeCN (260 mL). The mixture was then heated to 60 °C. After 17 h, the reaction mixture was cooled to room temperature and filtered through Celite. The filtrate was concentrated, and water (120 mL) was added. The aqueous layer was extracted with CH_2Cl_2 (100 mL × 4), and the combined organic layers were dried over sodium sulfate, filtered through Celite, and concentrated *in vacuo*. The residue was purified by silica gel chromatography (CH_2Cl_2/MeOH = 40:1 → 20:1) to afford tertiary amine **11** as a colorless oil, which slowly crystallized at room temperature (257.7 mg, 82% over two steps).

Compound 11:

^{1}H NMR (400 MHz, CDCl$_3$) δ 4.18 (dt, J = 3.9, 2.3 Hz, 1H), 3.41 (s, 1H), 3.41 – 3.31 (m, 1H), 3.05 – 2.87 (m, 3H), 2.77 (d, J = 9.1 Hz, 1H), 2.65 (dq, J = 9.1, 7.1 Hz, 1H), 2.37 – 2.20 (m, 2H), 2.08 – 1.95 (m, 1H), 1.83 (qt, J = 12.5, 4.0 Hz, 1H), 1.75 – 1.57 (m, 3H), 1.34 (d, J = 7.1 Hz, 3H), 1.28 – 1.22 (m, 1H).

^{13}C NMR (101 MHz, CDCl$_3$) δ 178.3, 115.3, 76.1, 60.8, 54.9, 53.6, 53.0, 37.9, 34.9, 27.03, 26.98, 18.9, 16.1.

IR (thin film): 2932, 1773, 1453, 1361, 1308, 1255, 1201, 1111, 1072, 1026, 976, 943, 731 cm^{-1}.

HRMS (APCI+): *m/z* Calc. for $C_{13}H_{20}O_3N$ $[M+H]^+$: 238.1437, found: 238.1438.

TLC: R_f = 0.37 (CH_2Cl_2: MeOH = 10:1, Dragendorff's reagent, not UV active).

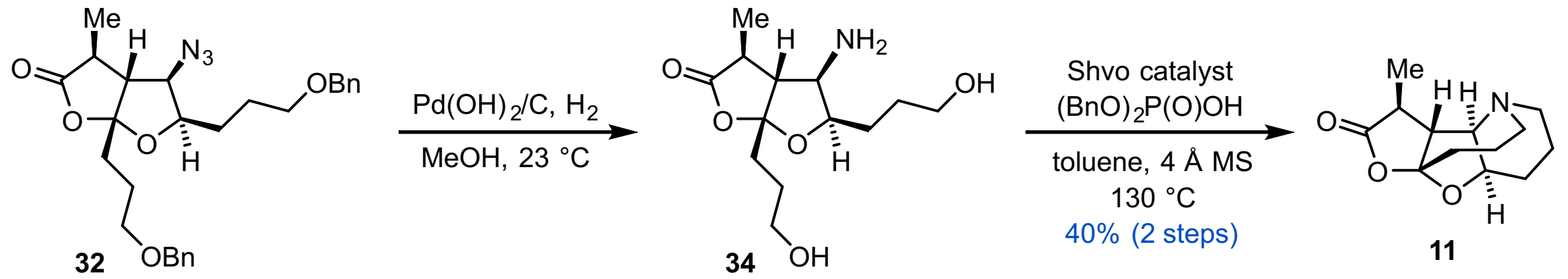

A round-bottom flask was charged with palladium hydroxide on carbon (0.5 equiv, 10%, 117.5 mg, 0.11 mmol) and a solution of **32** (1.0 equiv, 101.5 mg, 0.21 mmol) in methanol (20 mL). The atmosphere was then replaced with hydrogen using a balloon by five vacuum/backfill cycles, and the mixture was stirred vigorously at room temperature. After complete consumption of the azide (~24 h), the mixture was filtered through Celite and washed with methanol. The combined filtrate was concentrated *in vacuo* and dried under high vacuum to afford crude **34** (quantitative), which was used directly in the next step without further purification.

Compound 34 (crude)**:**

^{1}H NMR (400 MHz, MeOD) δ 4.12 (ddd, J = 7.7, 5.4, 3.3 Hz, 1H), 3.64 – 3.59 (m, 4H), 3.48 (d, J = 3.3 Hz, 1H), 2.85 (qd, J = 7.5, 4.6 Hz, 1H), 2.52 (d, J = 4.6 Hz, 1H), 2.12 – 1.92 (m, 2H), 1.76 – 1.61 (m, 6H), 1.38 (d, J = 7.5 Hz, 3H).

^{13}C NMR (101 MHz, MeOD) δ 179.7, 117.1, 81.5, 62.43, 62.40, 60.7, 59.0, 42.2, 36.4, 30.2, 28.0, 25.8, 17.6.

IR (thin film): 3351, 2937, 2874, 1756, 1455, 1379, 1325, 1273, 1190, 1056, 932 cm^{-1}.

HRMS (APCI+): *m/z* Calc. for $C_{13}H_{24}O_5N$ $[M+H]^+$: 274.1649, found: 274.1641.

TLC: R_f = 0.05 (CH_2Cl_2: MeOH = 10:1, PMA, not UV active).

In a glovebox, toluene (5 mL) was added to a sealed tube charged with crude **34** (1.0 equiv, 57.9 mg, ~0.21 mmol), Shvo catalyst (0.3 equiv, 69.0 mg, 63.6 μmol), dibenzyl phosphate (0.2 equiv, 11.8 mg, 42.4 μmol), and 4 Å molecular sieves (400 wt%, 230 mg).[6] The tube was then sealed, sonicated for 15 min, and heated to 130 °C for 48 h. After cooling to room temperature, the mixture was filtered through Celite and washed with CH_2Cl_2. The filtrate was concentrated *in vacuo*, and the residue was purified by silica gel chromatography

(CH_2Cl_2/MeOH = 40:1 → 20:1) and another time of preparative TLC (acetone/MeOH = 20:1) to afford tertiary amine **11** as a colorless oil (20.1 mg, 40% over two steps).

Note: The analytical data matches the one described above.

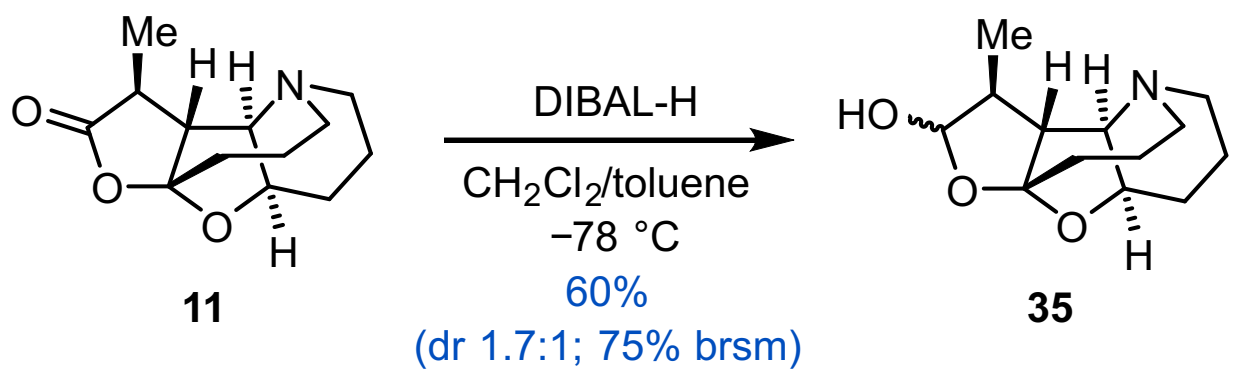

11 **35**

Under argon, diisobutylaluminum hydride (1.1 equiv, 1.0 M in toluene, 1.19 mL, 1.19 mmol) was added dropwise at −78 °C to a round-bottom flask charged with a solution of **11** (1.0 equiv, 257.7 mg, 1.09 mmol) in CH_2Cl_2 (25 mL). After 1 h, the reaction was carefully quenched with methanol (0.5 mL) and warmed to room temperature. *Sat.* potassium sodium tartrate aq. (20 mL) was then added, and the mixture was stirred for 3 h. The aqueous layer was extracted with CH_2Cl_2 (20 mL × 5), and the combined organic layers were dried over sodium sulfate, filtered, and concentrated *in vacuo*. The residue was purified by silica gel chromatography (CH_2Cl_2/MeOH = 30:1 → 20:1 → 10:1) to afford hemiacetal **35** as a white solid (156.9 mg, 60%, dr 1.7:1), along with recovered lactone **11** (52.7 mg, 20%).

Compound 35 (inseparable mixture of diastereomers, dr 1.7:1)**:**

^{1}H NMR (400 MHz, $CDCl_3$) δ 5.43 (d, J = 4.7 Hz, 0.67H), 5.15 (d, J = 3.5 Hz, 0.33H), 4.33 (dt, J = 4.1, 2.3 Hz, 0.33H), 4.08 (dt, J = 4.1, 2.3 Hz, 0.67H), 3.76 – 3.19 (m, 3H), 3.05 – 2.86 (m, 3H), 2.71 (d, J = 10.8 Hz, 0.67H), 2.44 (d, J = 7.2 Hz, 0.33H), 2.23 – 1.94 (m, 4H), 1.90 – 1.79 (m, 1H), 1.73 – 1.49 (m, 3H), 1.26 – 1.19 (m, 1H), 1.18 (d, J = 7.1 Hz, 3 × 0.33H), 1.11 (d, J = 6.8 Hz, 3 × 0.67H).

^{13}C NMR (101 MHz, $CDCl_3$) δ 118.4, 118.3, 106.2, 100.5, 74.4, 73.5, 61.4, 60.2, 58.3, 55.1, 53.9, 53.9, 53.2 (2C), 45.0, 40.2, 37.5, 35.8, 27.4, 27.2, 27.1, 27.0, 19.2, 19.0, 18.5, 12.9.

IR (thin film): 2927, 1454, 1357, 1216, 1046, 969, 783 cm^{-1}.

HRMS (APCI+): *m/z* Calc. for $C_{13}H_{22}O_3N$ $[M+H]^+$: 240.1594, found: 240.1590.

TLC: R_f = 0.17 (CH_2Cl_2: MeOH = 10:1, Dragendorff's reagent, not UV active).

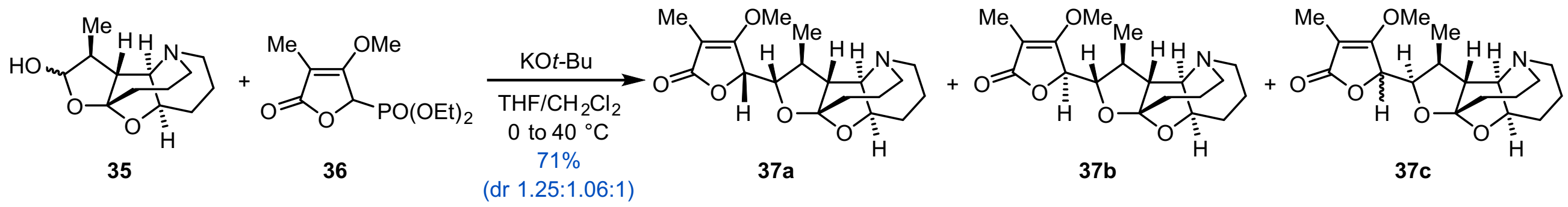

Under argon, potassium *tert*-butoxide (5.0 equiv, 141.0 mg, 1.25 mmol) was added at 0 °C to a round-bottom flask charged with a solution of **36** (5.0 equiv, 331.2 mg, 1.25 mmol, synthesized according to literature-reported procedures[7,8]) in THF (6 mL). After 1.5 h, a solution of **35** (1.0 equiv, 60.1 mg, 0.25 mmol) in THF (1.2 mL) and CH_2Cl_2 (2.4 mL) was added to the resulting red suspension, and the mixture was heated to 40 °C. After 4 h, the reaction was quenched with *sat.* NH_4Cl aq. (12 mL). The aqueous layer was extracted with CH_2Cl_2 (20 mL × 3), and the combined organic layers were dried over sodium sulfate, filtered, and concentrated *in vacuo*. The residue was purified by silica gel chromatography (CH_2Cl_2/MeOH = 40:1 → 20:1) to afford furanone **37** as a mixture of three diastereomers as a pale-yellow solid (62.4 mg, 71%, dr 1.25:1.06:1). The second major diastereomer of **37**, namely **37b**, was obtained as white needles by preparative TLC (CH_2Cl_2/MeOH = 10:1). The other two diastereomers, **37a** and **37c**, were obtained as an inseparable mixture.

Compound 37b (CCDC 2552359)**:**

^{1}H NMR (600 MHz, $CDCl_3$) δ 4.83 (dq, *J* = 3.6, 1.2 Hz, 1H), 4.17 (dt, *J* = 4.3, 2.2 Hz, 1H), 4.09 (s, 3H), 3.94 (dd, *J* = 8.8, 3.5 Hz, 1H), 3.36 – 3.30 (m, 1H), 3.28 (d, *J* = 2.1 Hz, 1H), 2.99 (ddt, *J* = 14.2, 4.1, 2.1 Hz, 1H), 2.94 – 2.87 (m, 2H), 2.49 (d, *J* = 9.5 Hz, 1H), 2.22 (dt, *J* = 17.7, 2.5 Hz, 1H), 2.18 – 2.12 (m, 2H), 2.03 – 1.94 (m, 1H), 1.97 (d, *J* = 1.3 Hz, 3H), 1.84 (qt, *J* = 13.2, 4.3 Hz, 1H), 1.69 (dddd, *J* = 15.0, 13.1, 5.6, 3.8 Hz, 1H), 1.61 – 1.55 (m, 1H), 1.52 (ddd, *J* = 13.3, 12.0, 5.5 Hz, 1H), 1.24 – 1.19 (m, 1H), 1.07 (d, *J* = 6.6 Hz, 3H).

^{13}C NMR (151 MHz, $CDCl_3$) δ 174.8, 171.6, 118.2, 99.1, 85.8, 77.8, 73.7, 61.1, 59.1, 58.6, 53.8, 53.3, 36.3, 35.9, 27.2 (2C), 19.1, 18.2, 8.6.

IR (thin film): 2921, 2874, 1744, 1666, 1453, 1392, 1334, 1315, 1231, 1106, 1078, 1047, 1000, 990, 974, 920 cm^{-1}.

HRMS (APCI+): *m/z* Calc. for $C_{19}H_{28}O_5N$ $[M+H]^+$: 350.1962, found: 350.1964.

TLC: R_f = 0.34 (CH_2Cl_2: MeOH = 10:1, Dragendorff's reagent, UV active).

Compound 37a and 37c (inseparable mixture of diastereomers, dr 1.25:1)**:**

^{1}H NMR (600 MHz, $CDCl_3$) δ 4.56 – 4.53 (m, 0.56H), 4.53 – 4.49 (m, 2 × 0.44H), 4.30 – 4.24 (m, 0.56H),

4.10 (s, 3 × 0.56H), 4.09 (s, 3 × 0.44H), 3.89 (dd, J = 9.2, 1.9 Hz, 0.56H), 3.41 – 3.32 (m, 1H), 3.29 (t, J = 1.6 Hz, 1H), 2.97 (dq, J = 14.2, 2.3 Hz, 1H), 2.94 – 2.85 (m, 2H), 2.71 (d, J = 10.7 Hz, 0.44H), 2.63 – 2.54 (m, 0.44H), 2.51 (d, J = 9.8 Hz, 0.56H), 2.39 (tq, J = 9.6, 6.5 Hz, 0.56H), 2.23 – 2.16 (m, 1H), 2.15 – 2.01 (m, 0.56H), 2.00 (d, J = 1.1 Hz, 3 × 0.44H), 1.98 (d, J = 1.3 Hz, 3 × 0.56H), 1.97 – 1.88 (m, 2 × 0.44 + 0.56H), 1.88 – 1.78 (m, 1H), 1.72 – 1.62 (m, 1H), 1.58 – 1.52 (m, 1H), 1.52 – 1.43 (m, 1H), 1.23 (d, J = 6.9 Hz, 3 × 0.44H), 1.22 – 1.17 (m, 1H), 1.14 (d, J = 6.6 Hz, 3 × 0.56H).

^{13}C NMR (151 MHz, CDCl$_3$) δ 175.4, 174.6, 171.3, 170.8, 119.2, 117.8, 98.6, 98.3, 83.6, 79.7, 77.5, 76.2, 73.36, 73.35, 61.0, 60.7, 59.0, 58.9, 58.3, 56.4, 53.7 (2C), 53.2, 53.1, 37.5, 37.1, 35.9, 34.8, 27.1, 27.0, 26.92, 26.90, 19.1, 18.9, 17.0, 14.4, 8.63, 8.57.

IR (thin film): 2927, 1750, 1668, 1455, 1390, 1340, 1311, 1230, 1153, 1076, 1048, 998, 988, 972, 920 cm^{-1}.

HRMS (APCI+): *m/z* Calc. for $C_{19}H_{28}O_5N$ $[M+H]^+$: 350.1962, found: 350.1963.

TLC: R_f = 0.32 (CH_2Cl_2: MeOH = 10:1, Dragendorff's reagent, UV active).

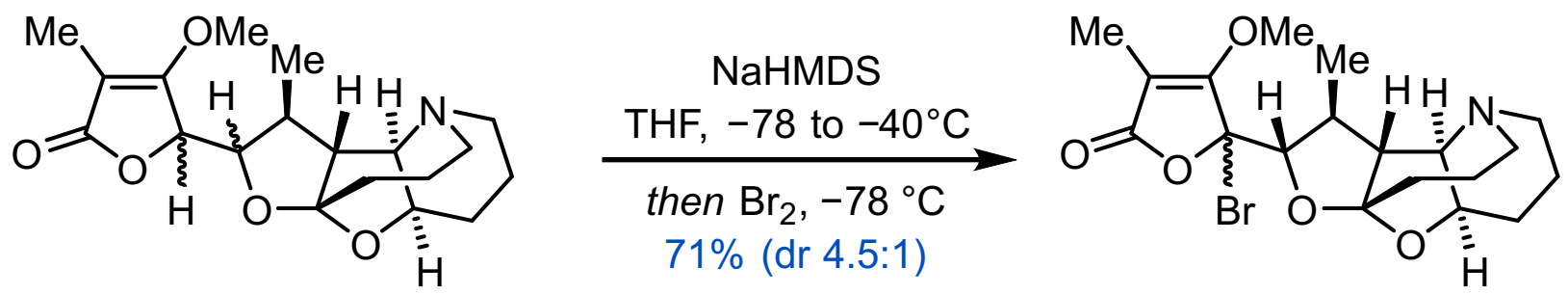

37 **38**

Under argon, sodium bis(trimethylsilyl)amide (2.0 equiv, 1.0 M in THF, 0.52 mL, 0.520 mmol) was added at −78 °C to a round-bottom flask charged with a solution of **37** (1.0 equiv, mixture of three diastereomers, 90.9 mg, 0.260 mmol) in THF (25 mL). The flask was then allowed to warm to −40 °C. After 1 h, the mixture was recooled to −78 °C, and a solution of bromine (2.0 equiv, 26.8 μL, 0.520 mmol) in THF (1 mL) was added dropwise. The reaction mixture was stirred for 1 h and quenched with *sat.* $Na_2S_2O_3$ aq. (5 mL) and sat. NH_4Cl aq. (2.5 mL). The aqueous layer was extracted with CH_2Cl_2 (10 mL × 4), and the combined organic layers were dried over sodium sulfate, filtered, and concentrated *in vacuo*. The residue was purified by silica gel chromatography (CH_2Cl_2/MeOH = 40:1 → 20:1) to afford **38** as a mixture of two diastereomers as a yellow oil (79.2 mg, 71%, dr 4.5:1).

Compound 38 (inseparable mixture of diastereomers, dr 4.5:1)**:**

^{1}H NMR (400 MHz, CDCl$_3$) δ 4.51 (dt, J = 4.1, 2.3 Hz, 0.82H), 4.23 (d, J = 7.0 Hz, 0.82H), 4.23 – 4.21 (m, 0.18H), 4.19 (s, 3 × 0.18H), 4.18 (s, 3 × 0.82H), 4.14 – 4.10 (m, 0.18H), 4.02 (d, J = 8.7 Hz, 0.18H), 3.41 – 3.31

(m, 2H), 3.03 – 2.89 (m, 3H), 2.66 (dt, J = 8.6, 6.7 Hz, 1H), 2.57 (d, J = 8.8 Hz, 1H), 2.21 – 2.16 (m, 1H), 2.10 – 2.03 (m, 1H), 2.04 (s, 3 × 0.18H), 2.02 (s, 3 × 0.82H), 2.00 – 1.91 (m, 1H), 1.90 – 1.78 (m, 1H), 1.76 – 1.68 (m, 1H), 1.60 – 1.54 (m, 1H), 1.53 – 1.36 (m, 1H), 1.42 (d, J = 6.6 Hz, 3 × 0.82H), 1.28 – 1.24 (m, 1H), 1.18 (d, J = 6.5 Hz, 3 × 0.18H).

^{13}C NMR (101 MHz, $CDCl_3$) δ 172.0*, 171.6†, 170.6†, 170.5*, 118.7†, 117.9*, 98.68*, 98.65†, 90.6*, 90.3†, 89.1*, 87.9†, 73.6*, 73.5†, 61.9†, 61.3*, 59.7†, 59.6*, 59.4†, 59.2*, 53.9†, 53.8*, 53.44†, 53.37*, 38.8†, 38.1*, 36.0*, 35.9†, 26.9†,* (2C), 26.6†,* (2C), 21.9†, 18.9*, 18.8†,* (2C), 8.8*, 8.7†.

Note: † = major diastereomer; * = minor diastereomer

IR (thin film): 2931, 1779, 1668, 1454, 1390, 1331, 1224, 1048, 1035, 998, 990, 968, 953, 913, 825, 811, 733 cm^{-1}.

HRMS (APCI+): *m/z* Calc. for $C_{19}H_{27}O_5NBr$ $[M+H]^+$: 428.1067, found: 428.1055.

TLC: R_f = 0.37 (CH_2Cl_2: MeOH = 10:1, Dragendorff's reagent, UV active).

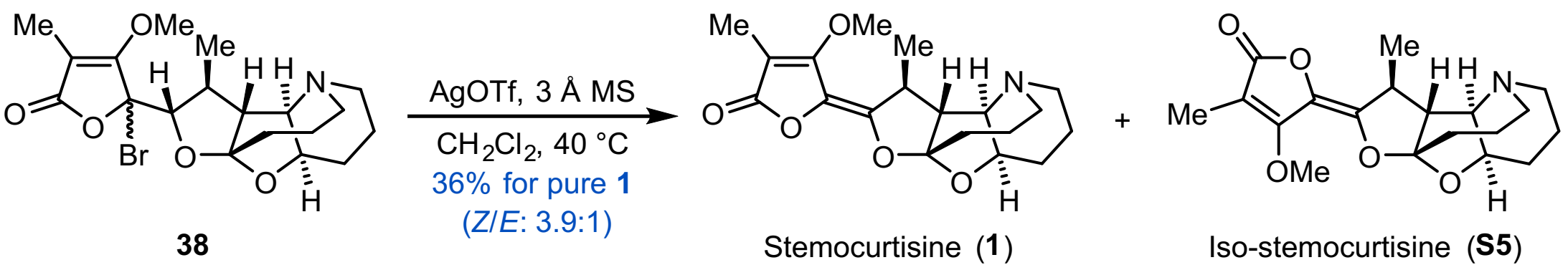

In a glovebox, a microwave vial was charged with **38** (1.0 equiv, 12.2 mg, 28.5 μmol), CH_2Cl_2 (1.4 mL), and 3 Å molecular sieves (1000 wt%, ~130 mg). The vial was sealed and stirred overnight. Silver trifluoromethanesulfonate (5.0 equiv, 36.6 mg, 0.142 mmol) was then added, and the mixture was heated to 40 °C.[9] After 1 h, the reaction was quenched with *sat.* $NaHCO_3$ aq. (1.5 mL). The aqueous layer was diluted with water (5 mL) and extracted with CH_2Cl_2 (10 mL × 3), and the combined organic layers were dried over sodium sulfate, filtered, and concentrated *in vacuo*. The residue was purified by preparative TLC (first purification: CH_2Cl_2/MeOH = 10:1; second purification: acetone/MeOH = 20:1, developed twice) to afford stemocurtisine (**1**) as a colorless oil (3.6 mg, 36%). Isostemocurtisine (**S5**) was also obtained; however, it overlapped with one of the hydrolysis byproducts. Attempts to separate the diastereomer by silica gel chromatography, preparative TLC, and different solvent systems were unsuccessful. The diastereomeric ratio of stemocurtisine to isostemocurtisine was determined by NMR after the first round of preparative TLC.

^{1}H NMR (600 MHz, $CDCl_3$) δ 4.15 (s, 3H), 4.03 – 3.98 (m, 1H), 3.43 (s, 1H), 3.38 (dd, J = 10.3, 8.7 Hz, 1H),

3.10 – 3.03 (m, 1H), 3.00 – 2.95 (m, 2H), 2.92 (td, J = 13.7, 2.8 Hz, 1H), 2.66 (d, J = 5.0 Hz, 1H), 2.41 – 2.34 (m, 1H), 2.21 (d, J = 15.1 Hz, 1H), 2.08 (s, 3H), 2.05 – 1.98 (m, 1H), 1.82 (qt, J = 13.3, 4.3 Hz, 1H), 1.74 (ddd, J = 13.2, 11.9, 5.6 Hz, 1H), 1.69 – 1.57 (m, 2H), 1.37 (d, J = 6.9 Hz, 3H), 1.21 (ddd, J = 13.4, 5.4, 2.5 Hz, 1H).

^{13}C NMR (151 MHz, $CDCl_3$) δ 170.1, 163.0, 147.5, 125.2, 120.7, 97.6, 75.6, 62.2, 59.1, 57.2, 53.7, 53.1, 39.4, 34.0, 27.1, 27.0, 22.7, 18.9, 9.3.

IR (thin film): 2928, 2872, 1740, 1691, 1619, 1458, 1397, 1366, 1283, 1223, 1133, 1062, 1039, 1021, 992, 983, 946, 912 cm^{-1}.

HRMS (APCI+): *m/z* Calc. for $C_{19}H_{26}O_5N$ $[M+H]^+$: 348.1806, found: 348.1816.

TLC: R_f = 0.40 (CH_2Cl_2: MeOH = 10:1, Dragendorff's reagent, UV active).

Asymmetric reduction of 20 to 21:

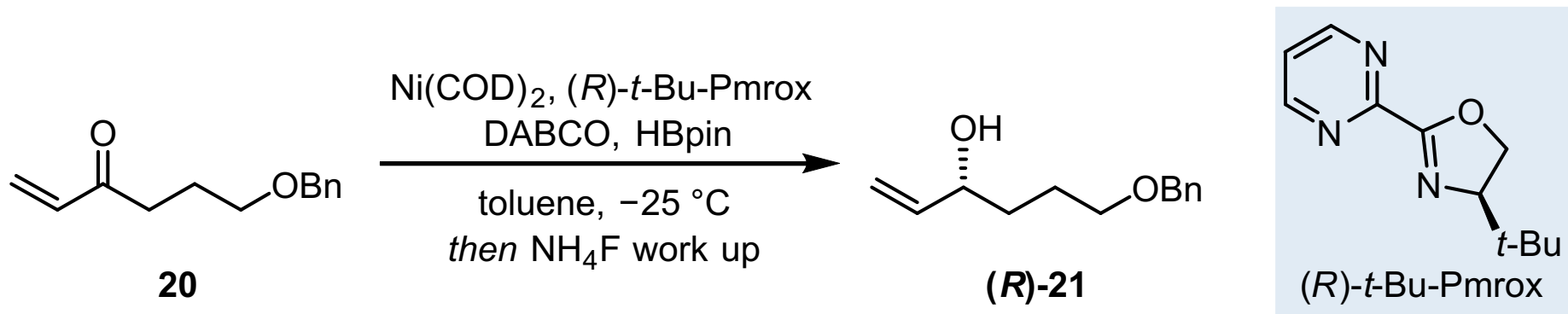

Nickel-catalyzed asymmetric reduction:[10] In a glovebox, a microwave vial was charged with $Ni(COD)_2$ (10 mol%, 5.5 mg, 0.02 mmol), (*R*)-*t*-Bu-Pmrox (12 mol%, 5.0 mg, 0.024 mmol), DABCO (1.5 equiv, 33.6 mg, 0.30 mmol) and toluene (0.8 mL). The vial was then sealed and stirred for 10 min. Pinacolborane (1.2 equiv, 35 μL, 0.24 mmol) was then added dropwise. The reaction mixture was cooled to −25 °C and stirred vigorously for 10 min. Then a solution of enone **20** (1.0 equiv, 40.6 mg, 0.20 mmol) in toluene (0.2 mL) was added dropwise to the mixture. After 1.5 h, the reaction was quenched with *sat.* NH_4F in MeOH (1 mL) and stirred for 15 min at room temperature. Water (1 mL) was then added, and the aqueous layer was extracted with EtOAc (3 mL × 3), and the combined organic layers were dried over sodium sulfate, filtered, and concentrated *in vacuo*. The residue was purified by silica gel chromatography (hexanes/EtOAc = 8:1 → 4:1) to afford (*R*)-**21** as a colorless oil (16.1 mg, 39%, 97% ee).

Note: The analytical data matches with the reported compound.[11]

Table S2. Condition optimizations for the asymmetric reduction of 20.

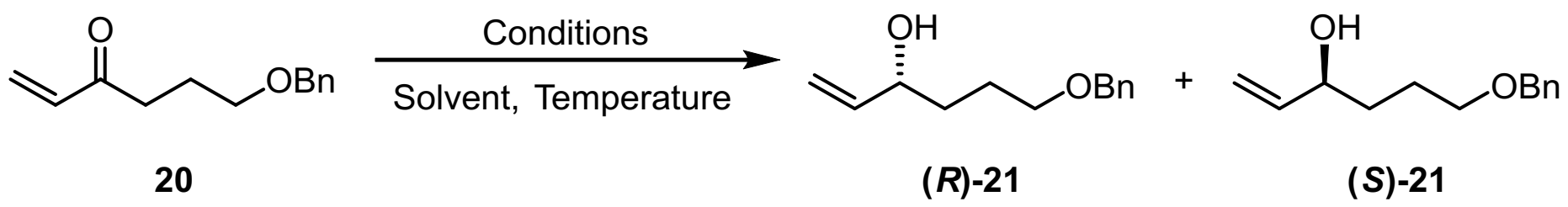

Entry	Condition	Yield	Enantiomeric Ratio (*R*:*S*)
1	(*S*)-Me-CBS, $BH_3 \cdot SMe_2$, THF, 0 °C	86%	85:15 (70% *ee*)
2	$Ni(COD)_2$ (10 mol%), (*S*)-*t*-Bu-Pmrox (12 mol%), DABCO, HBpin, toluene (wet), −25 °C	20%	5:95 (90% *ee*)
3	$Ni(COD)_2$ (10 mol%), (*S*)-*t*-Bu-Pmrox (12 mol%), DABCO, HBpin, toluene, −25 °C	45%	2:98 (96% *ee*)
4	$Ni(COD)_2$ (2 mol%), (*R*)-*t*-Bu-Pmrox (2.4 mol%), DABCO, HBpin, toluene, −25 °C	6%	90:10 (80% *ee*)
5	$Ni(COD)_2$ (5 mol%), (*R*)-*t*-Bu-Pmrox (6 mol%), DABCO, HBpin, toluene, −25 °C	20%	95.5:4.5 (91% *ee*)
6	$Ni(COD)_2$ (10 mol%), (*R*)-*t*-Bu-Pmrox (12 mol%), DABCO, HBpin, toluene, −25 °C	39%	98.5:1.5 (97% ee)

HPLC Chromatogram of *rac*-**21**:

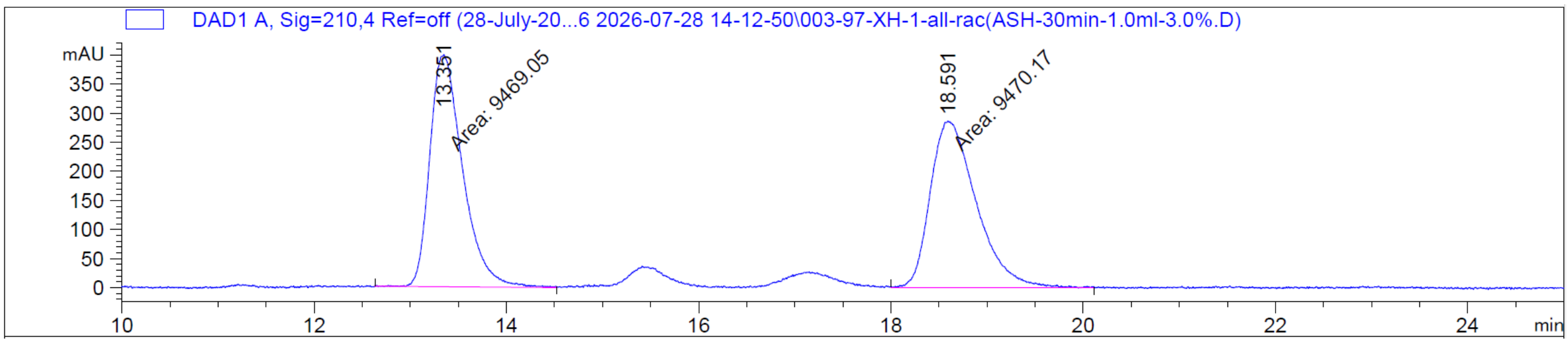

Peak #	RetTime [min]	Type	Width [min]	Area [mAU*s]	Height [mAU]	Area %
1	13.351	MM	0.3955	9469.04785	399.02026	49.9970
2	18.591	MM	0.5515	9470.16797	286.21149	50.0030

HPLC Chromatogram of (R)-**21** (97% *ee*; $[\alpha]_D^{23} = -0.9$ (c = 2.0, $CHCl_3$)):

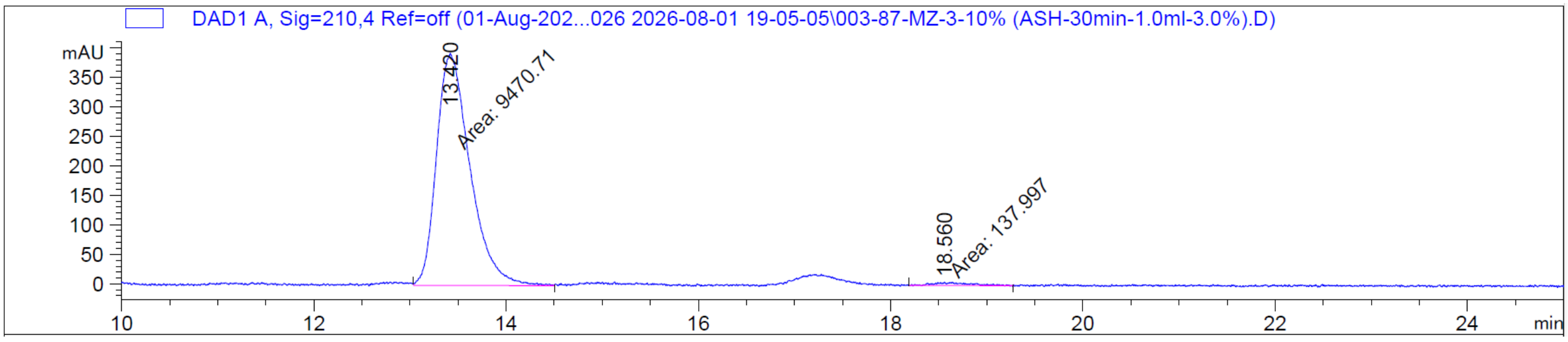

Signal 1: DAD1 A, Sig=210,4 Ref=off

Peak #	RetTime [min]	Type	Width [min]	Area [mAU*s]	Height [mAU]	Area %
1	13.420	MM	0.4015	9470.70508	393.13025	98.5638
2	18.560	MM	0.4059	137.99675	5.66577	1.4362

(R)-**21** (97% *ee*) was then converted to (R)-**23a** using the same procedure described above for the racemic synthesis to produce (R)-**23a** in 97% *ee*.

HPLC Chromatogram of *rac*-**23a**:

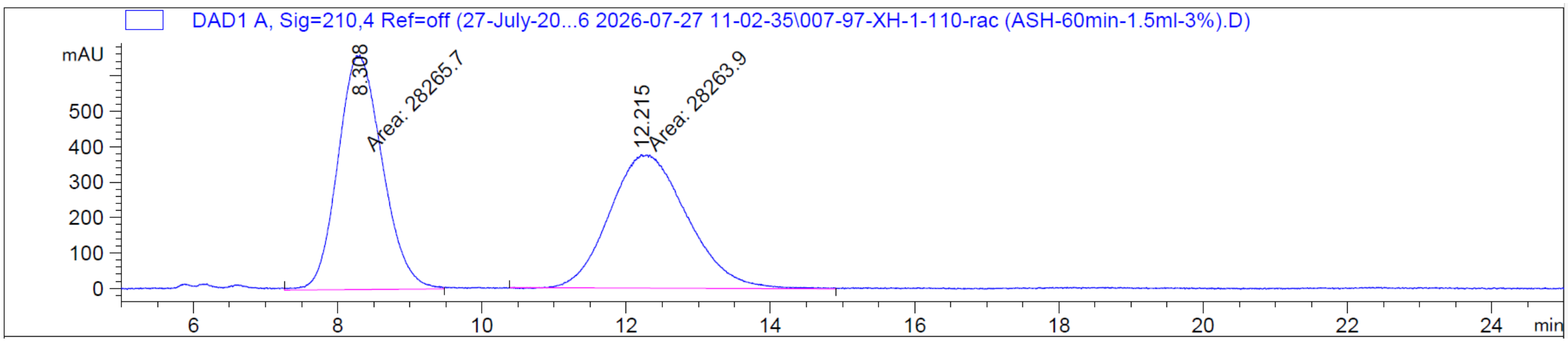

Signal 1: DAD1 A, Sig=210,4 Ref=off

Peak #	RetTime [min]	Type	Width [min]	Area [mAU*s]	Height [mAU]	Area %
1	8.308	MM	0.7119	2.82657e4	661.76208	50.0016
2	12.215	MM	1.2501	2.82639e4	376.80731	49.9984

HPLC Chromatogram of (*R*)-**23a** (97% *ee*; $[\alpha]_D^{23}$ = +17.9 (c = 2.0, $CHCl_3$)):

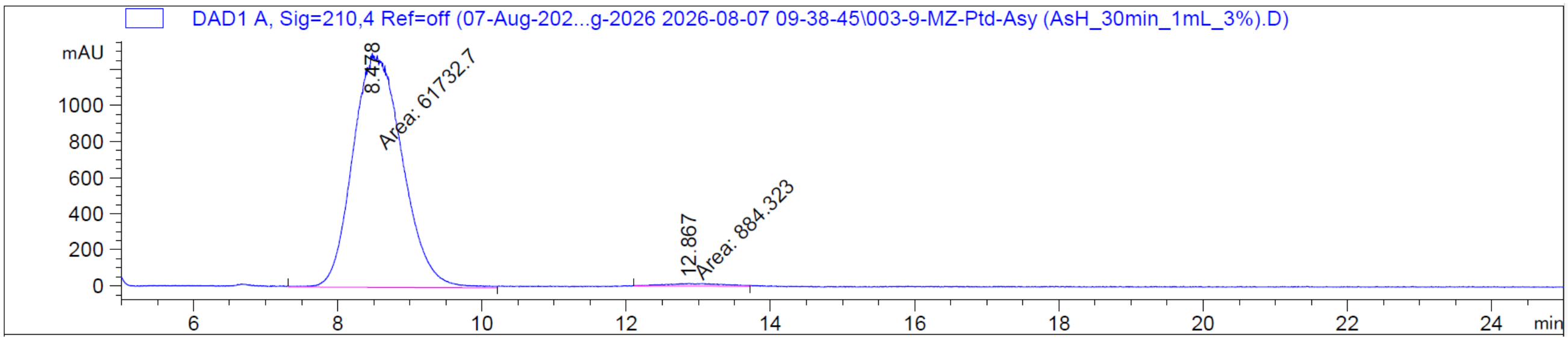

Signal 1: DAD1 A, Sig=210,4 Ref=off

Peak #	RetTime [min]	Type	Width [min]	Area [mAU*s]	Height [mAU]	Area %
1	8.478	MM	0.7918	6.17327e4	1299.38342	98.5877
2	12.867	MM	0.8957	884.32269	16.45506	1.4123

(*R*)-**23a** (97% *ee*) was then converted to **24a** using the same procedure described above for the racemic synthesis to produce **24a** in 97% *ee*.

HPLC Chromatogram of *rac*-**24a**:

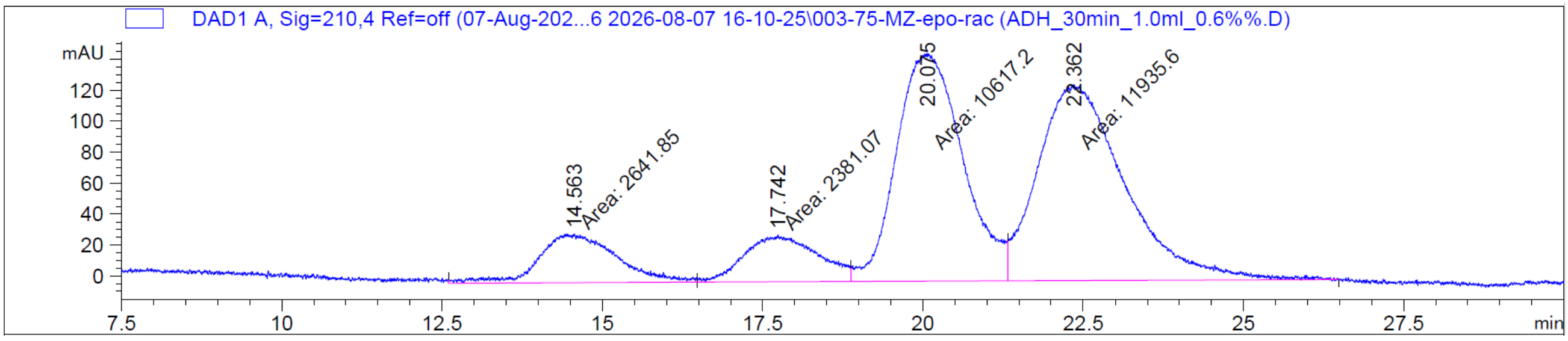

Peak #	RetTime [min]	Type	Width [min]	Area [mAU*s]	Height [mAU]	Area %
1	14.563	MF	1.3939	2641.85449	31.58861	9.5804
2	17.742	MF	1.3033	2381.06641	30.44935	8.6347
3	20.075	MF	1.2009	1.06172e4	147.34544	38.5021
4	22.362	FM	1.5689	1.19356e4	126.78970	43.2829

HPLC Chromatogram of enantioenriched **24a** (97% *ee*; $[\alpha]_D^{23}$ = +6.5 (c = 1.0, $CHCl_3$)):

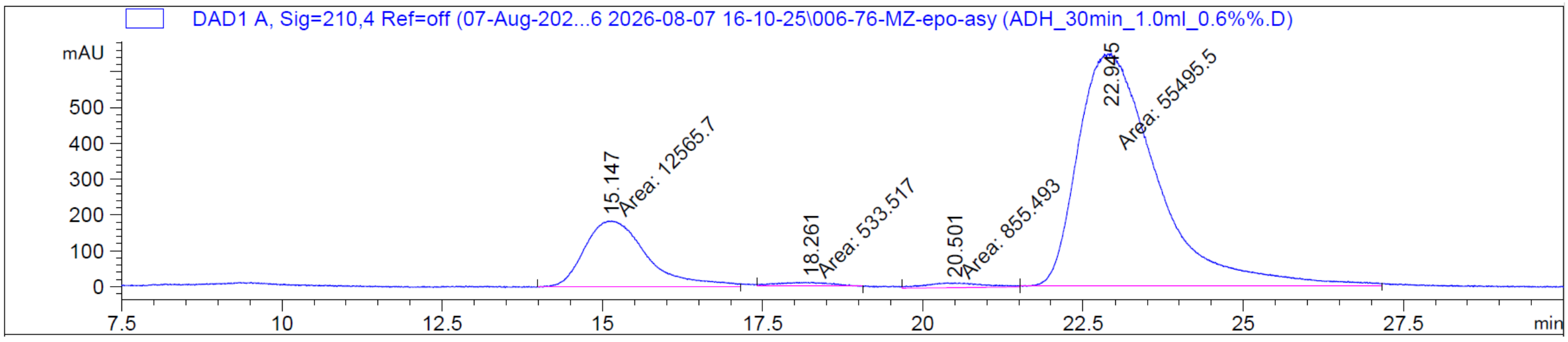

Signal 1: DAD1 A, Sig=210,4 Ref=off

Peak #	RetTime [min]	Type	Width [min]	Area [mAU*s]	Height [mAU]	Area %
1	15.147	MM	1.1340	1.25657e4	184.67622	18.0931
2	18.261	MM	0.9201	533.51678	9.66424	0.7682
3	20.501	MM	1.0534	855.49268	13.53528	1.2318
4	22.945	MM	1.4257	5.54955e4	648.73004	79.9069

C. NMR Comparison Tables.

Comparison of ^{1}H and ^{13}C NMR data for isolated[12] and synthetic Stemocurtisine

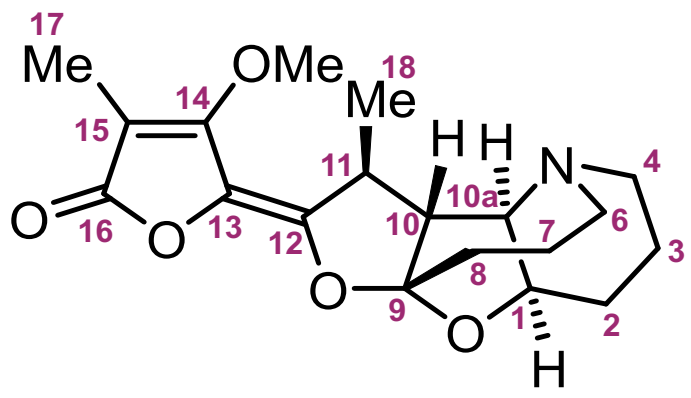

Stemocurtisine (**1**)

Position	^{1}H NMR (δ, ppm)		^{13}C NMR (δ, ppm)		
	Isolated, 500 MHz	Synthetic, 600 MHz	Isolated, 75 MHz	Synthetic, 151 MHz	Δδ
1	4.01 (s)	4.03 – 3.98 (m)	75.5	75.6	− 0.1
2	1.62 (m)	1.67 – 1.57 (m)*	26.9	27.0	− 0.1
	2.21 (d, *J* = 14.5 Hz)	2.21 (d, *J* = 15.1 Hz)			
3	1.21 (d, *J* = 13.5 Hz)	1.21 (ddd, *J* = 13.4, 5.4, 2.5 Hz)	18.9	18.9	0
	1.82 (m)	1.82 (qt, *J* = 13.3, 4.3 Hz)			
4	2.87 (m)	2.92 (td, *J* = 13.7, 2.8 Hz)	53.6	53.7	− 0.1
	3.02 (m)	3.02 – 2.97 (m)			
6	3.38 (t, *J* = 13 Hz)	3.38 (dd, *J* = 10.3, 8.7 Hz)	53.0	53.1	− 0.1
	2.96 (m)	3.00 – 2.95 (m)			
7	2.03 (m)	2.05 – 1.98 (m)	27.0	27.1	− 0.1
	1.66 (m)	1.69 – 1.63 (m)*			
8	2.36 (dd, *J* = 13.5, 4.5 Hz)	2.41 – 2.34 (m)	33.9	34.0	− 0.1
	1.75 (m)	1.74 (ddd, *J* = 13.2, 11.9, 5.6 Hz)			
9			120.4	120.7	− 0.3
10	2.65 (d, *J* = 4.5 Hz)	2.66 (d, *J* = 5.0 Hz)	57.0	57.2	− 0.2
10a	3.44 (s)	3.43 (s)	62.0	62.2	− 0.2
11	3.07 (quin, *J* = 6.5 Hz)	3.10 – 3.03 (m)	39.3	39.4	− 0.1
12			147.2	147.5	− 0.3
13			124.9	125.2	− 0.3
14			162.7	163.0	− 0.3
15			97.3	97.6	− 0.3
16			169.8	170.1	− 0.3
17	2.08 (s)	2.08 (s)	9.2	9.3	− 0.1
18	1.37 (d, *J* = 7 Hz)	1.37 (d, *J* = 6.9 Hz)	22.6	22.7	− 0.1
OMe	4.15 (s)	4.15 (s)	58.9	59.1	− 0.2

*Estimated from COSY correlations due to overlap with the residual water peak.

D. X-ray Crystal Data and Experimental.

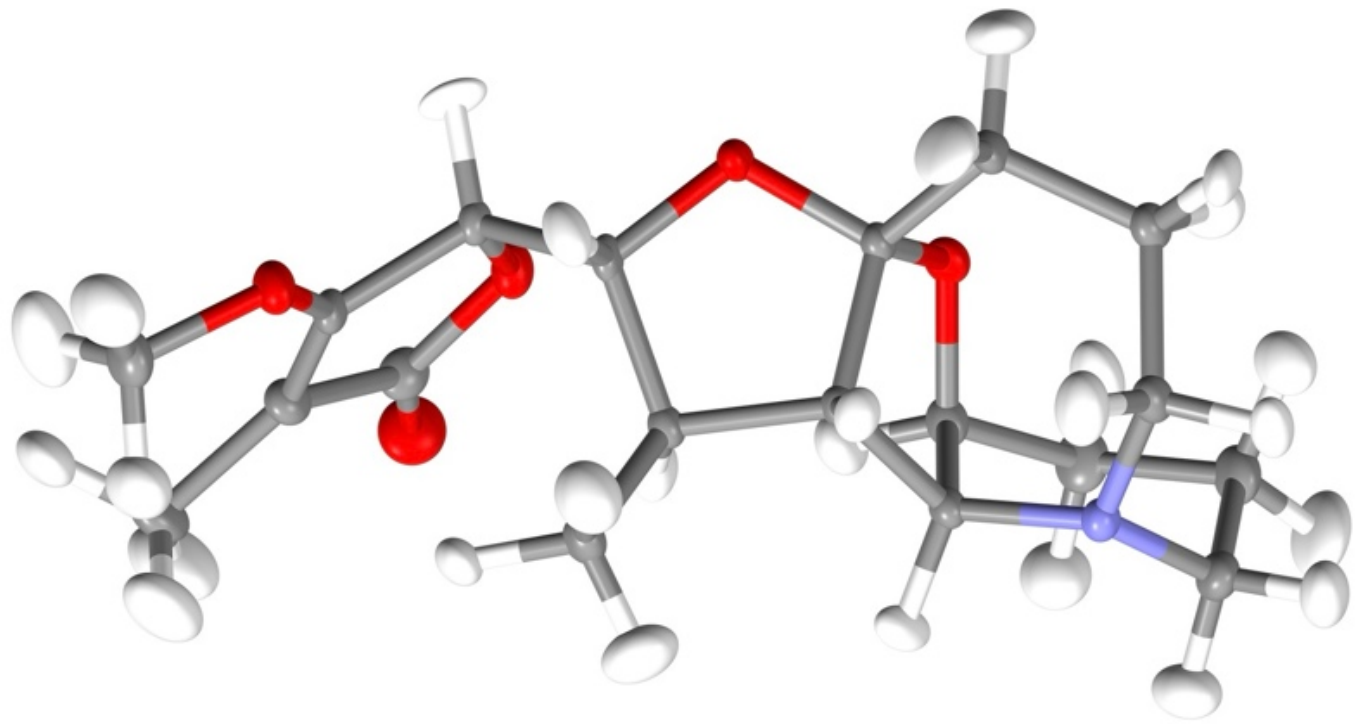

Figure S1. Crystal data and structure refinement for compound (±)-**37b** (CCDC 2552359).

Experimental. Single colorless plate-shaped crystals of MZ-II-230-1 were recrystallised from methanol by slow evaporation. A suitable crystal with dimensions 0.31 × 0.23 × 0.05 mm was selected and mounted on a nylon loop with NVH immersion oil on a XtaLAB Synergy, Dualflex, HyPix diffractometer. The crystal was kept at a constant T = 100.0(1) K during data collection. The structure was solved with ShelXT (Sheldrick, 2015) and Olex2 1.5-dev (Dolomanov et al., 2009). The crystal structure was refined with olex2.refine 1.5-dev (Bourhis et al., 2015) using full matrix least squares minimisation on $|\mathbf{F}|^2$.

Crystal Data. $C_{19}H_{27}NO_5$, M_r = 349.430, monoclinic, $P2_1$ (No. 4), a = 8.4523(1) Å, b = 7.6240(1) Å, c = 14.0436(2) Å, b = 105.894(1)°, a = g = 90°, V = 870.38(2) Å^3, T = 100.00(10) K, Z = 2, Z' = 1, m(Cu K_a) = 0.788, 18347 reflections measured, 3627 unique (R_{int} = 0.0330) which were used in all calculations. The final wR_2 was 0.0795 (all data) and R_1 was 0.0324 (I≥2s(I)).

E. References.

[1] Kerr, W. J.; Morrison, A. J.; Paterson, L. C. Synthesis of *α*-Methylene Propellanone via the Strategic Employment of Metal-Mediated Cyclisation Chemistry. *Tetrahedron* **2015**, *71*, 5356−5361.

[2] Labarre-Lainé, J.; Beniazza, R.; Desvergnes, V.; Landais, Y. Convergent Access to Bis-Spiroacetals through a Sila-Stetter–Ketalization Cascade. *Org. Lett.* **2013**, *15*, 4706−4709.

[3] Yamamoto, K.; Yamamoto, N. Epoxidation of *α*,*β*-Unsaturated Ketones Using Dialkylzinc–Oxygen Reagents. *Chem. Lett.* **1989**, *18*, 1149–1152.

[4] Nomura, K.; Oshima, K.; Matsubara, S. Stereospecific and Stereoselective Preparation of 2-(1-Hydroxyalkyl)-1-alkylcyclopropanols from *α*,*β*-Epoxy Ketones and Bis(iodozincio)methane. *Angew. Chem. Int. Ed.* **2005**, *44*, 5860–5863.

[5] Cai, X.; Liang, W.; Liu, M.; Li, X.; Dai, M. Catalytic Hydroxycyclopropanol Ring-Opening Carbonylative Lactonization to Fused Bicyclic Lactones. *J. Am. Chem. Soc.* **2020**, *142*, 13677–13682.

[6] Ng, X. Q.; Lim, C. S.; Liaw, M. W.; Quach, T. T.; Yang, B.-M.; Isoni, V.; Wu, J.; Zhao, Y. Direct Access to Chiral Aliphatic Amines by Catalytic Enantioconvergent Redox-Neutral Amination of Alcohols. *Nat. Synth*. **2023**, *2*, 572–580.

[7] Kayser, M. M.; Breau, L.; Eliev, S.; Morand, P.; Ip, H. S. Regioselectivity Control in Metal Hydride Reductions of Substituted Maleic Anhydrides. *Can. J. Chem.* **1986**, *64*, 104–109.

[8] Huang, X.-Z.; Gao, L.-H.; Huang, P.-Q. Enantioselective Total Syntheses of (+)-Stemofoline and Three Congeners Based on a Biogenetic Hypothesis. *Nat. Commun.* **2020**, *11*, 5314.

[9] Soda, Y.; Sugiyama, Y.; Yoritate, M.; Tajima, H.; Shibuya, K.; Ogihara, C.; Oishi, T.; Sato, T.; Chida, N. Unified Total Synthesis of Pentacyclic Stemoamide-Type Alkaloids. *Org. Lett.* **2020**, *22*, 7502-7507.

[10] Chen, F.; Zhang, Y.; Yu, L.; Zhu, S. Enantioselective NiH/Pmrox-Catalyzed 1,2-Reduction of *α*,*β*-Unsaturated Ketones. *Angew. Chem., Int. Ed.* **2017**, *56*, 2022–2025.

[11] Venkatesham, A.; Nagaiah, K. Stereoselective Total Synthesis of Stagonolide-C. *Tetrahedron: Asymmetry* **2012**, *23*, 1186–1197.

[12] Mungkornasawakul, P.; Pyne, S. G.; Jatisatienr, A.; Supyen, D.; Lie, W.; Ung, A. T.; Skelton, B. W.; White, A. H. Stemocurtisine, the First Pyrido[1,2-*a*]azepine *Stemona* Alkaloid. *J. Nat. Prod.* **2003**, *66*, 980–982.

F. ^1H, ^{13}C and 2D NMR Spectra

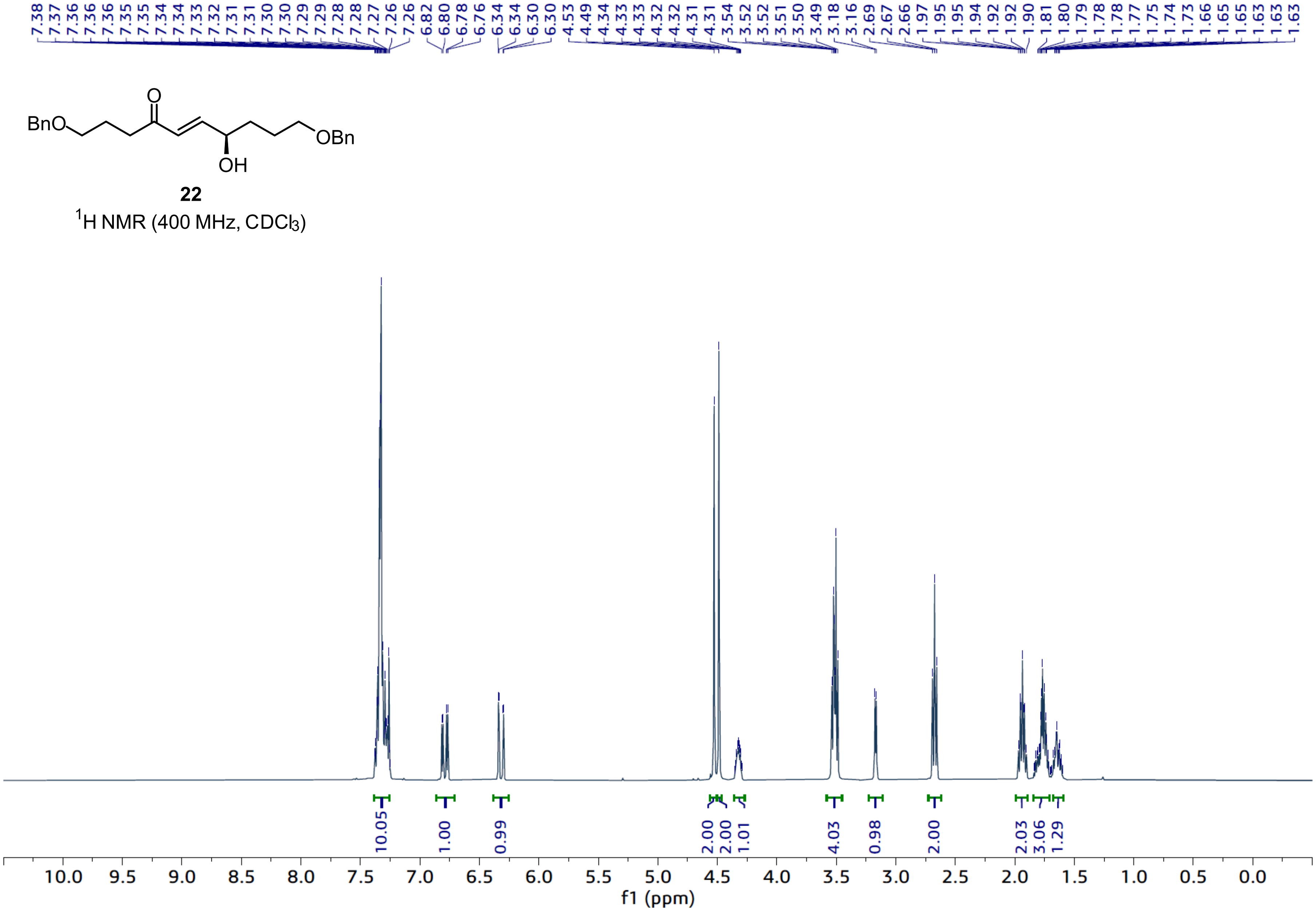
BnO
O
OH
OBn
22
1H NMR (400 MHz, CDCl3)
f1 (ppm)

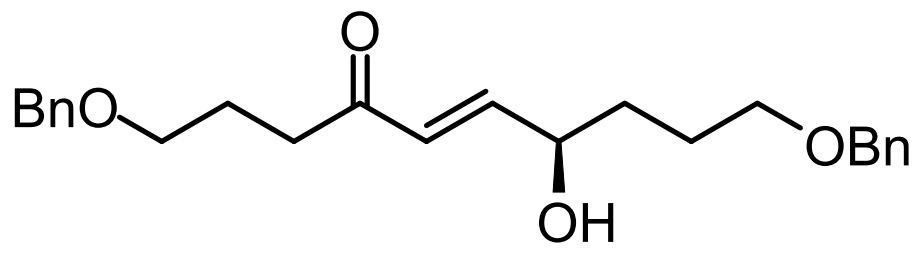

22

^{13}C NMR (101 MHz, $CDCl_3$)

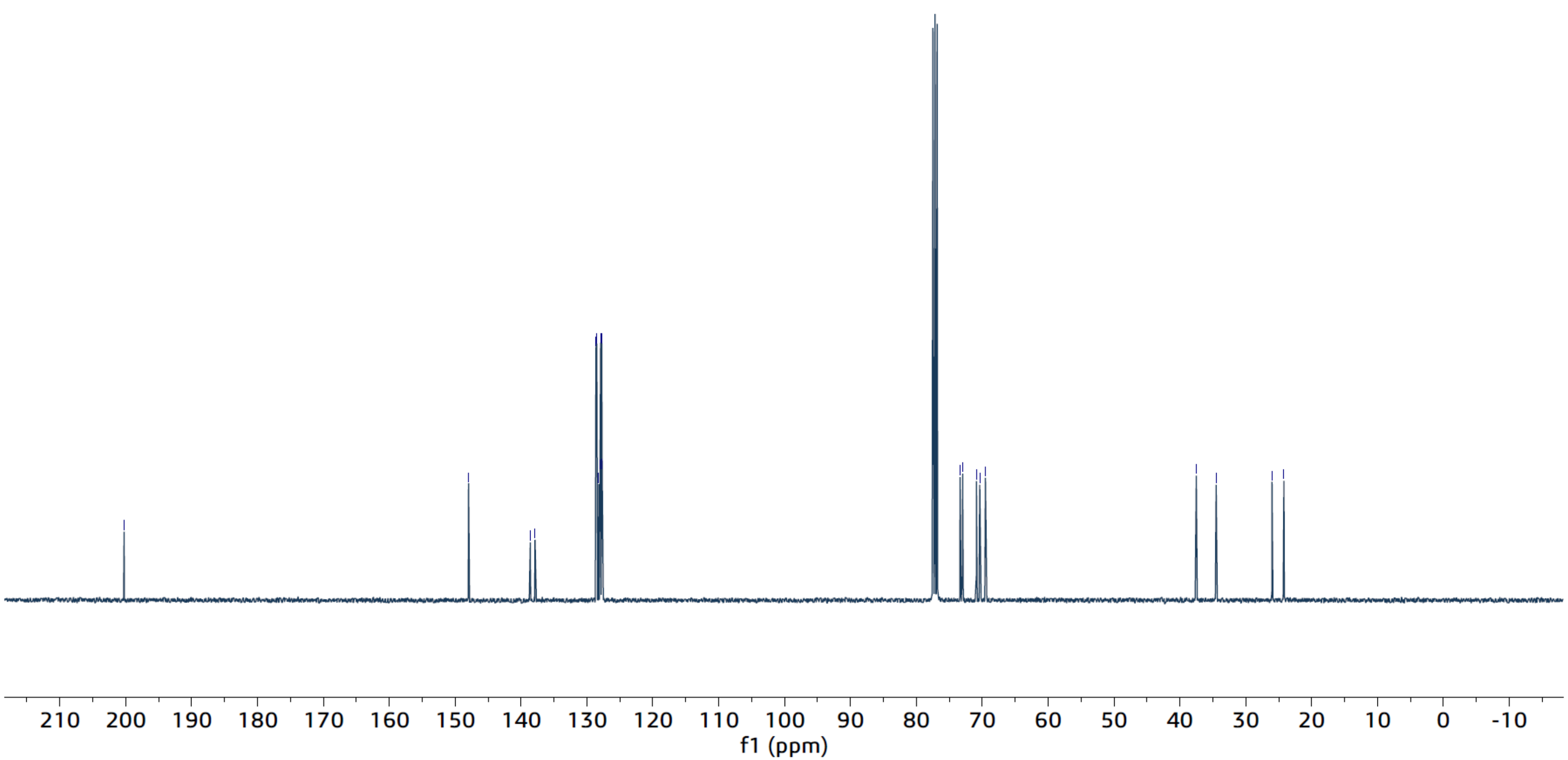

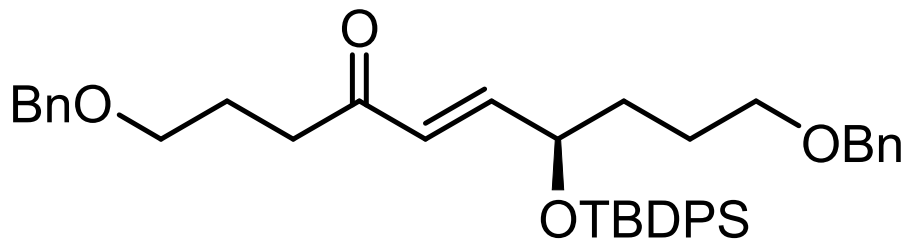

23a

^{1}H NMR (400 MHz, $CDCl_3$)

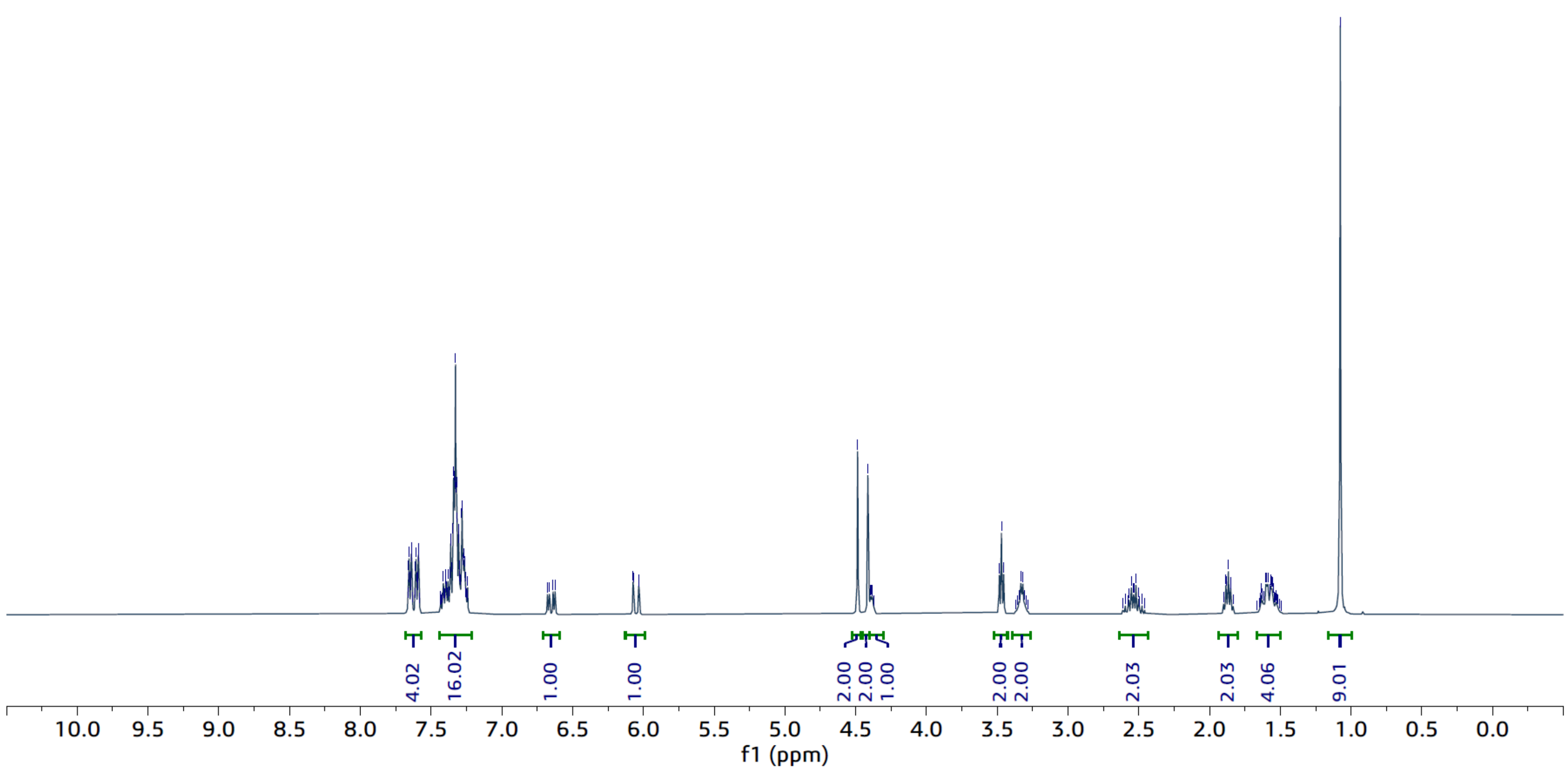

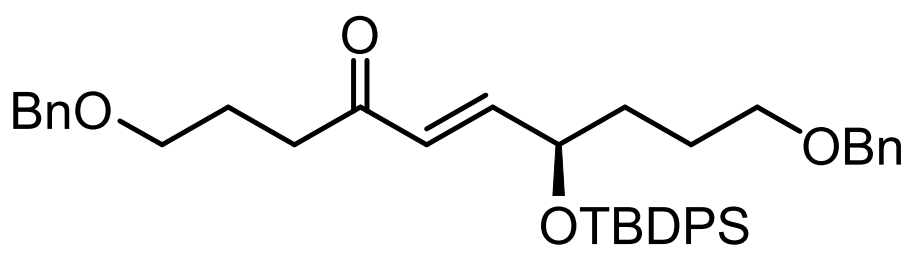

23a

^{13}C NMR (101 MHz, $CDCl_3$)

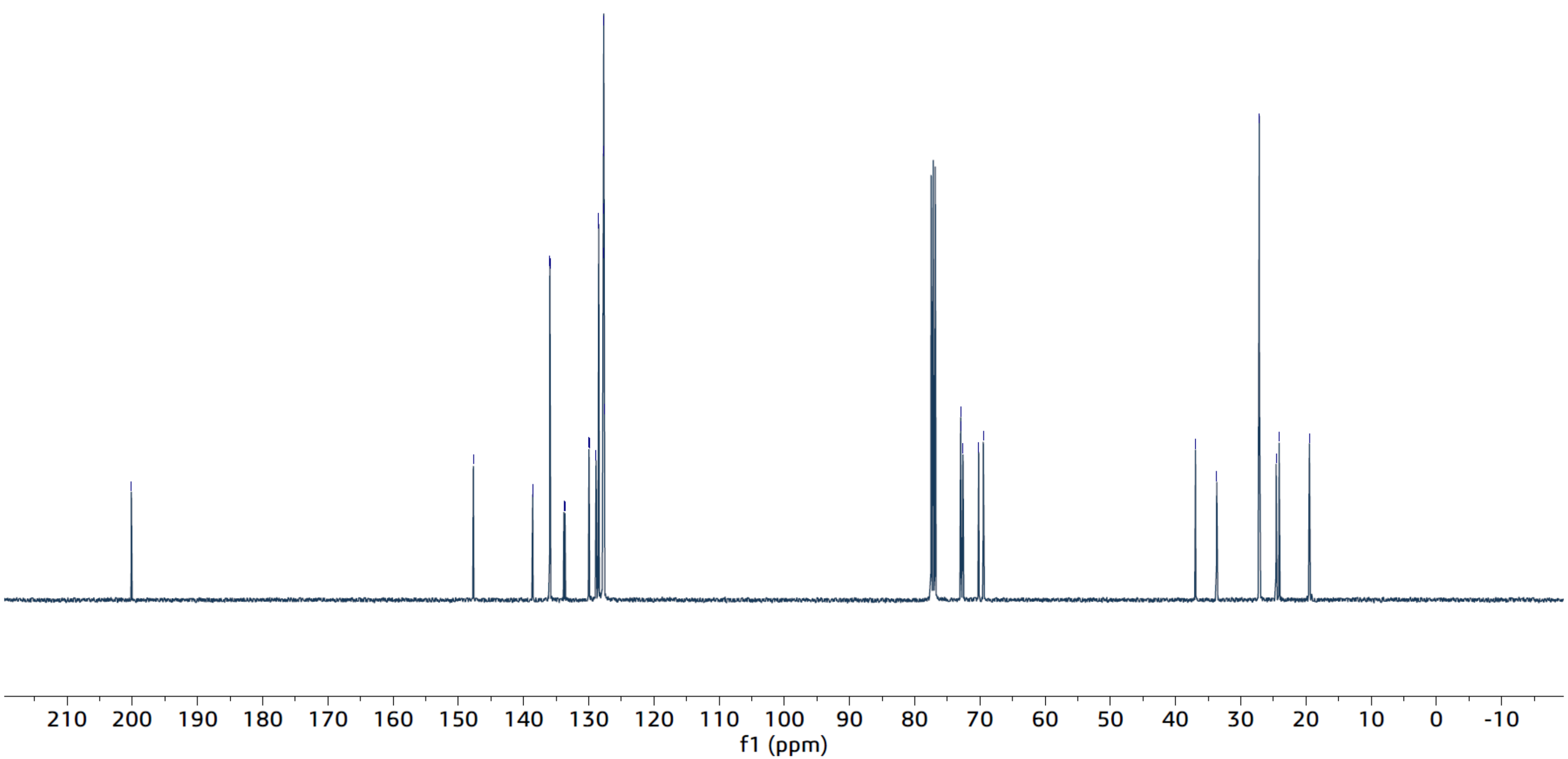

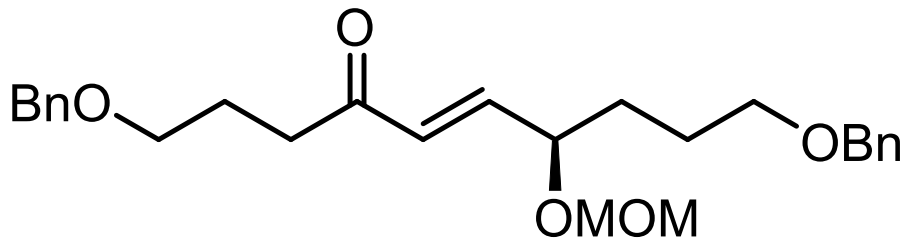

23b

^{1}H NMR (600 MHz, $CDCl_3$)

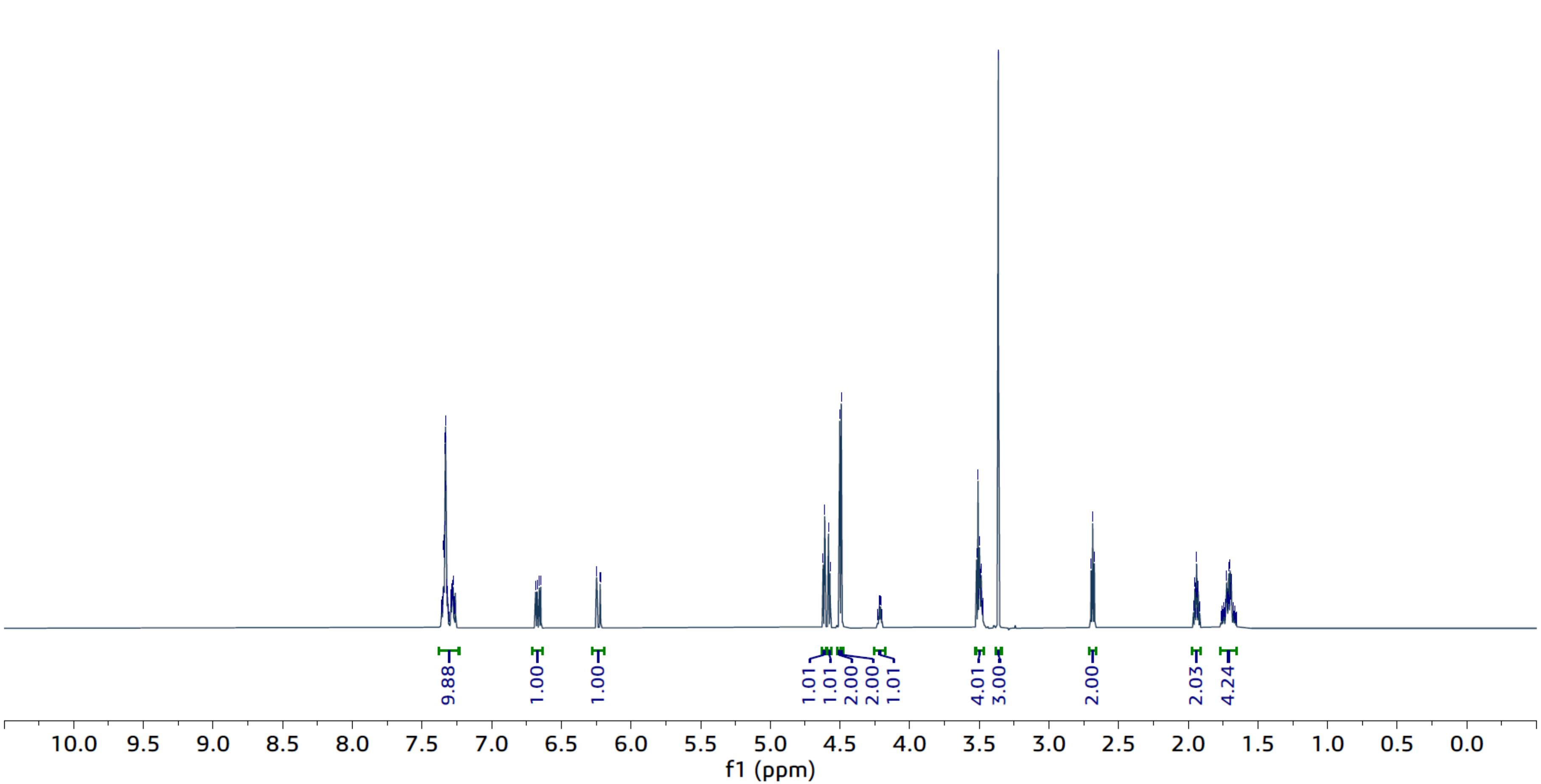

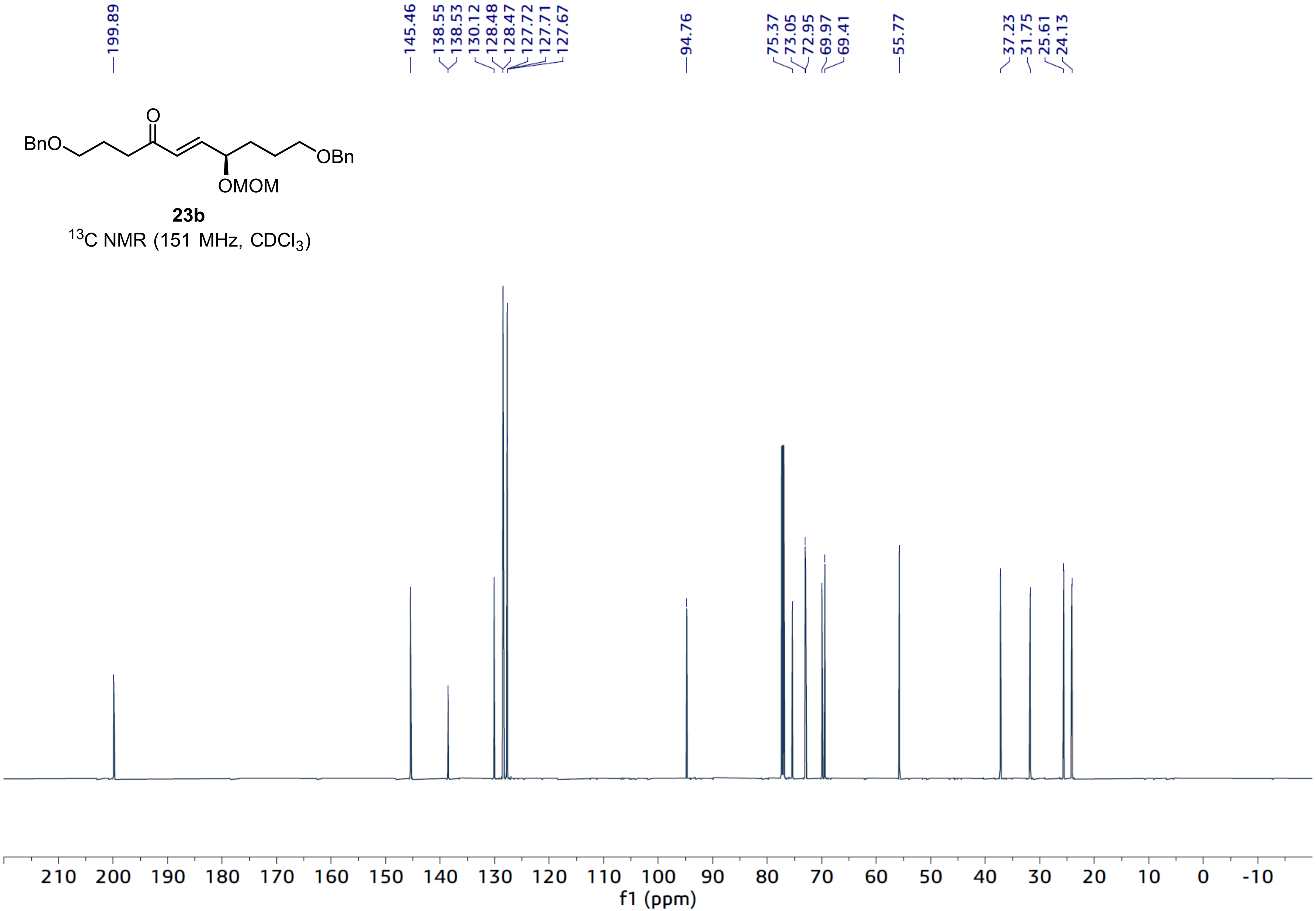

23b

^{13}C NMR (151 MHz, $CDCl_3$)

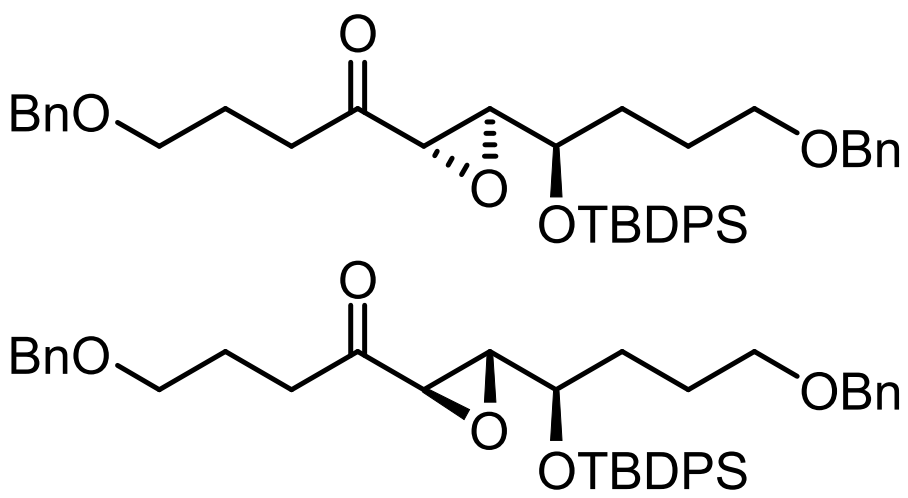

24a/S1 (dr 3.3:1)

^{1}H NMR (400 MHz, $CDCl_3$)

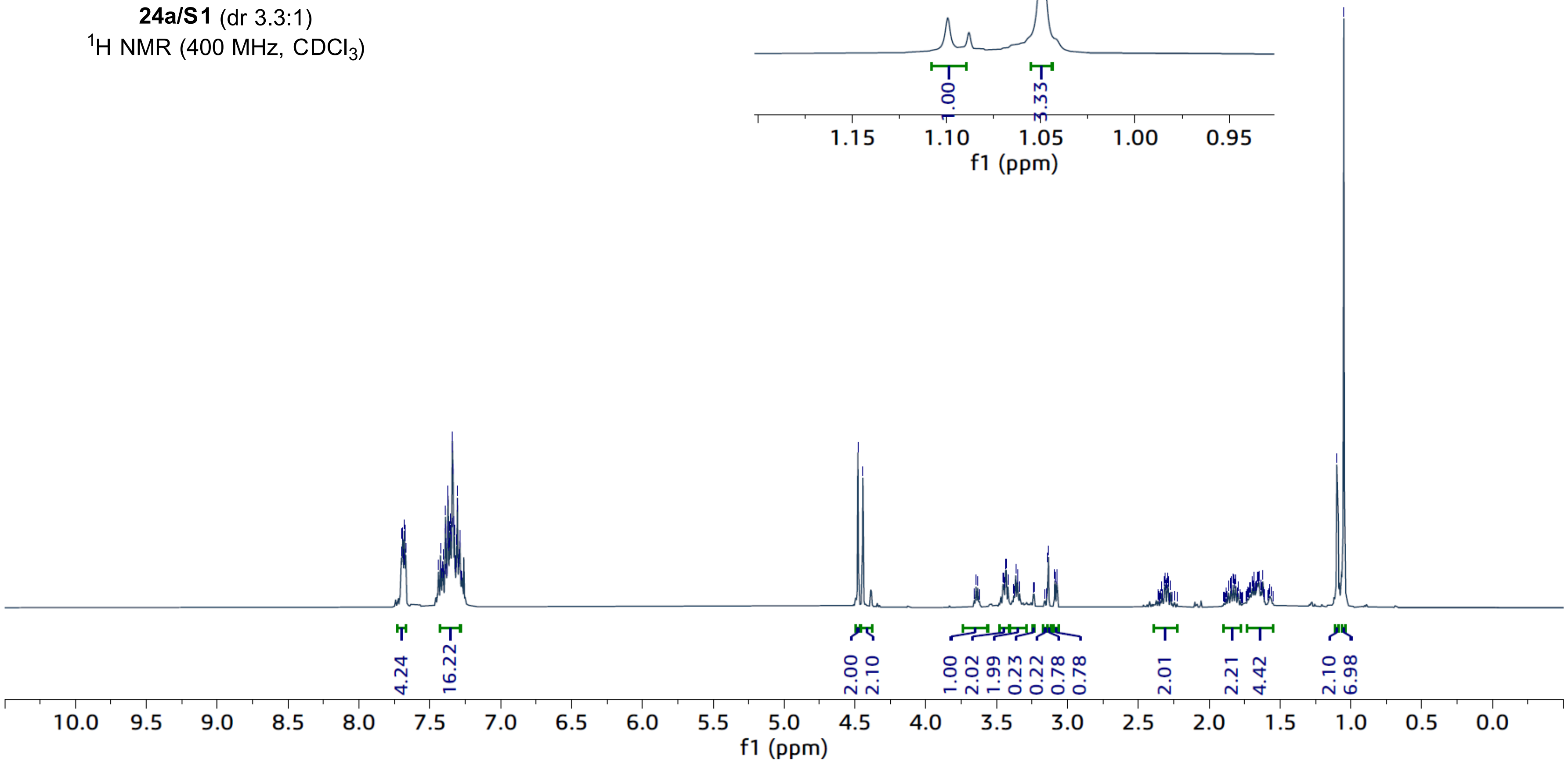

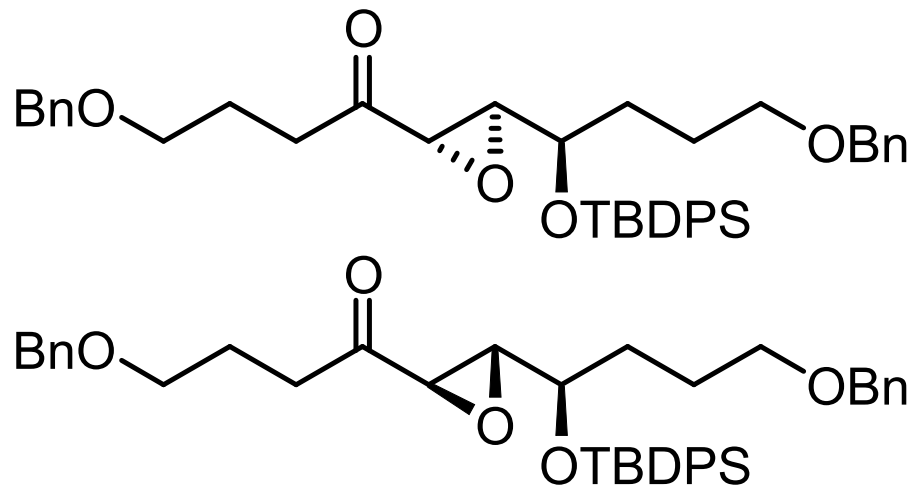

24a/S1 (dr 3.3:1)

^{13}C NMR (101 MHz, $CDCl_3$)

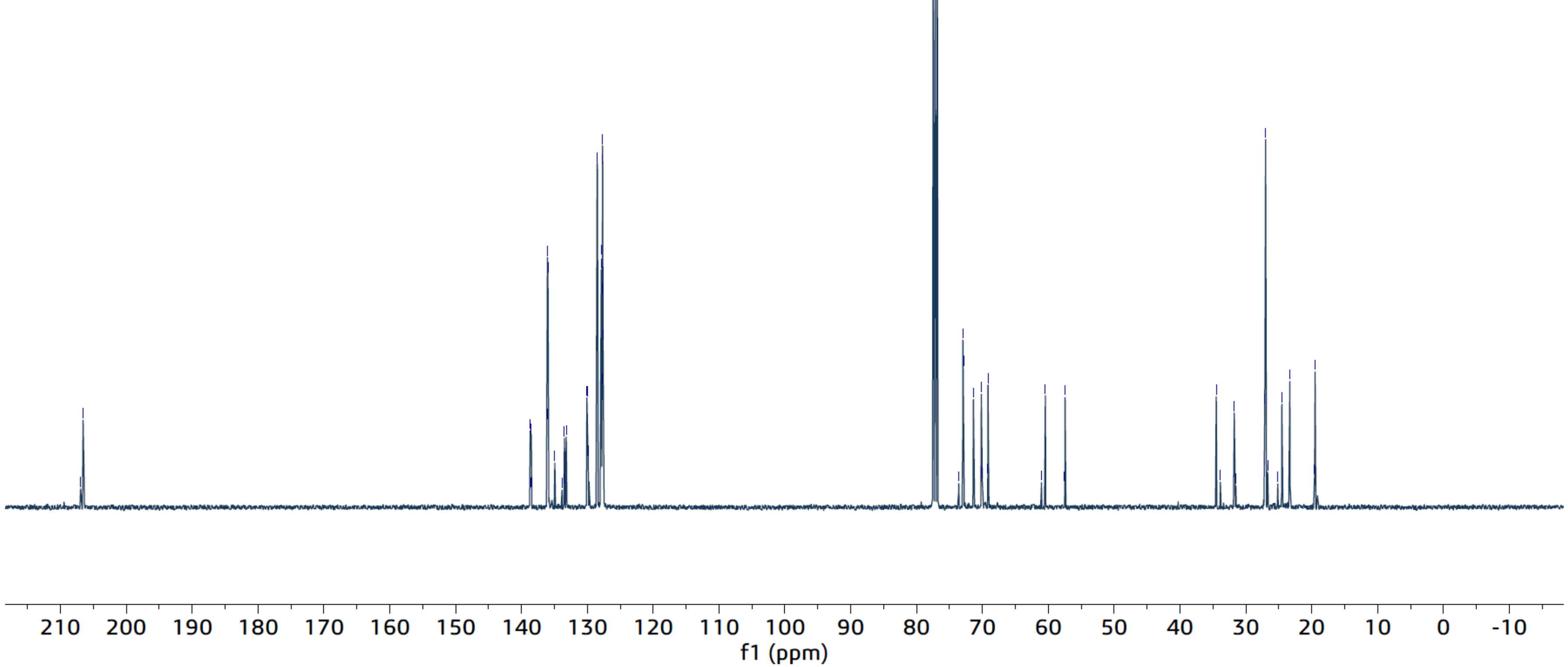

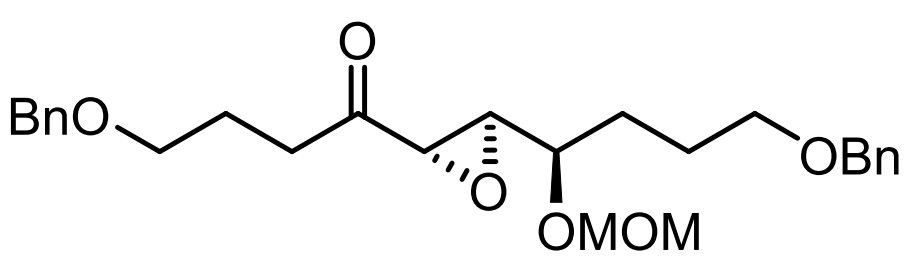

24b (dr > 20:1)

^{1}H NMR (400 MHz, $CDCl_3$)

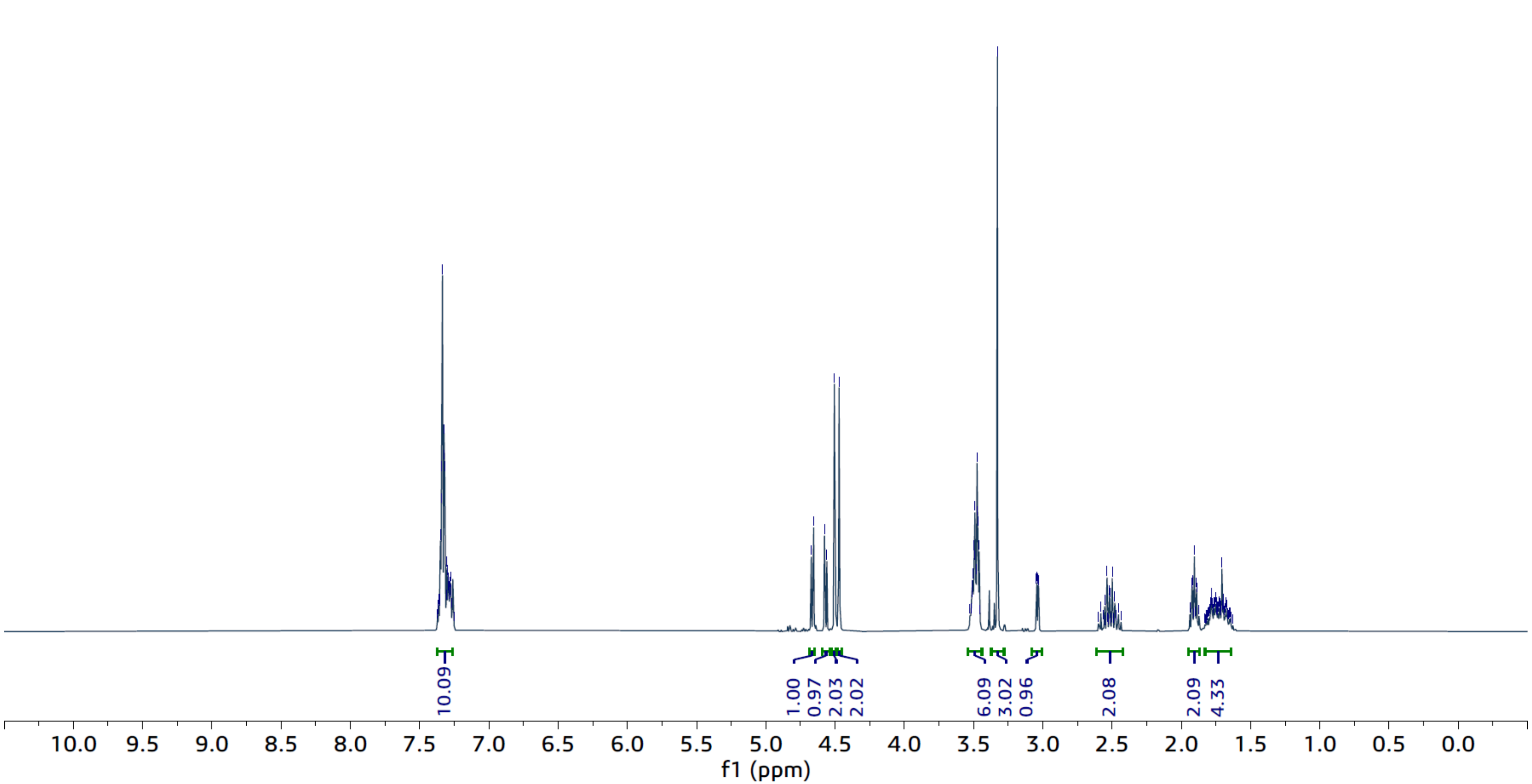

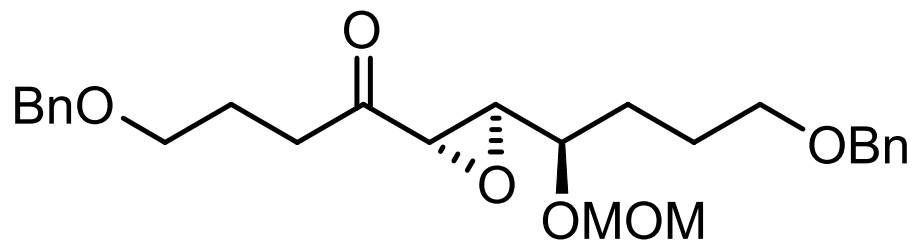

24b (dr > 20:1)
^{13}C NMR (101 MHz, $CDCl_3$)

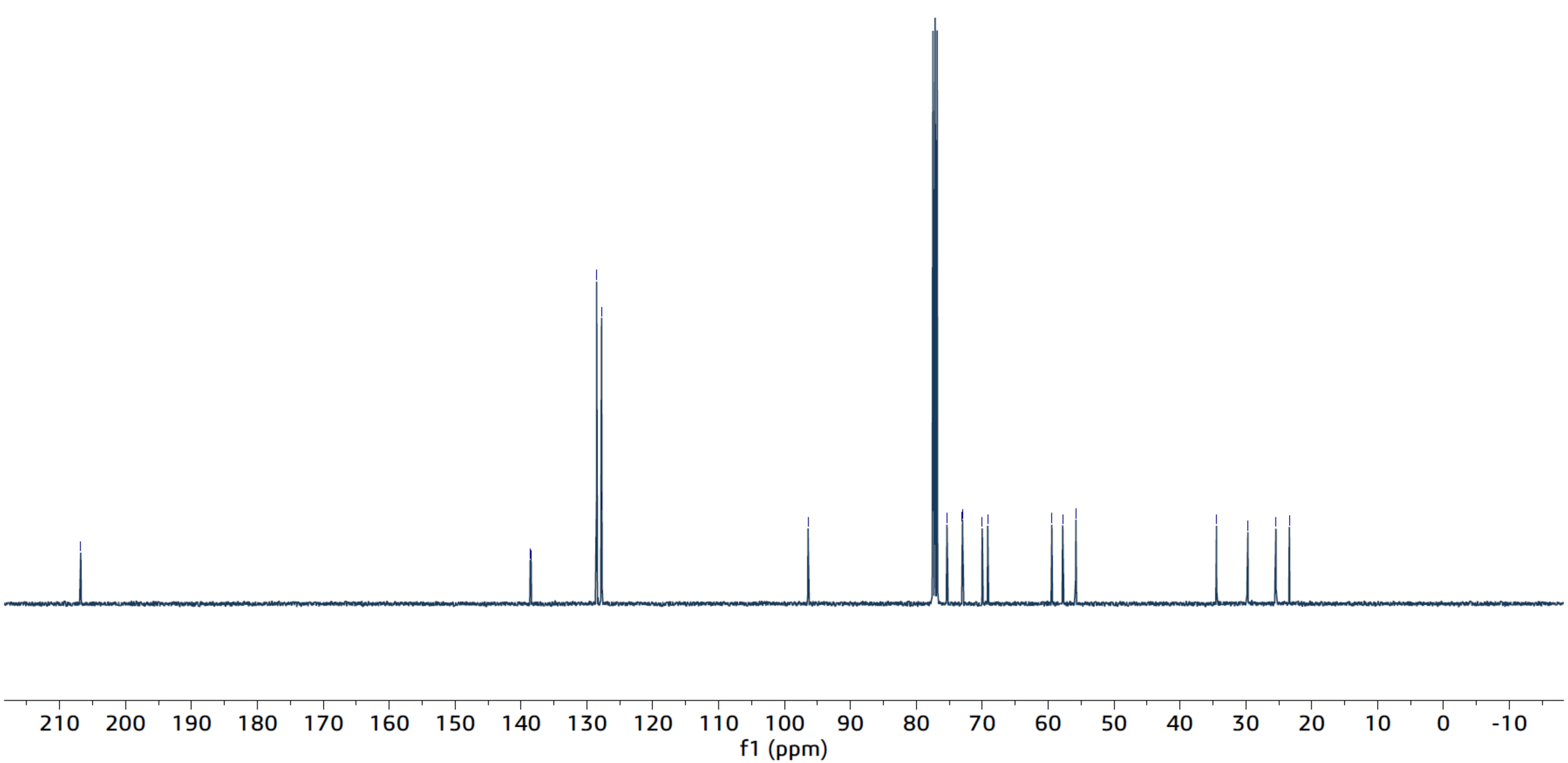

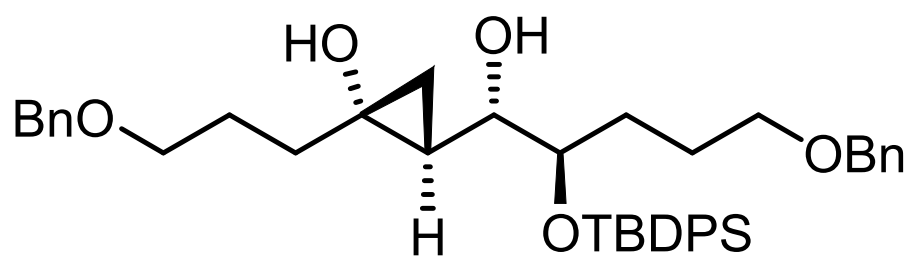

25a

^{1}H NMR (400 MHz, $CDCl_3$)

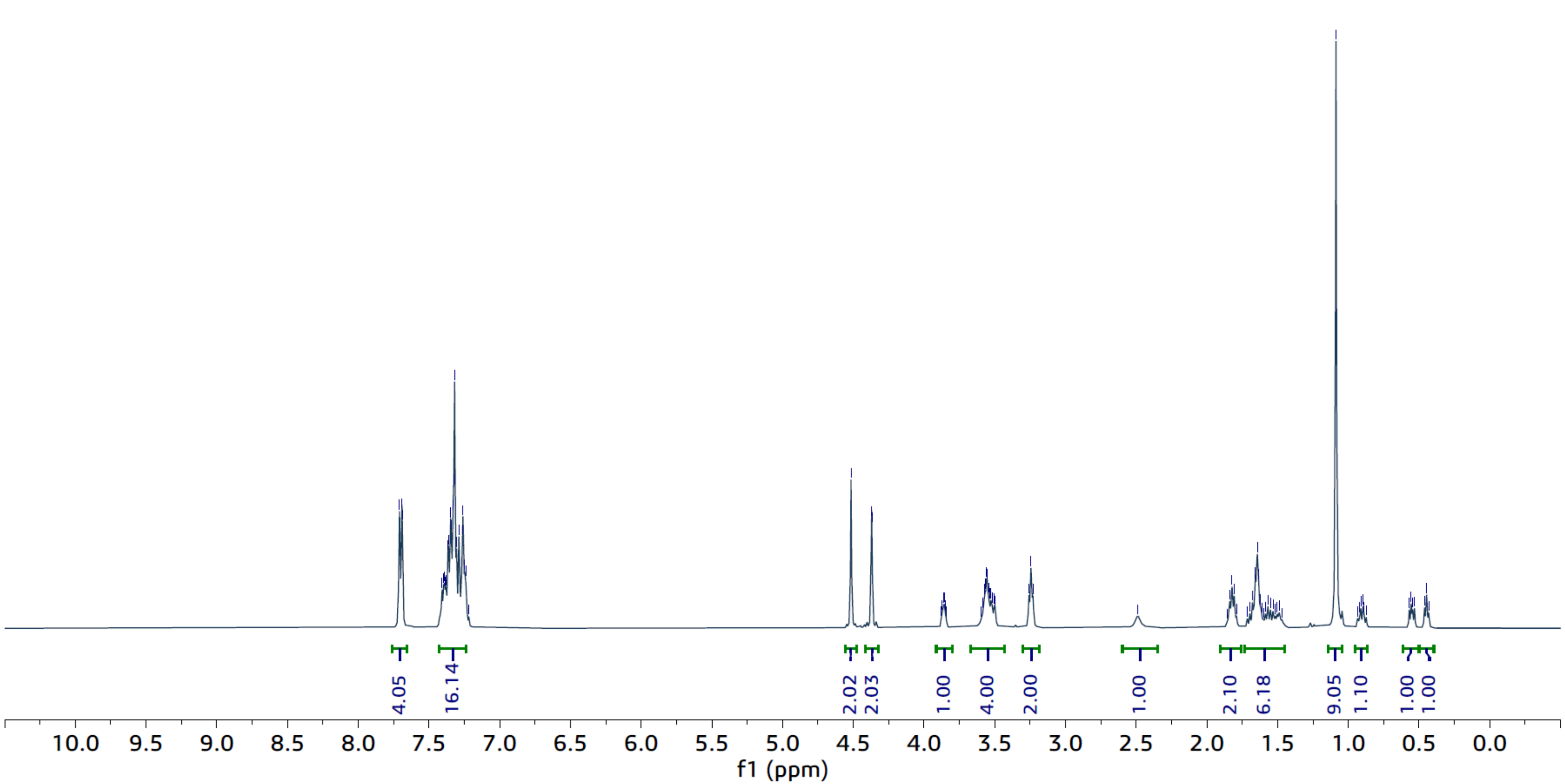

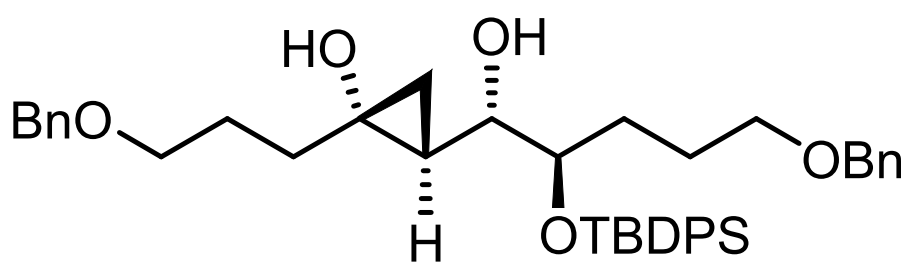

25a

^{13}C NMR (101 MHz, $CDCl_3$)

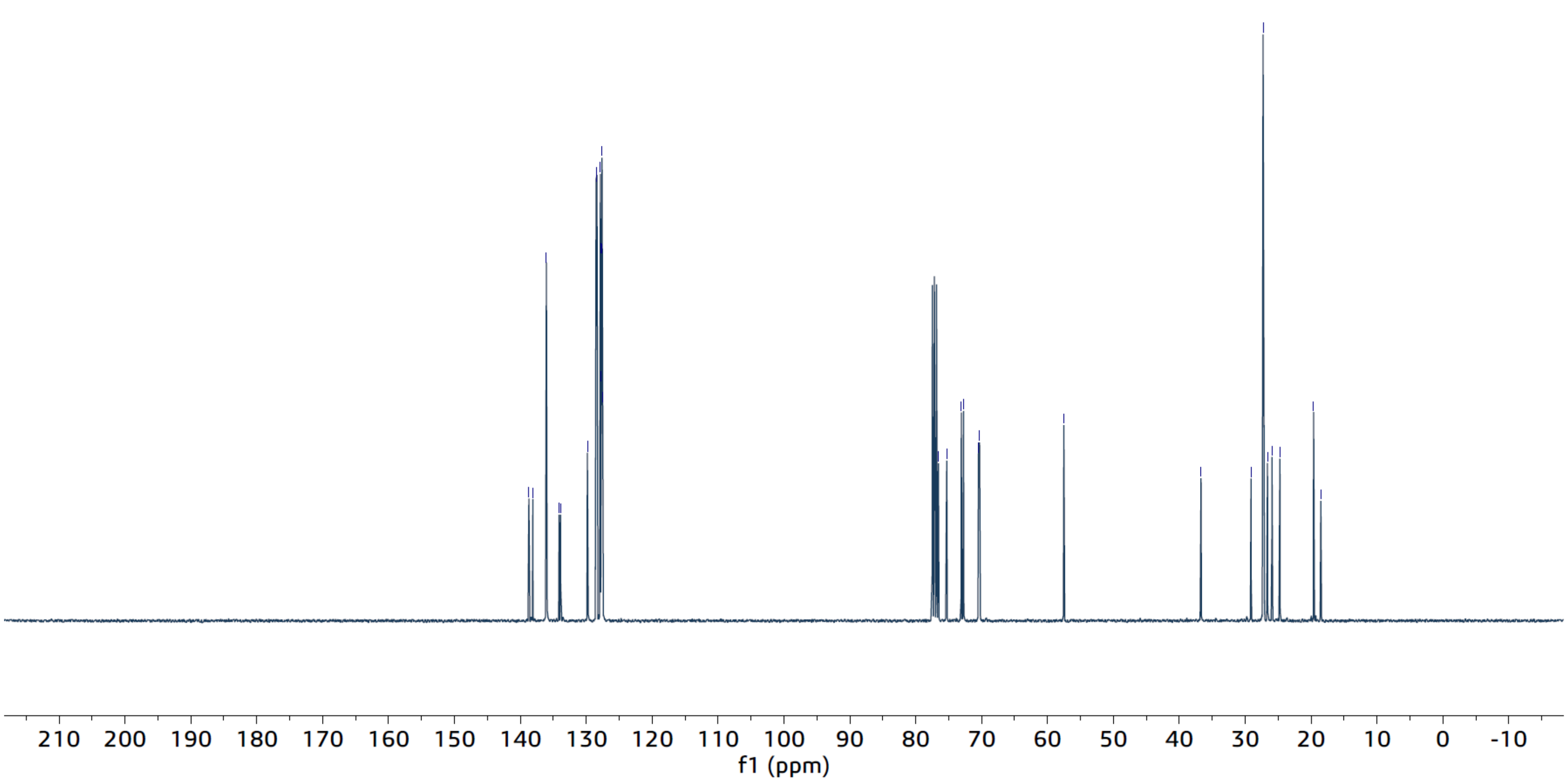

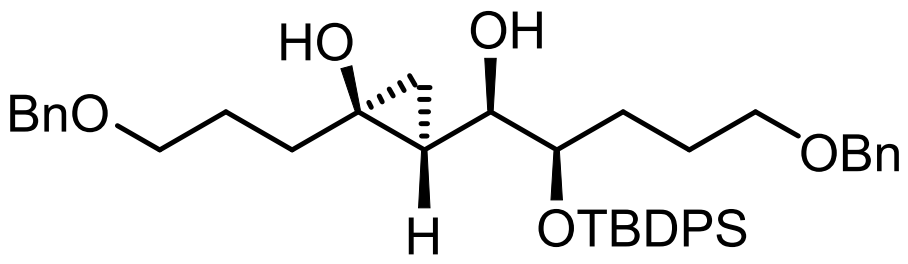

S2

^{1}H NMR (400 MHz, $CDCl_3$)

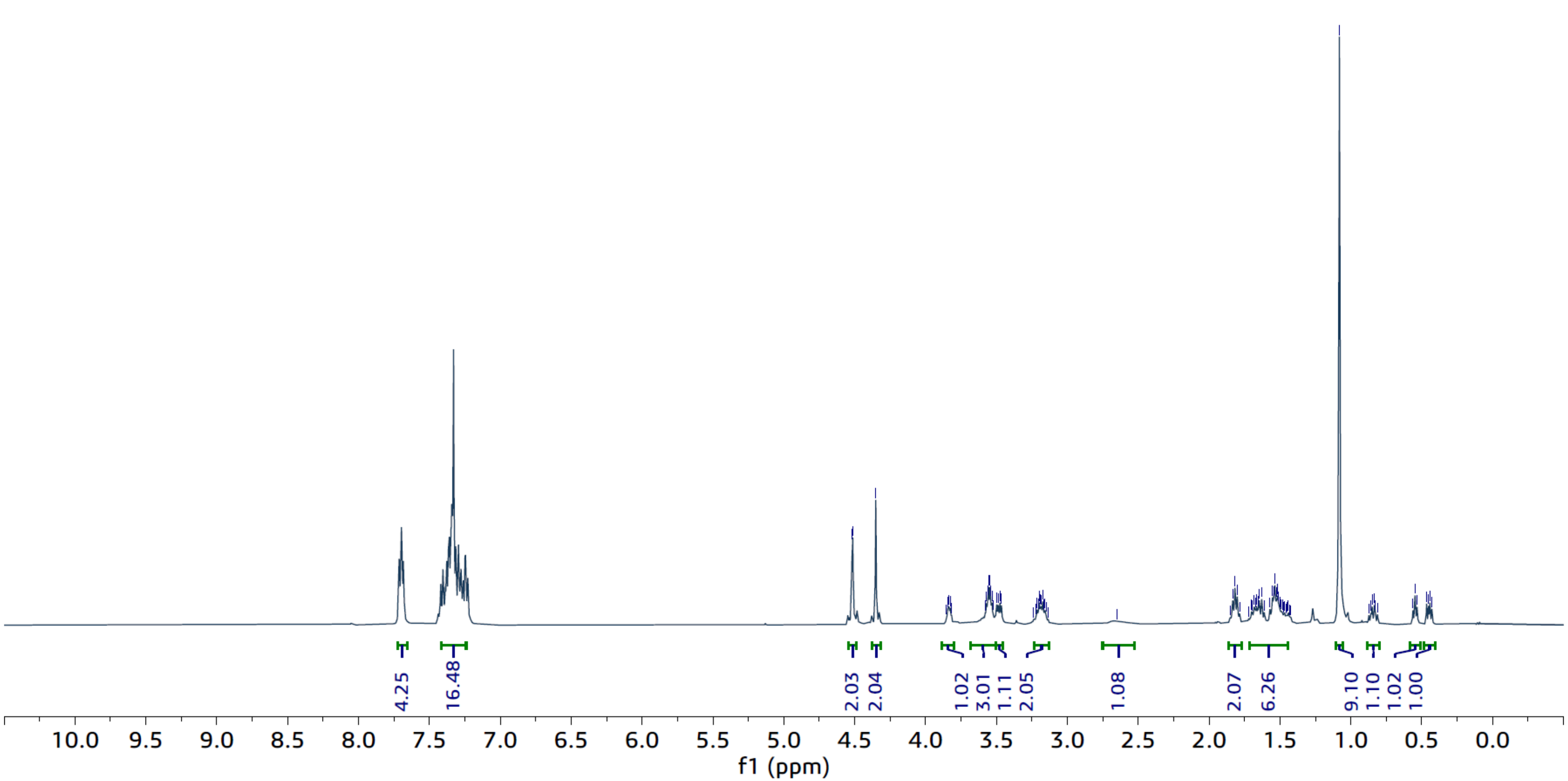

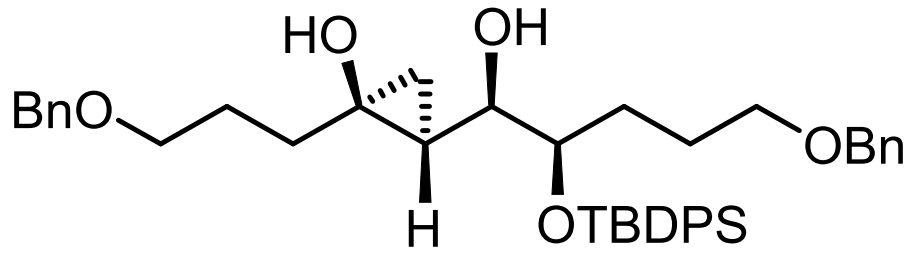

S2

^{13}C NMR (101 MHz, $CDCl_3$)

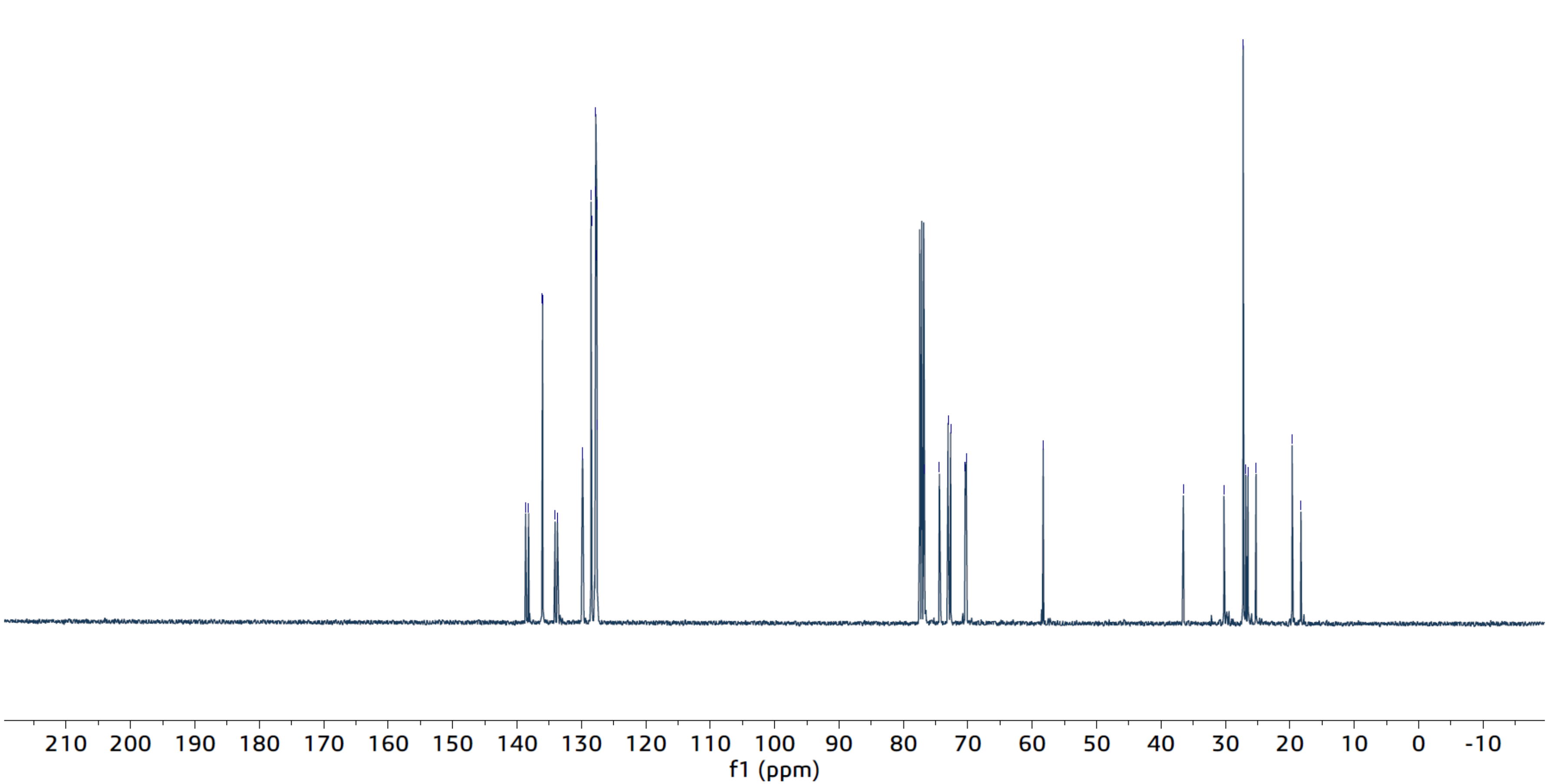

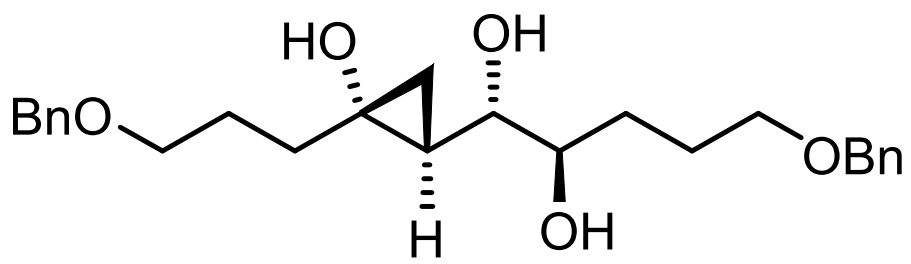

26

^{1}H NMR (400 MHz, $CDCl_3$)

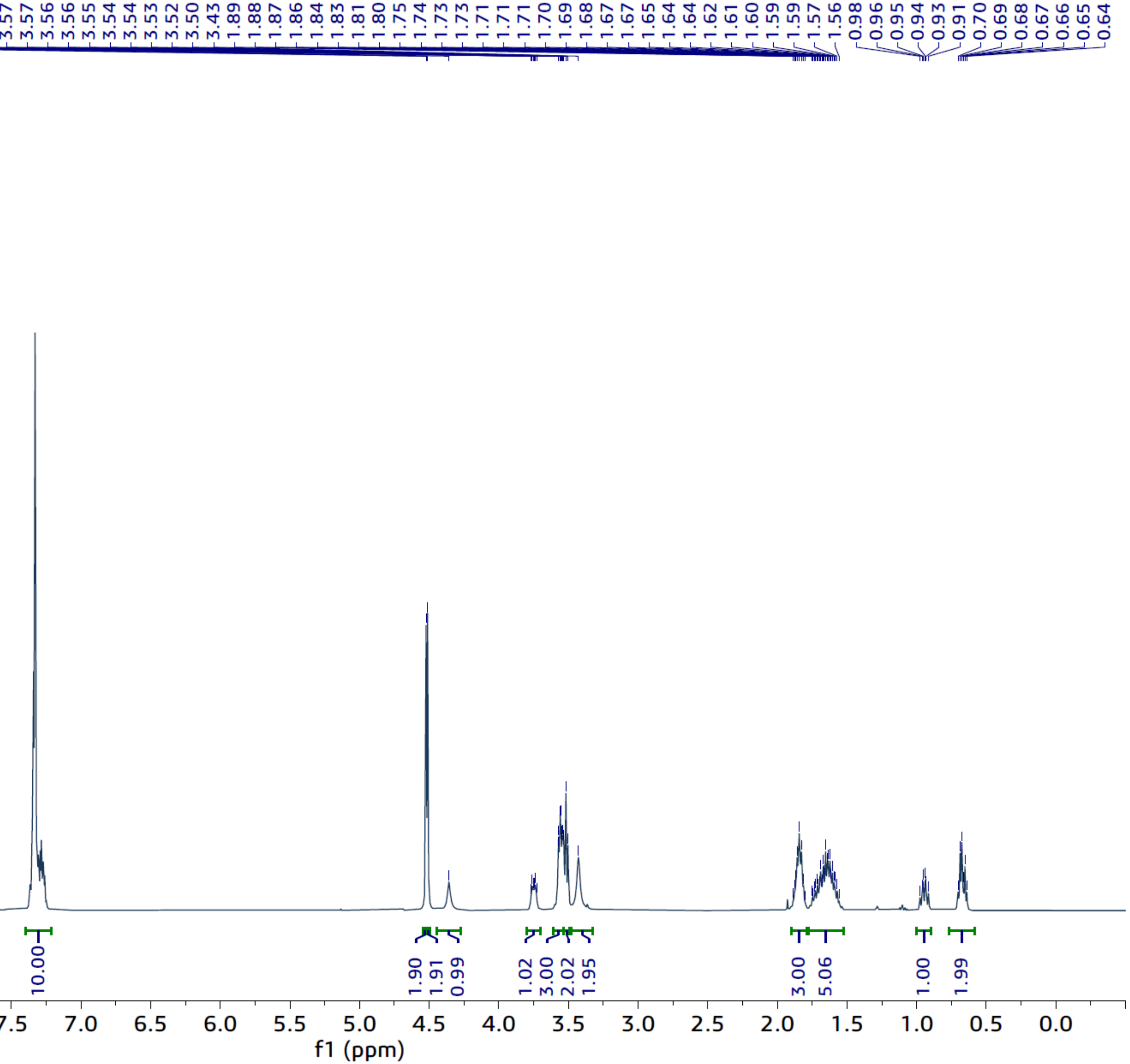

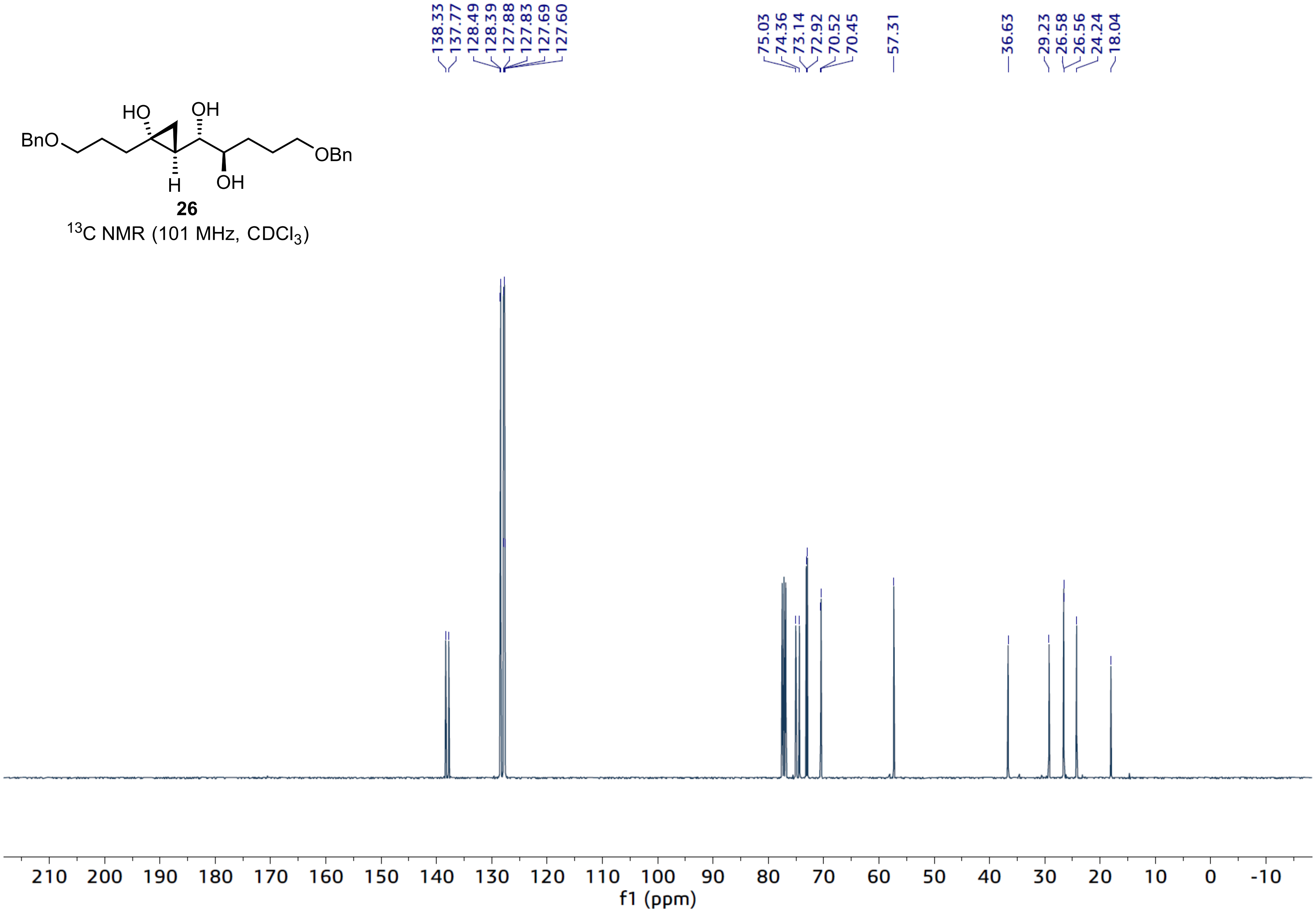

26

^{13}C NMR (101 MHz, $CDCl_3$)

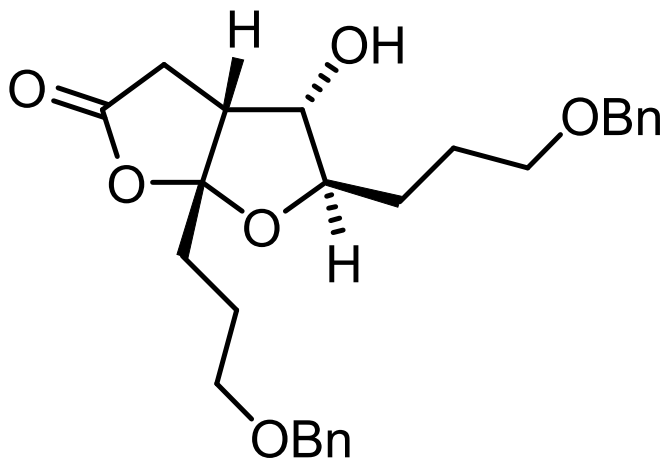

29

^{1}H NMR (400 MHz, $CDCl_3$)

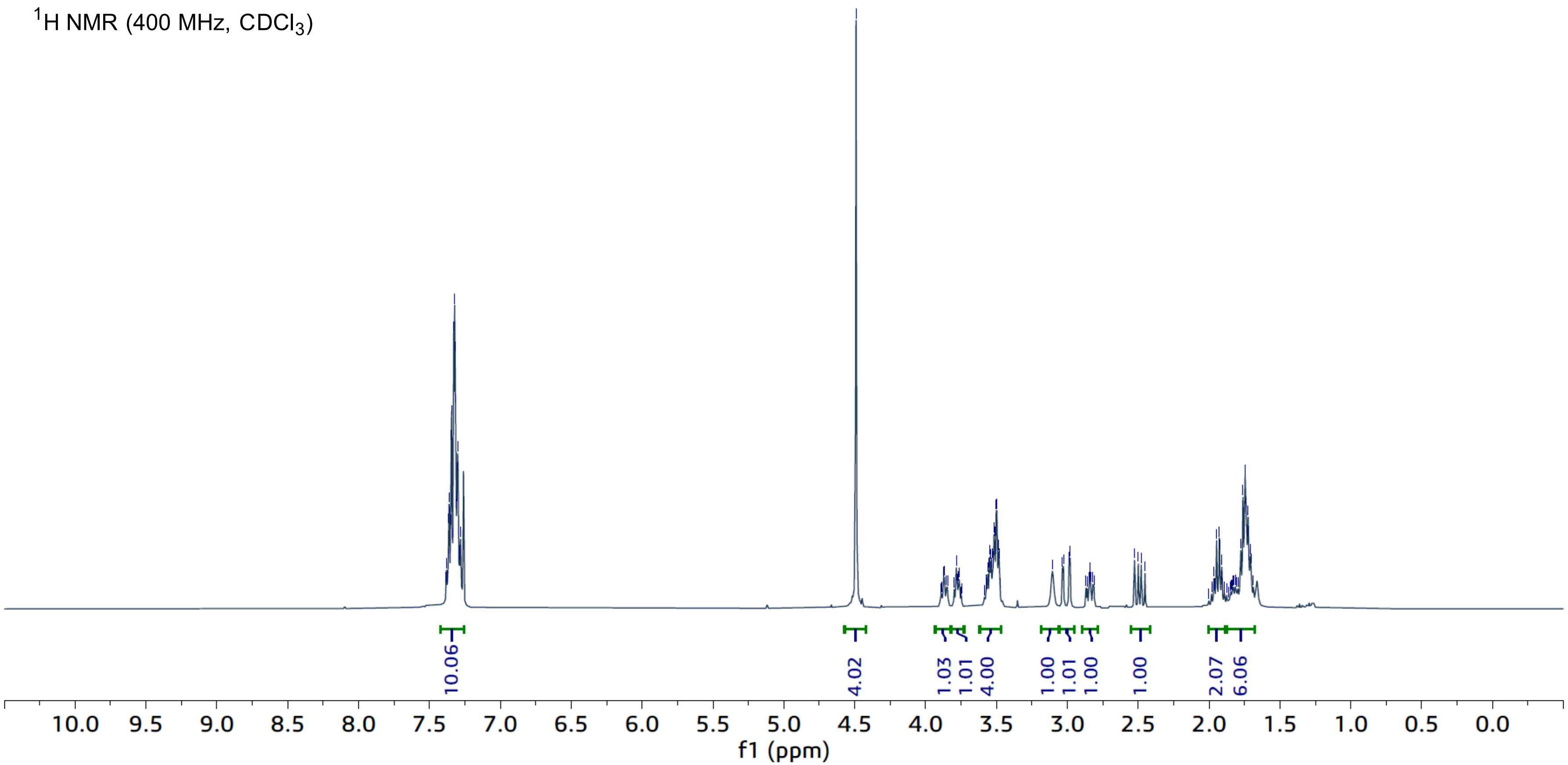

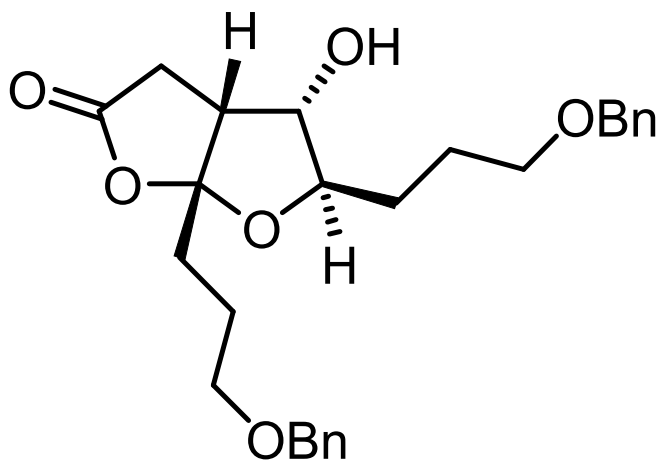

29

^{13}C NMR (101 MHz, $CDCl_3$)

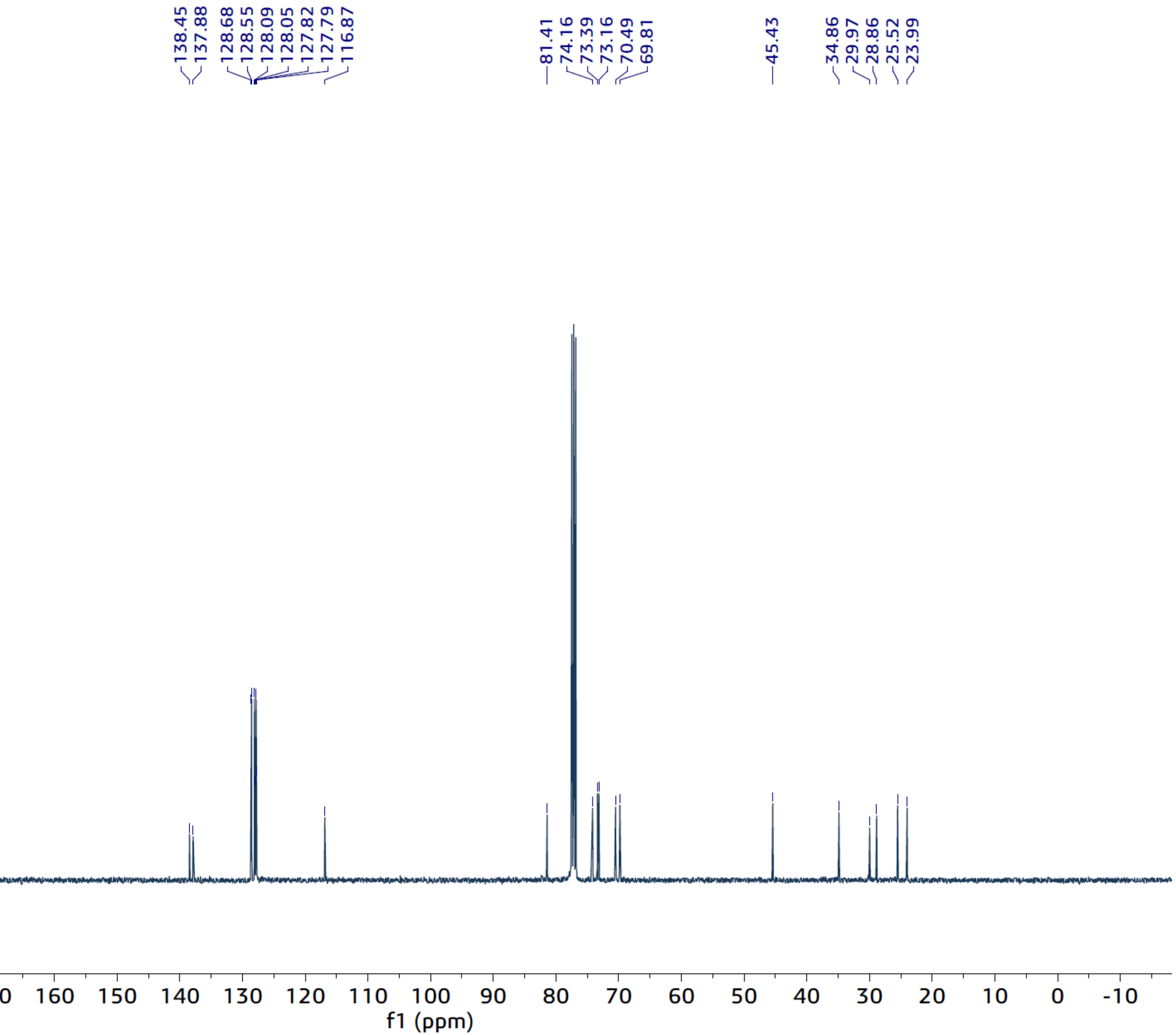

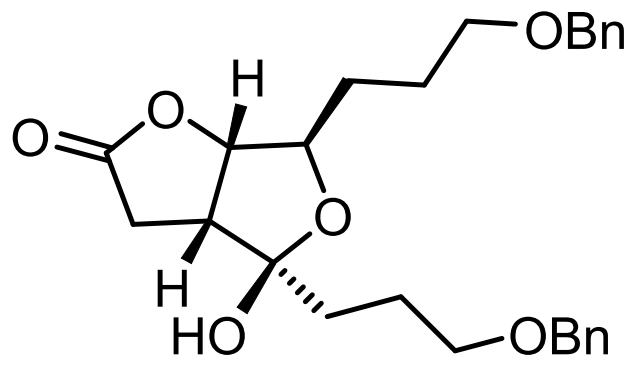

28

^{1}H NMR (400 MHz, $CDCl_3$)

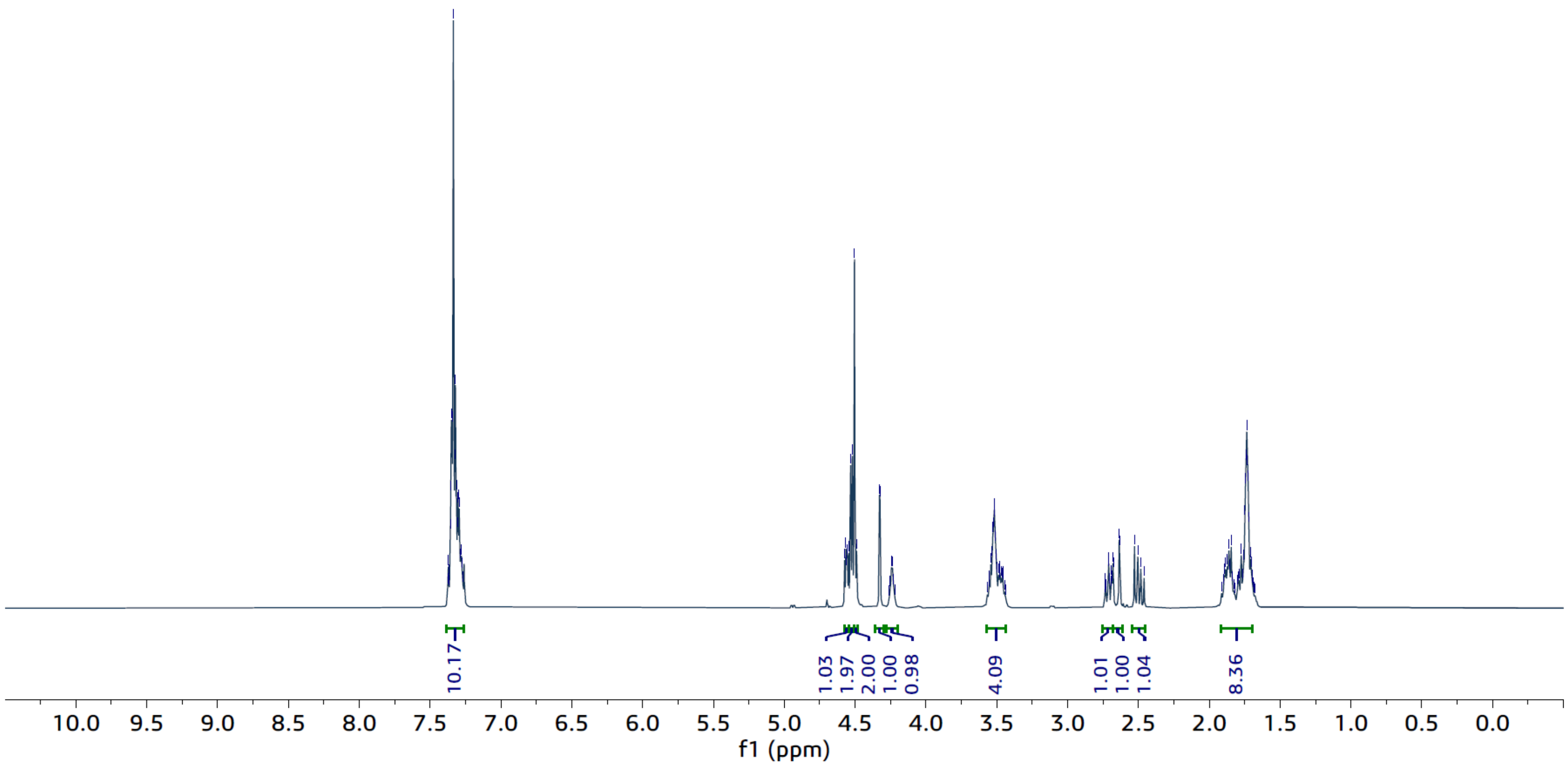

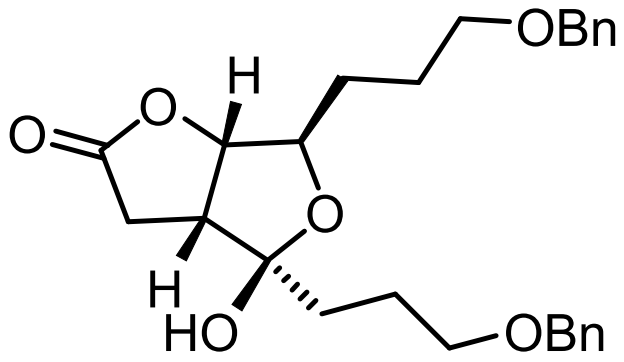

28

^{13}C NMR (101 MHz, $CDCl_3$)

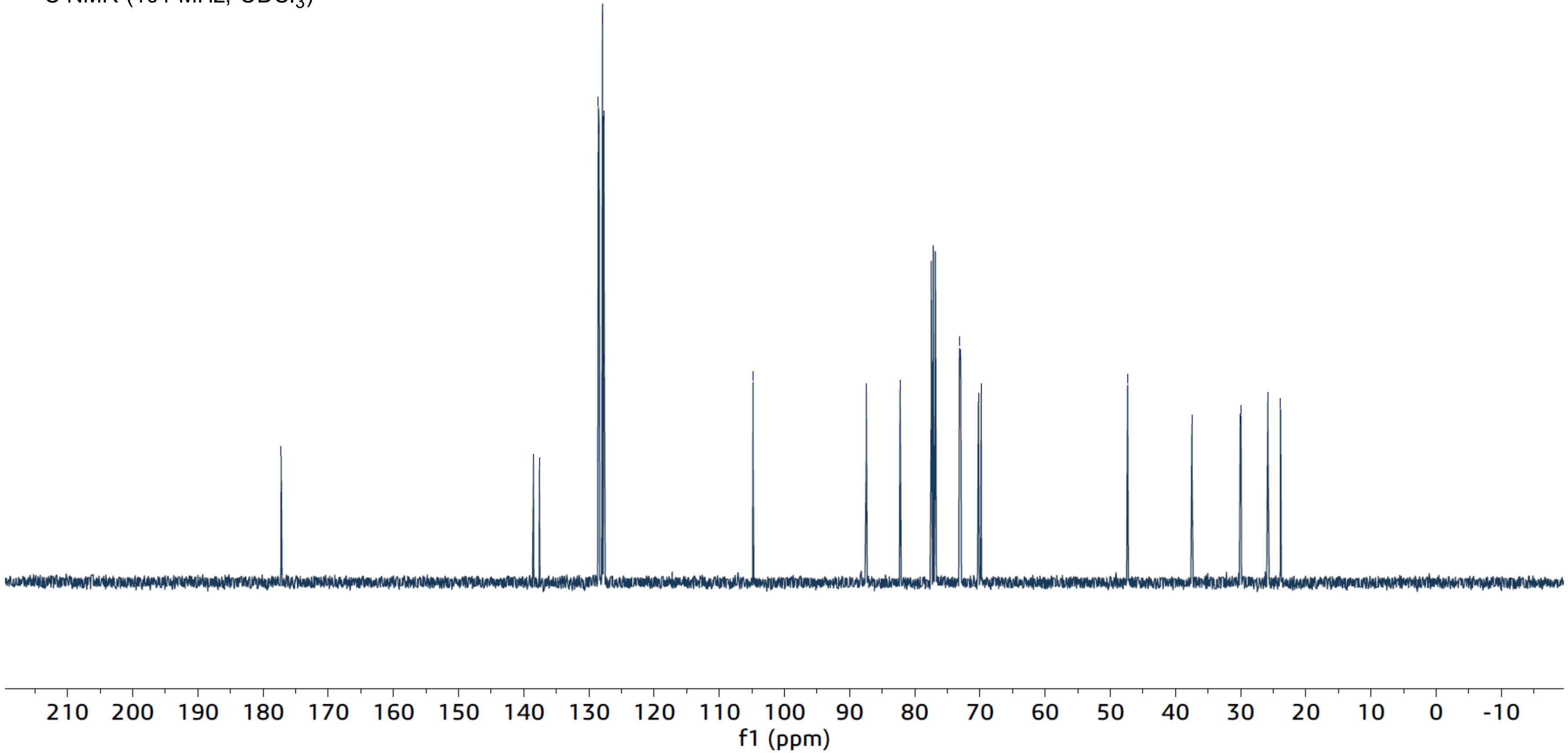

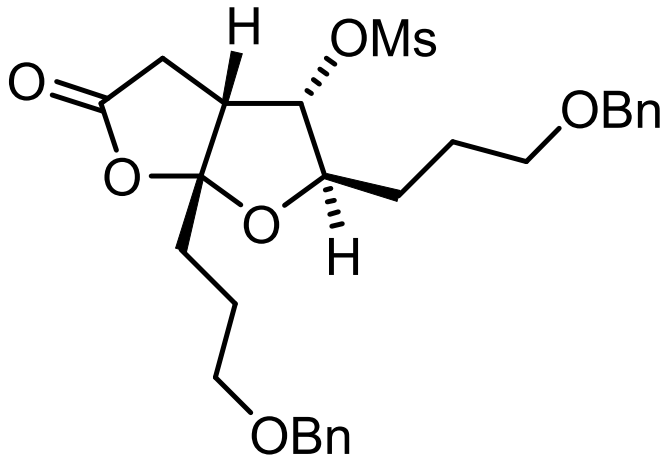

30

^{1}H NMR (400 MHz, $CDCl_3$)

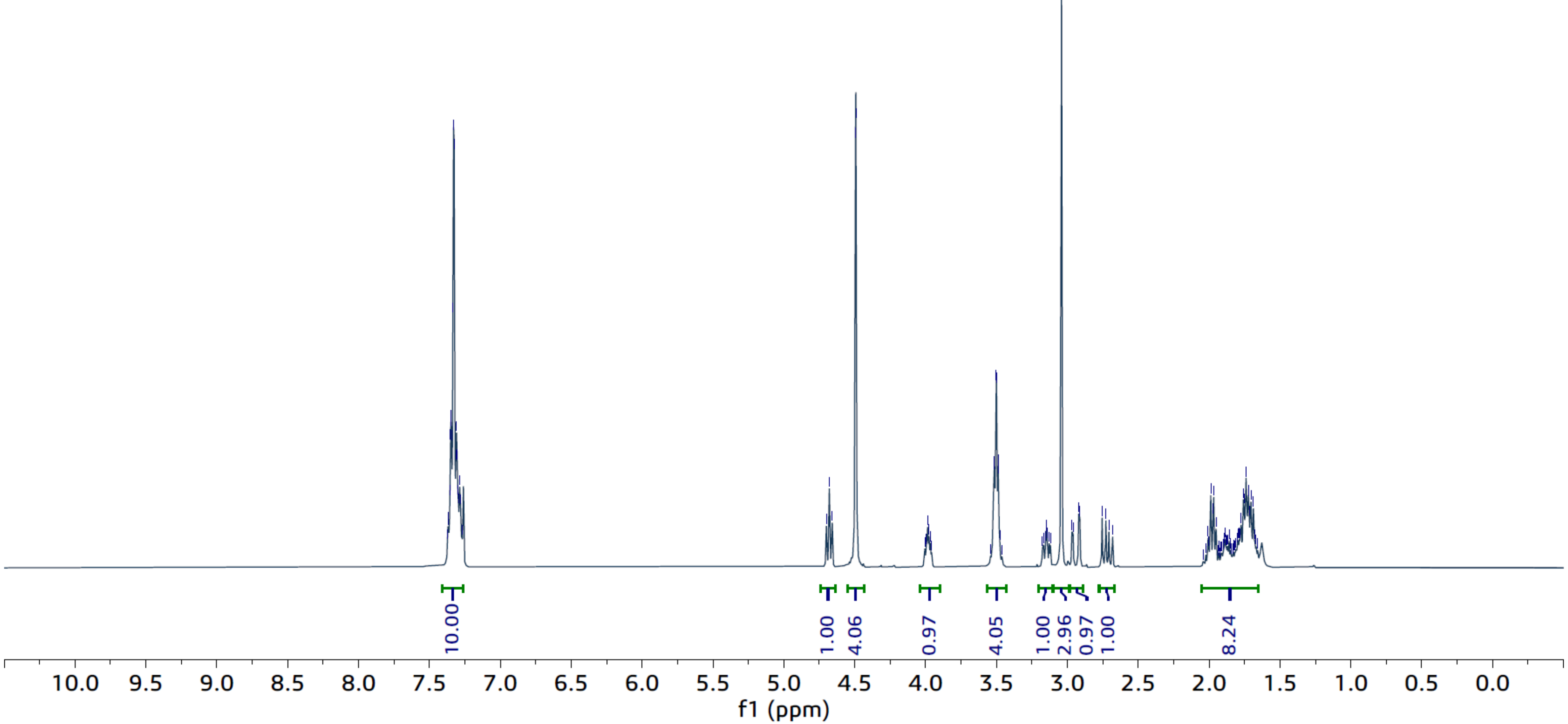

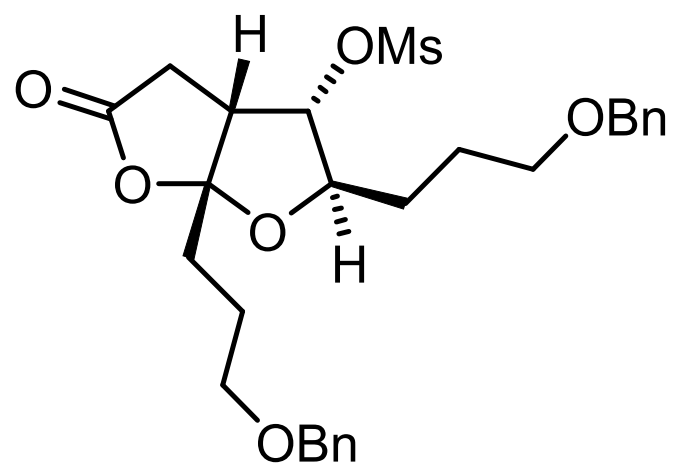

30

^{13}C NMR (101 MHz, $CDCl_3$)

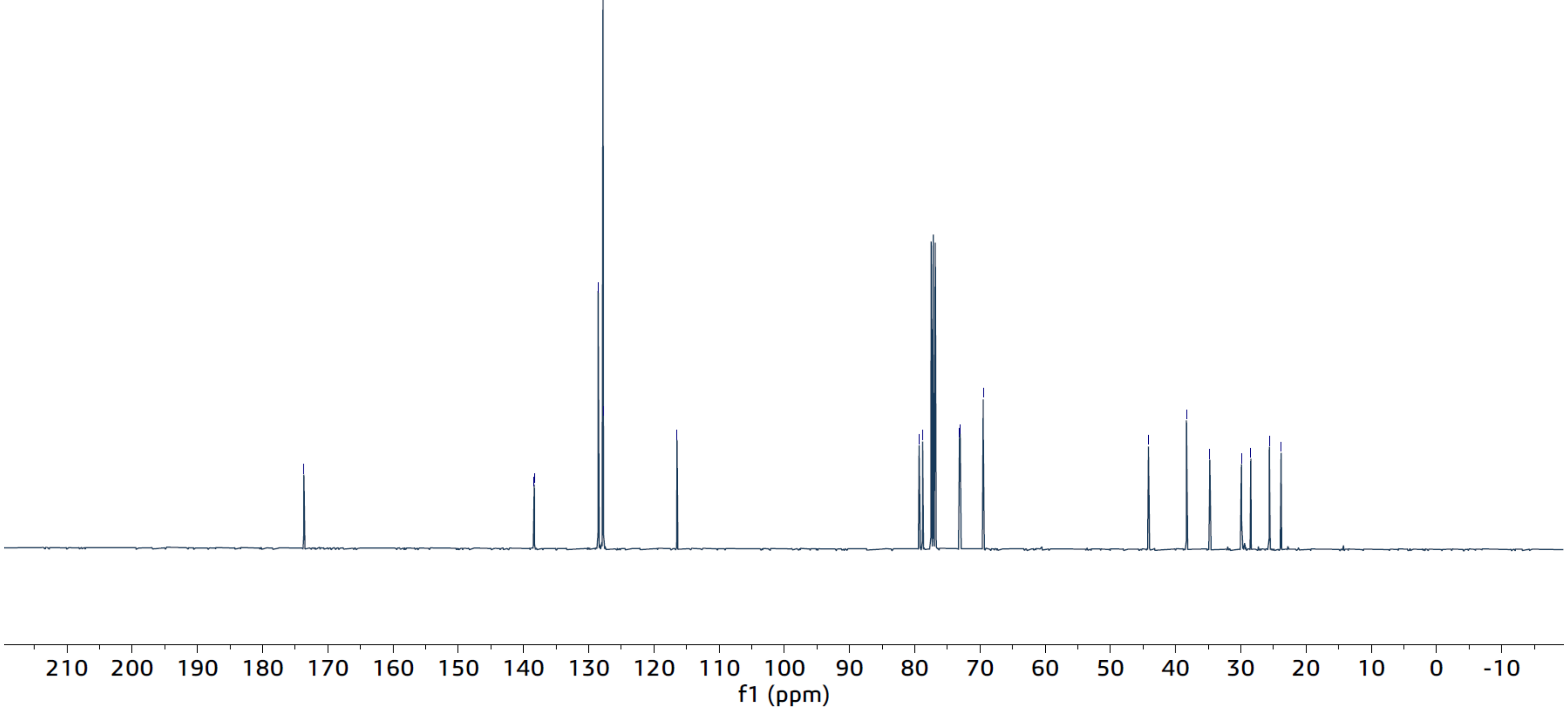

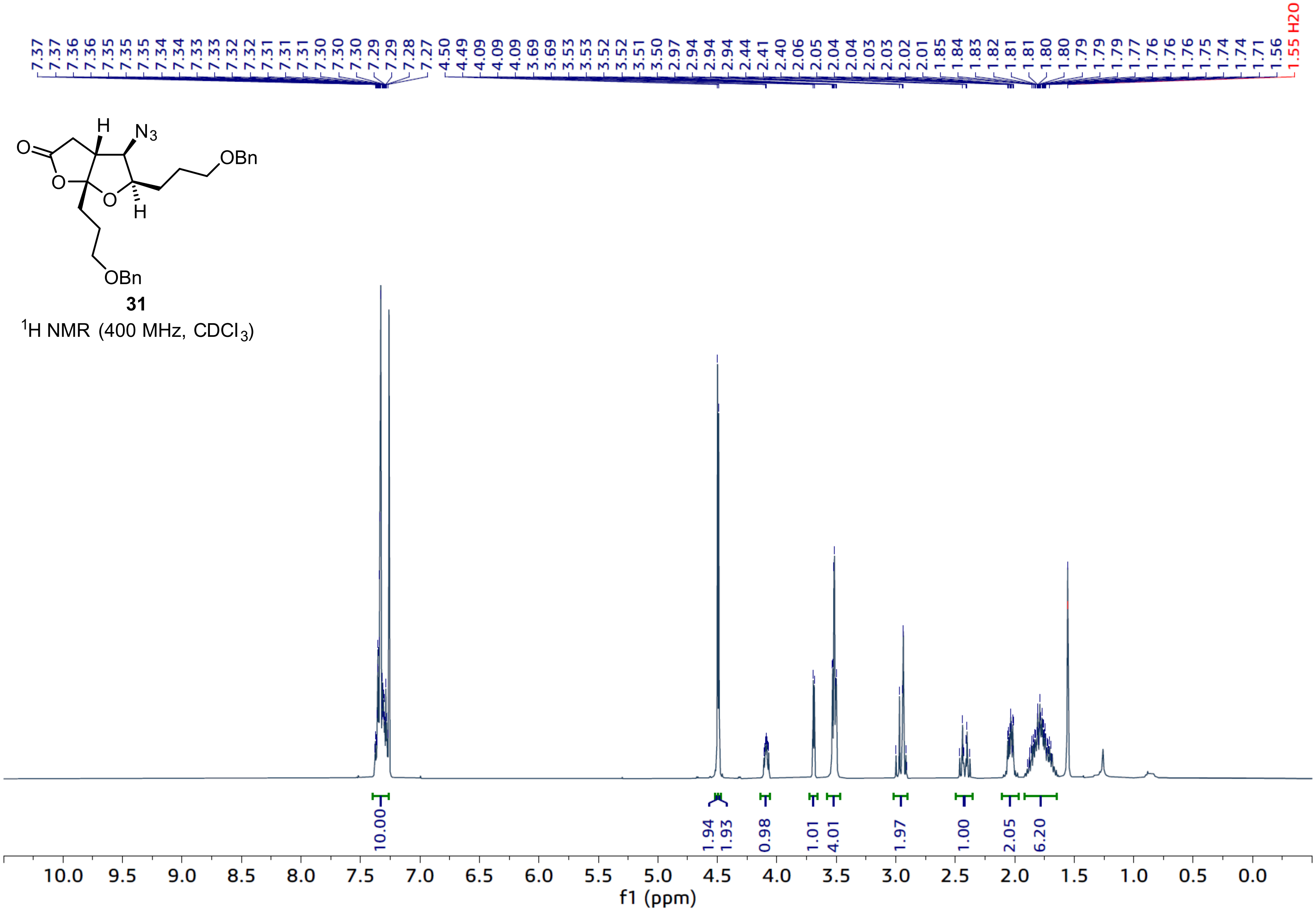
7.37 7.37 7.36 7.36 7.35 7.35 7.35 7.34 7.34 7.33 7.33 7.32 7.32 7.31 7.31 7.31 7.30 7.30 7.30 7.29 7.29 7.28 7.27 4.50 4.49 4.09 4.09 4.09 3.69 3.69 3.53 3.53 3.52 3.52 3.51 3.50 2.97 2.94 2.94 2.94 2.44 2.41 2.40 2.06 2.05 2.04 2.04 2.03 2.03 2.02 2.01 1.85 1.84 1.83 1.82 1.81 1.81 1.80 1.80 1.79 1.79 1.79 1.77 1.76 1.76 1.75 1.74 1.74 1.71 1.56
1.55 H2O
O
H
N3
OBn
O
O
H
OBn
31
1H NMR (400 MHz, CDCl3)
10.00
1.94
1.93
0.98
1.01
4.01
1.97
1.00
2.05
6.20
10.0 9.5 9.0 8.5 8.0 7.5 7.0 6.5 6.0 5.5 5.0 4.5 4.0 3.5 3.0 2.5 2.0 1.5 1.0 0.5 0.0
f1 (ppm)

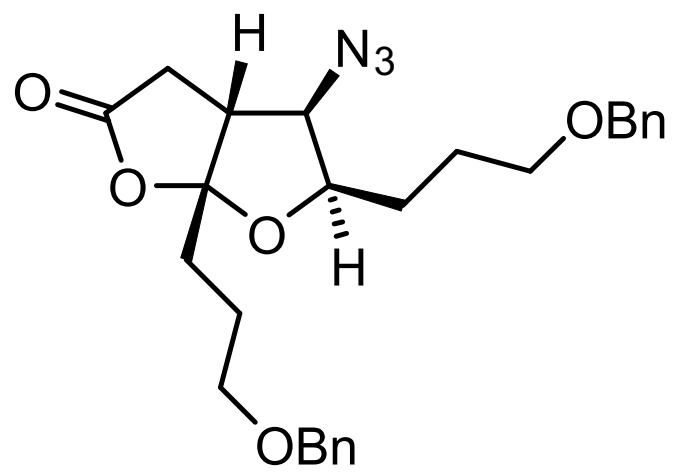

31

^{13}C NMR (101 MHz, $CDCl_3$)

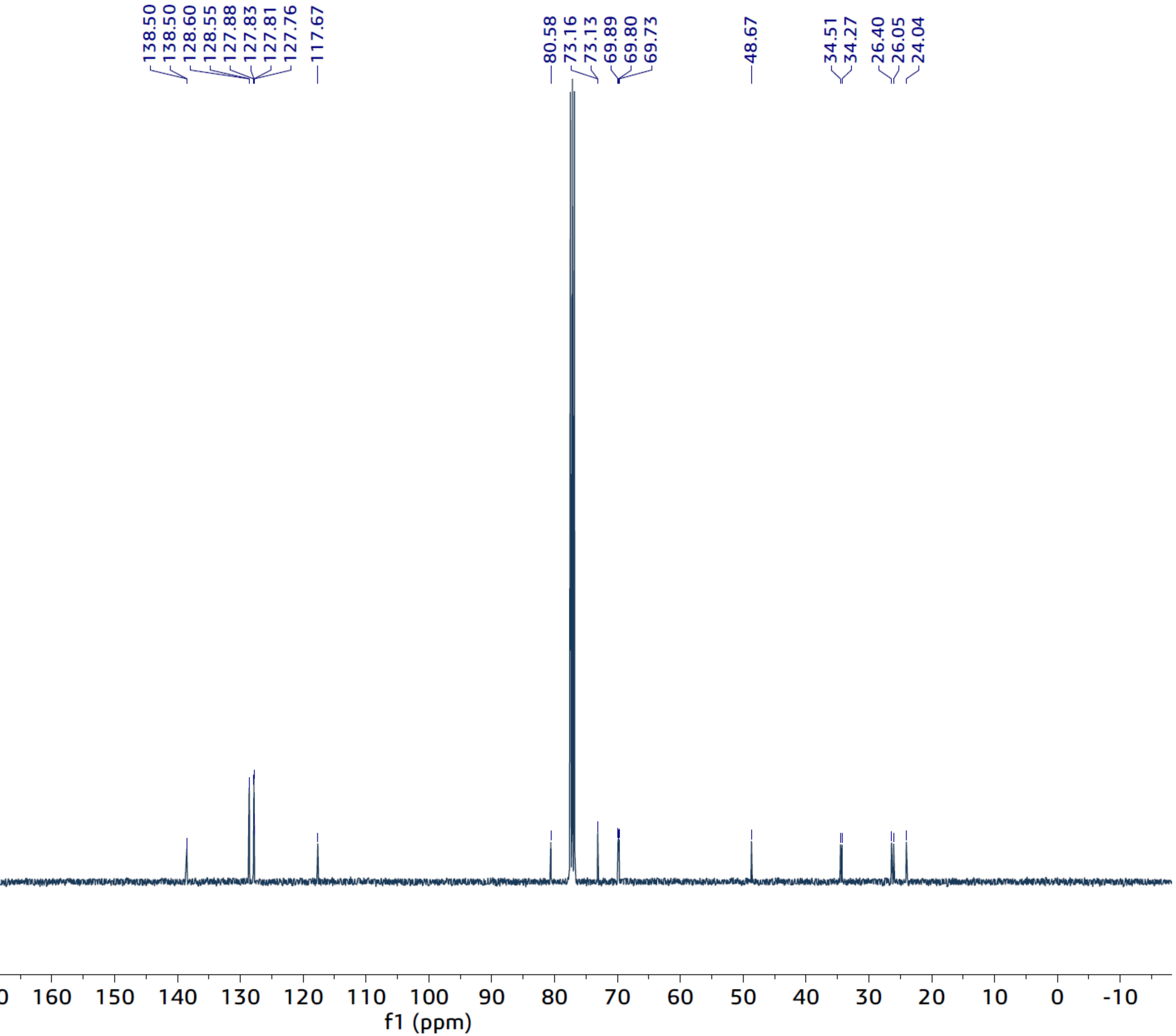

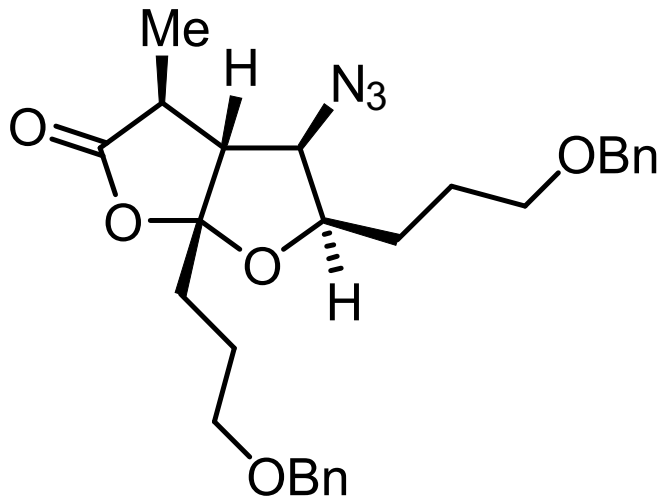

32

^{1}H NMR (600 MHz, $CDCl_3$)

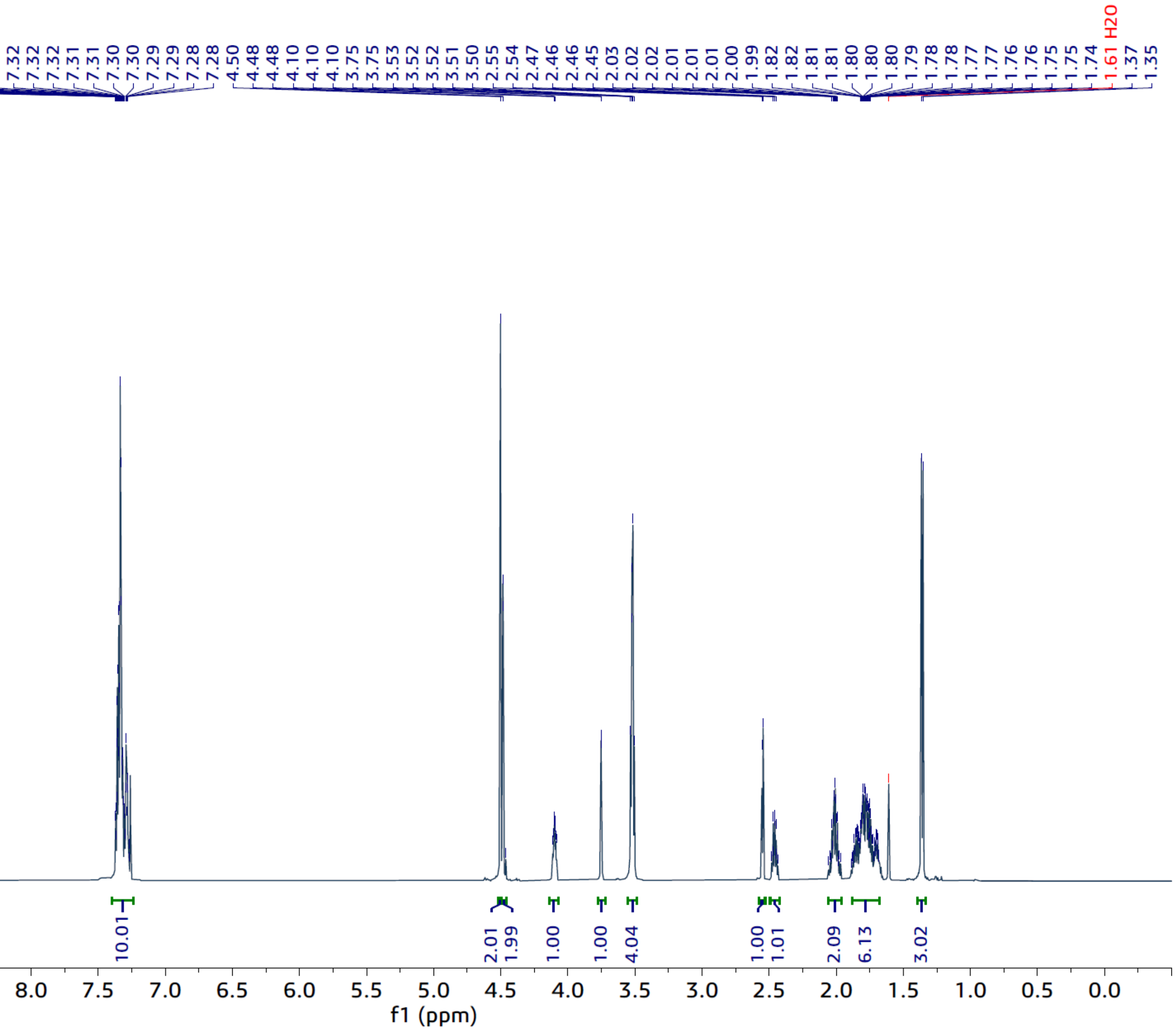

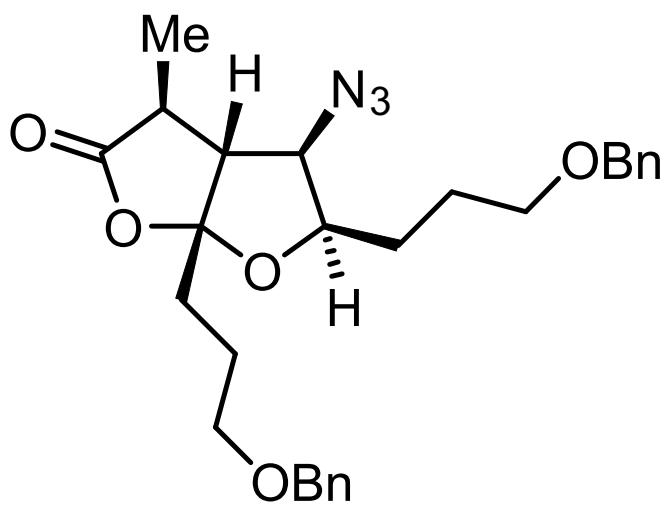

32

^{13}C NMR (151 MHz, $CDCl_3$)

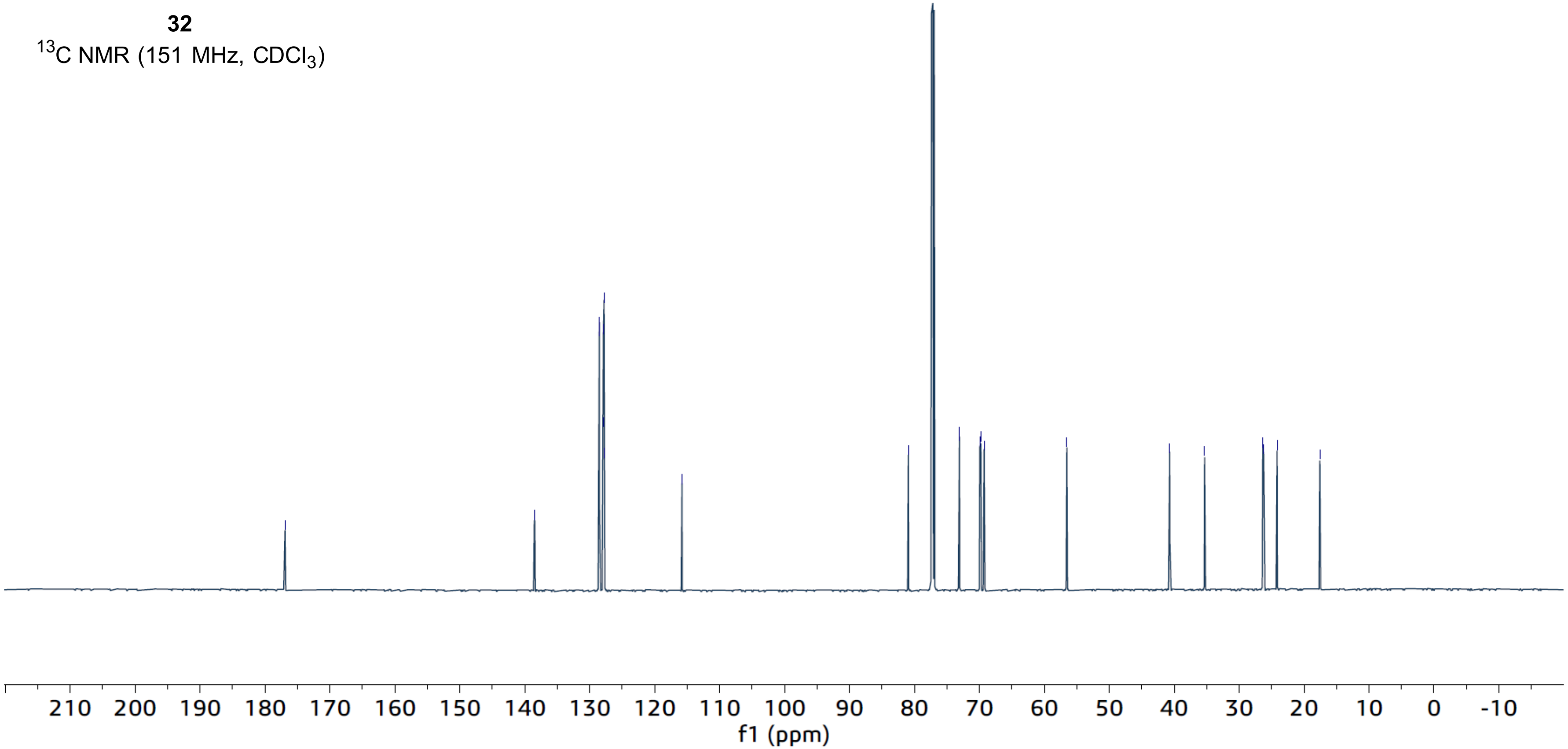

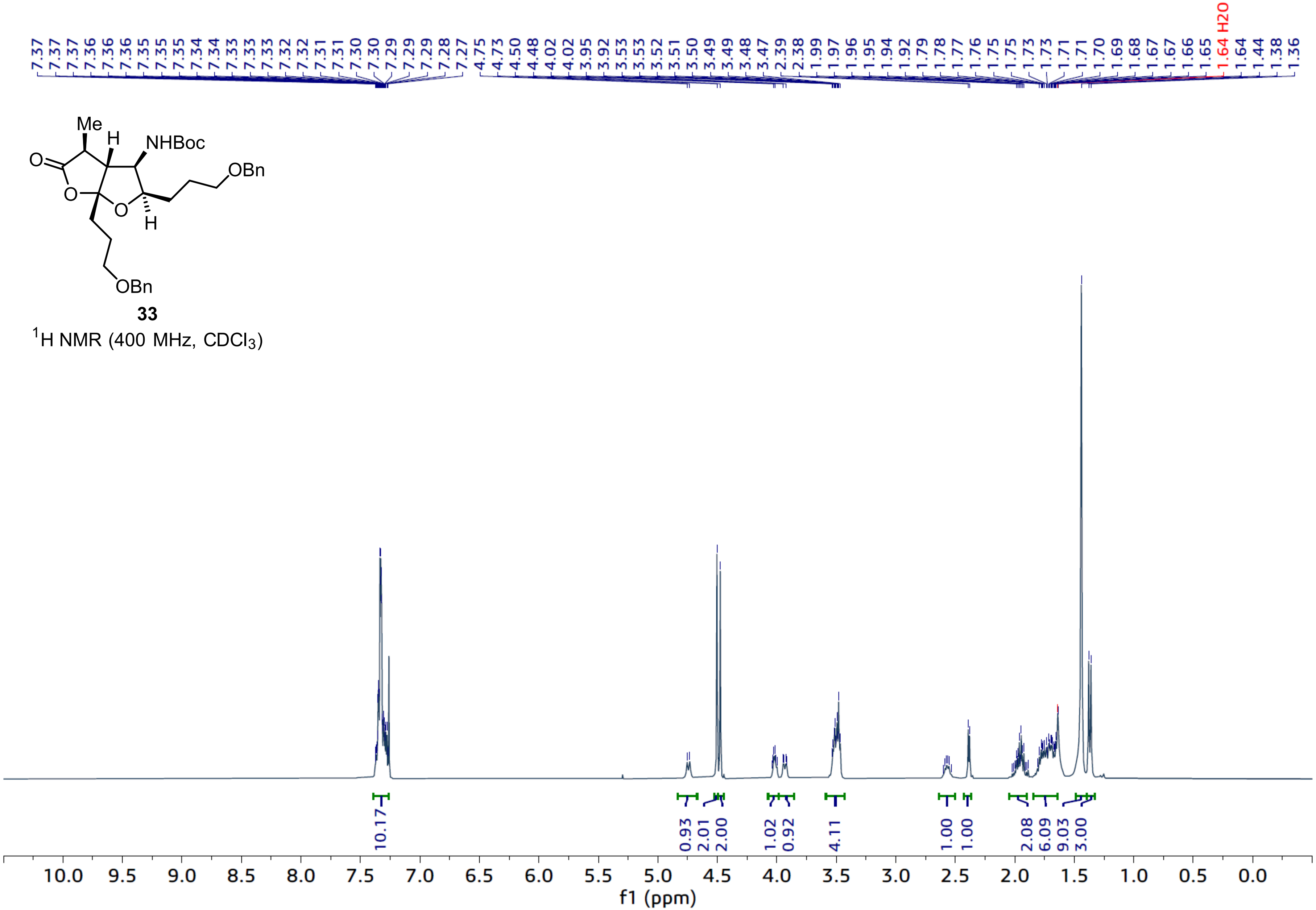
7.37 7.37 7.37 7.36 7.36 7.36 7.35 7.35 7.35 7.34 7.34 7.33 7.33 7.33 7.32 7.32 7.31 7.31 7.30 7.30 7.29 7.29 7.29 7.28 7.27 4.75 4.73 4.50 4.48 4.02 4.02 3.95 3.92 3.53 3.53 3.52 3.51 3.50 3.49 3.49 3.48 3.47 2.39 2.38 1.99 1.97 1.96 1.95 1.94 1.92 1.79 1.78 1.77 1.76 1.75 1.75 1.73 1.73 1.71 1.71 1.70 1.69 1.68 1.67 1.67 1.66 1.65 1.64 H2O 1.64 1.44 1.38 1.36
Me
H
NHBoc
O
O
O
OBn
H
OBn
33
1H NMR (400 MHz, CDCl3)
10.17
0.93 2.01 2.00
1.02 0.92
4.11
1.00 1.00
2.08 6.09 9.03 3.00
10.0 9.5 9.0 8.5 8.0 7.5 7.0 6.5 6.0 5.5 5.0 4.5 4.0 3.5 3.0 2.5 2.0 1.5 1.0 0.5 0.0
f1 (ppm)

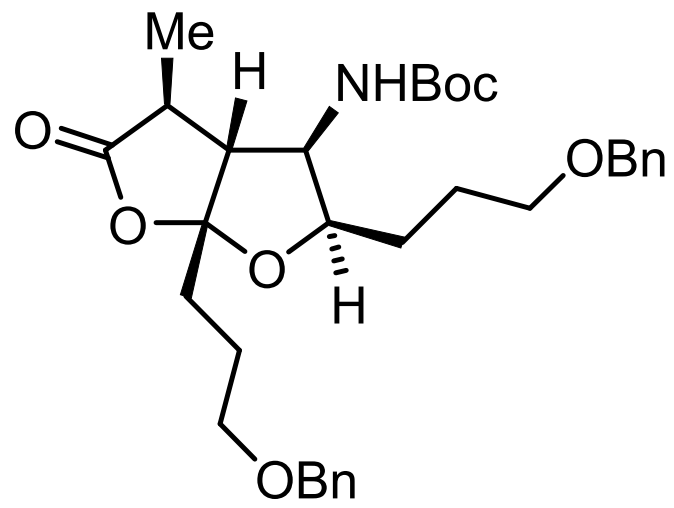

33

^{13}C NMR (101 MHz, $CDCl_3$)

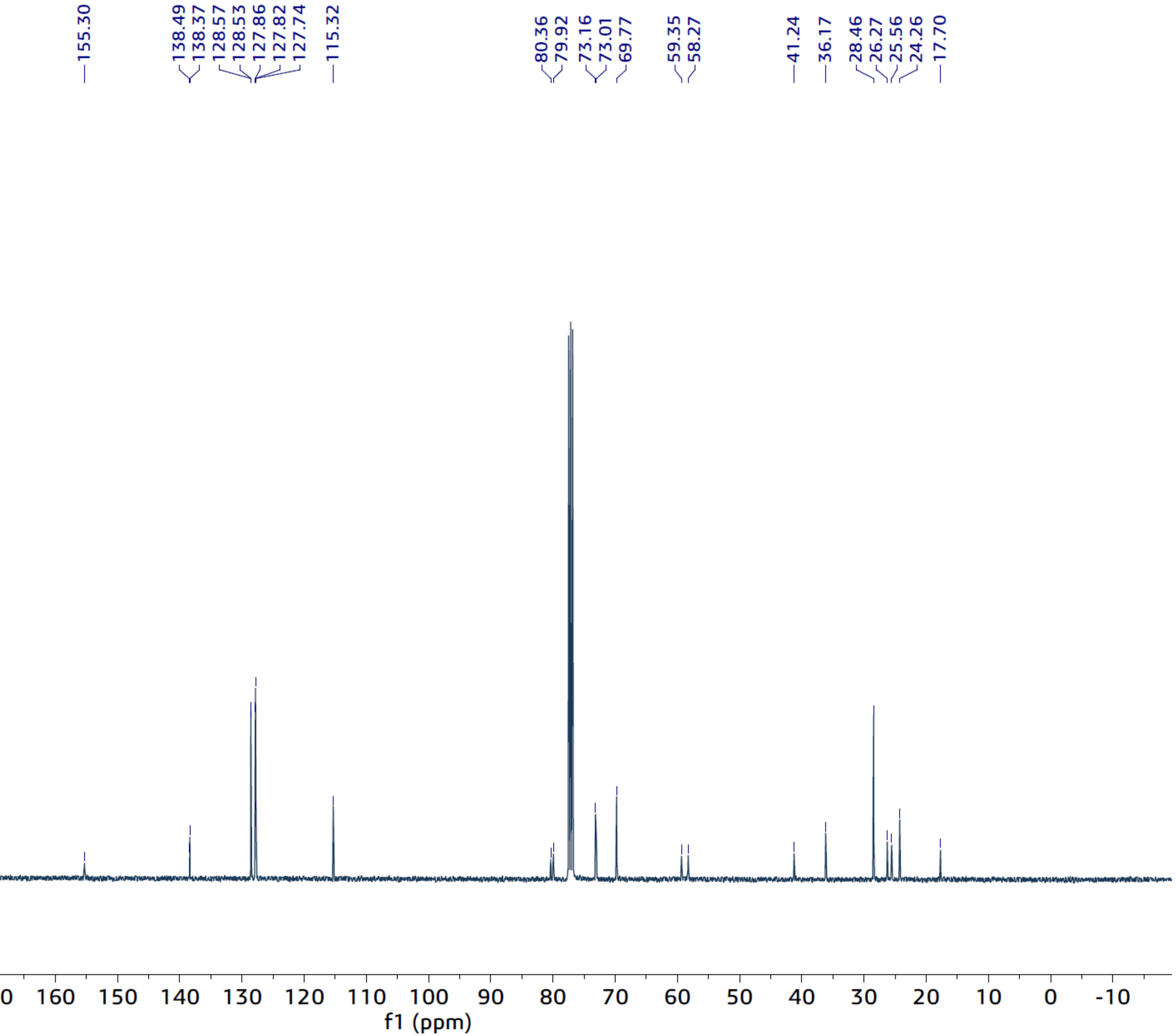

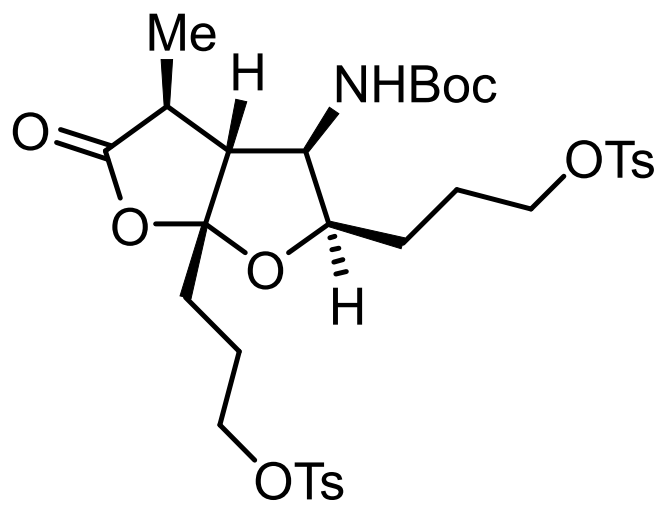

S3

^{1}H NMR (400 MHz, $CDCl_3$)

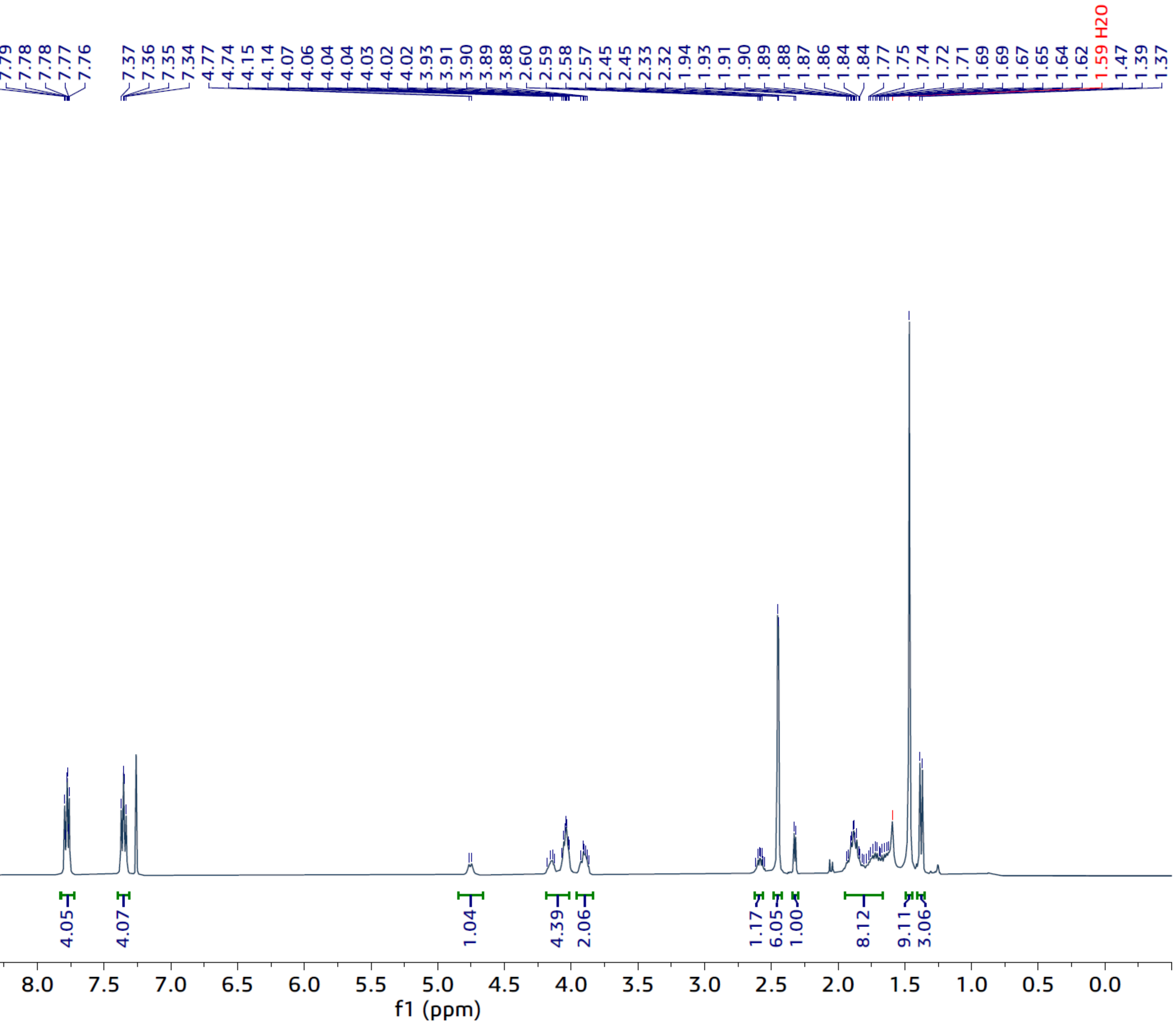

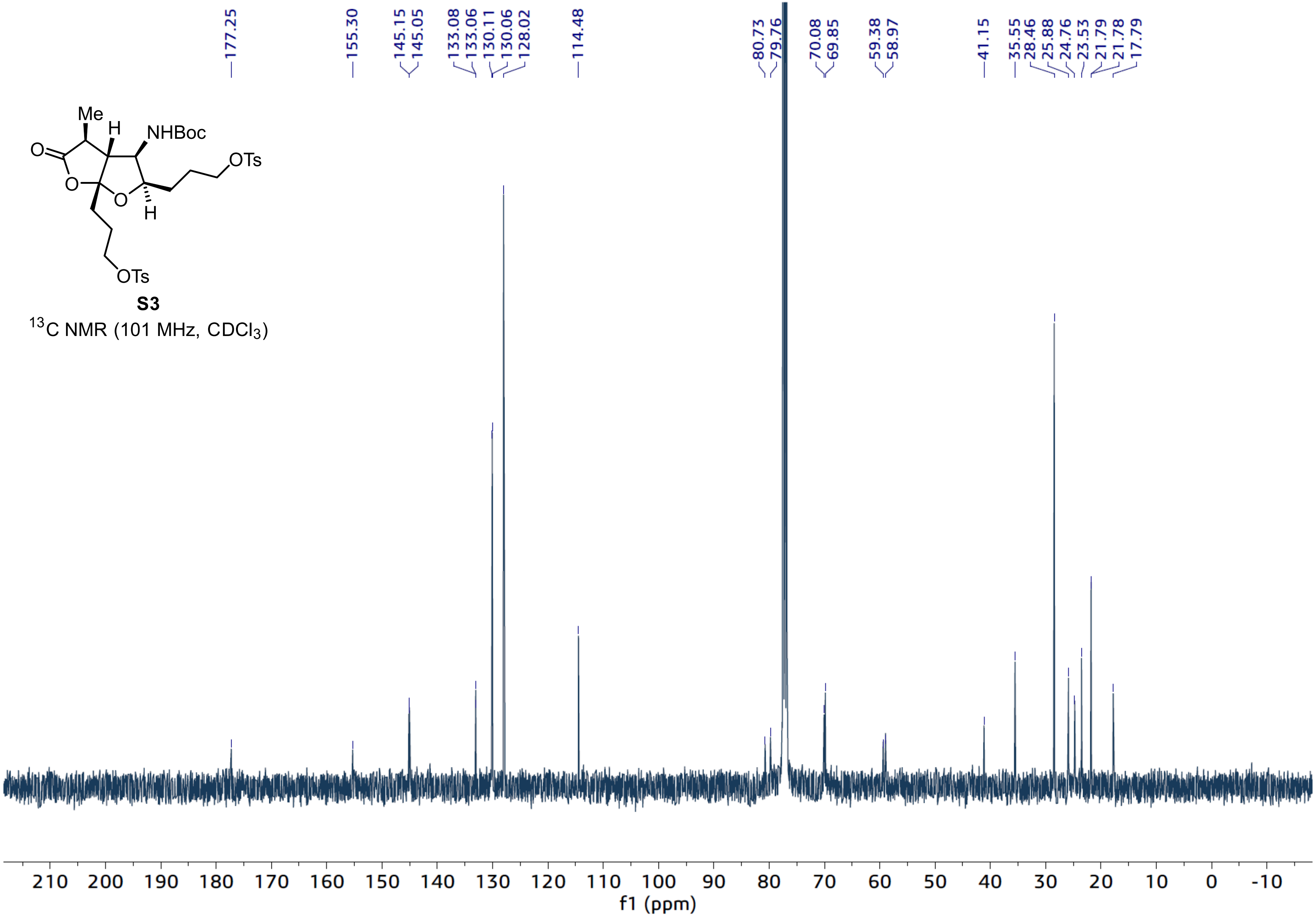

S3

^{13}C NMR (101 MHz, $CDCl_3$)

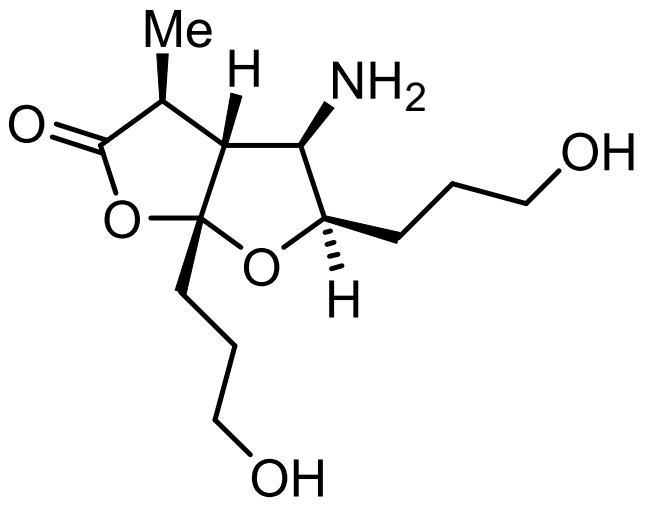

34 (crude)

^{1}H NMR (400 MHz, MeOD)

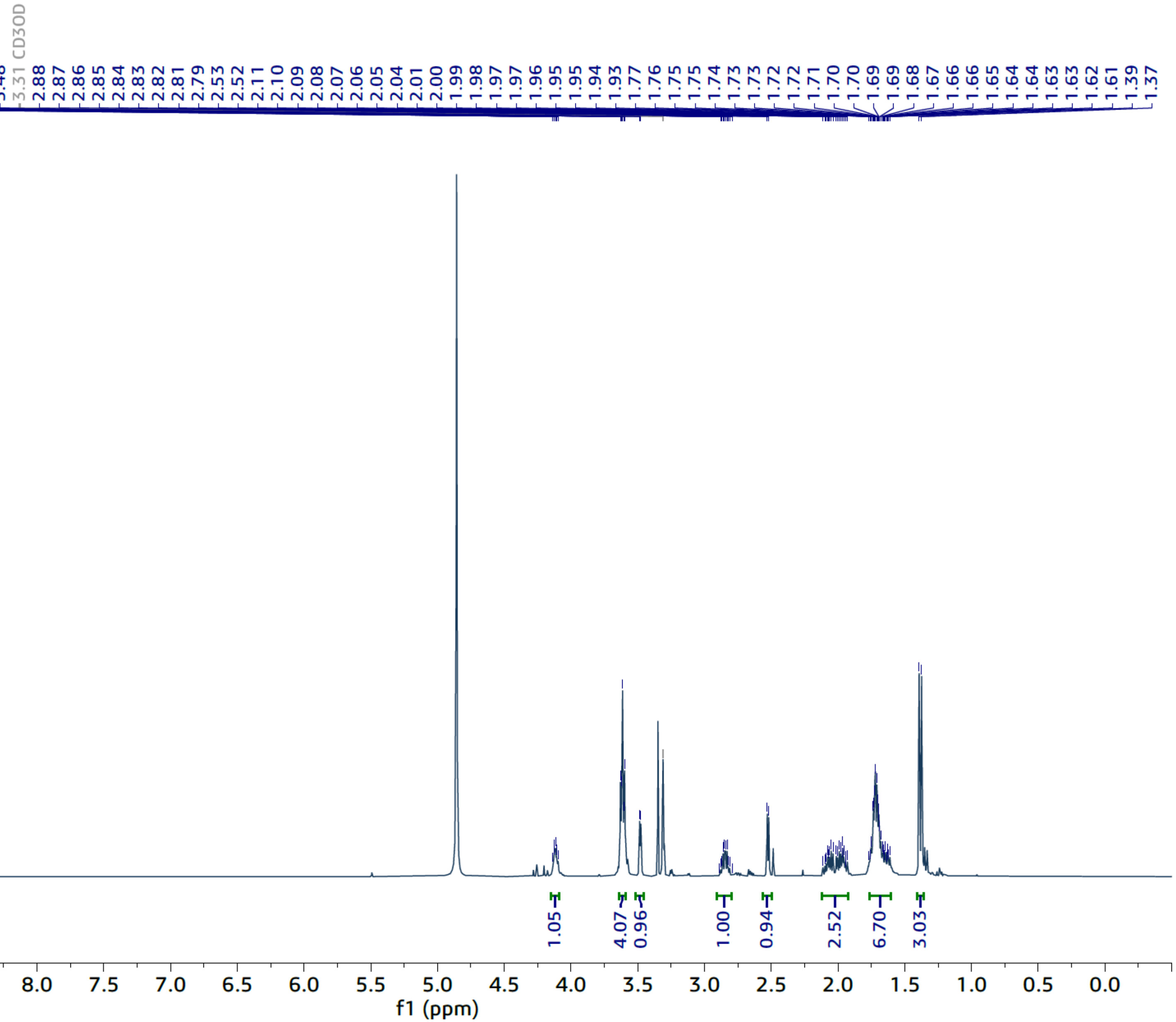

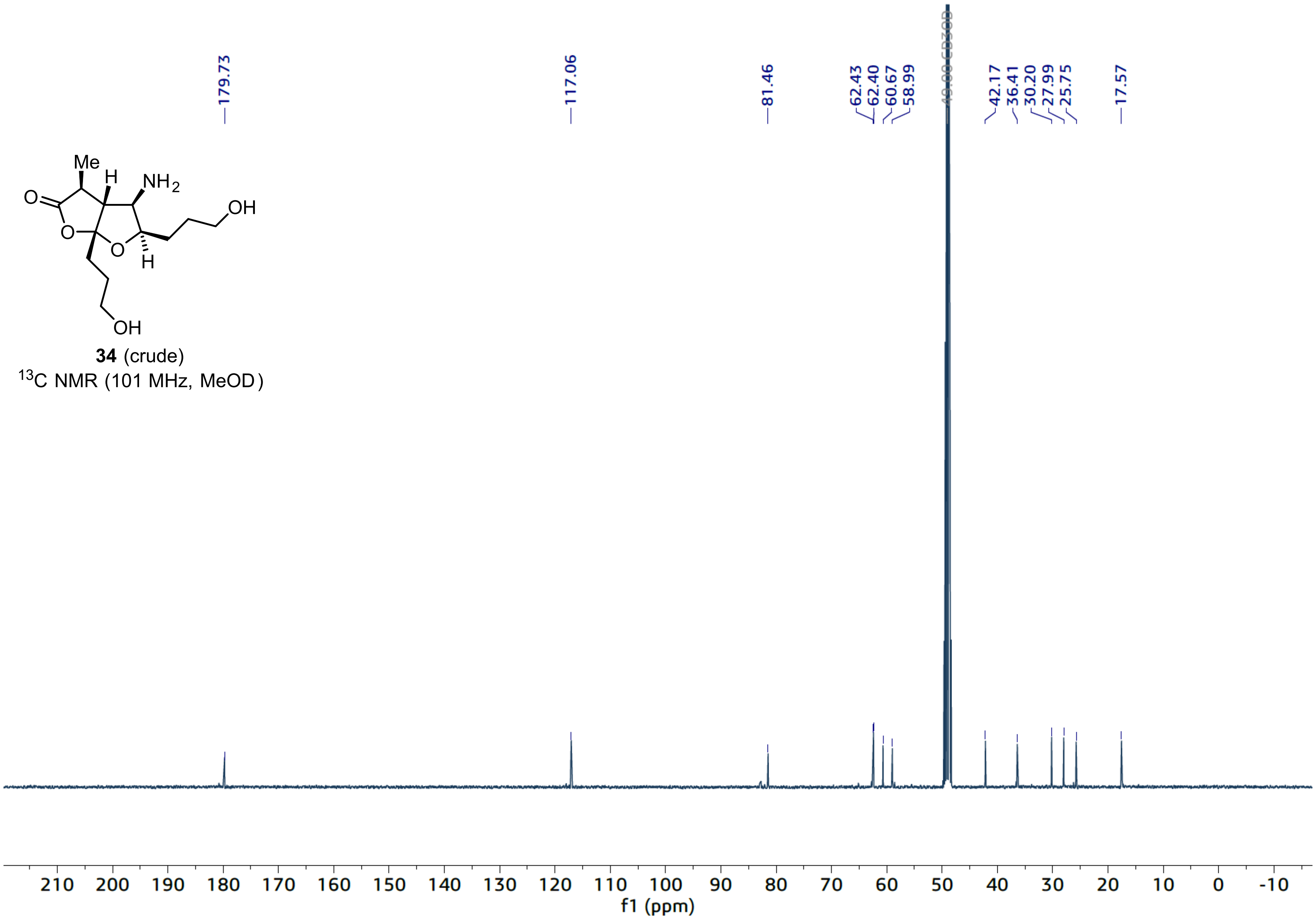

34 (crude)

^{13}C NMR (101 MHz, MeOD)

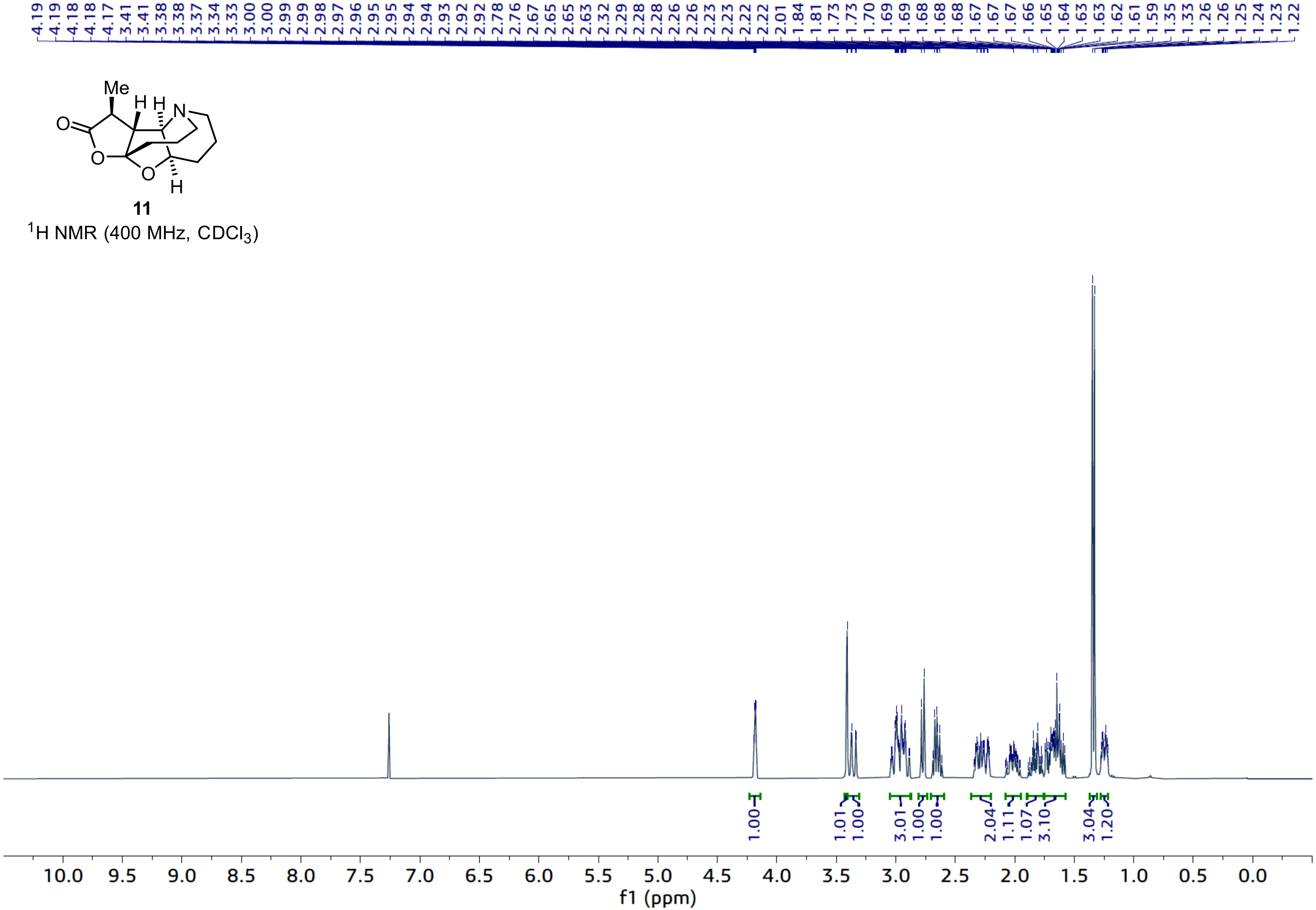

11

^{1}H NMR (400 MHz, $CDCl_3$)

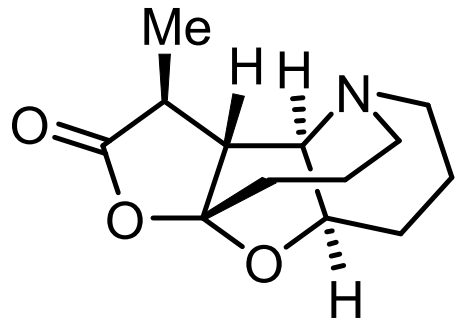

11

^{13}C NMR (101 MHz, $CDCl_3$)

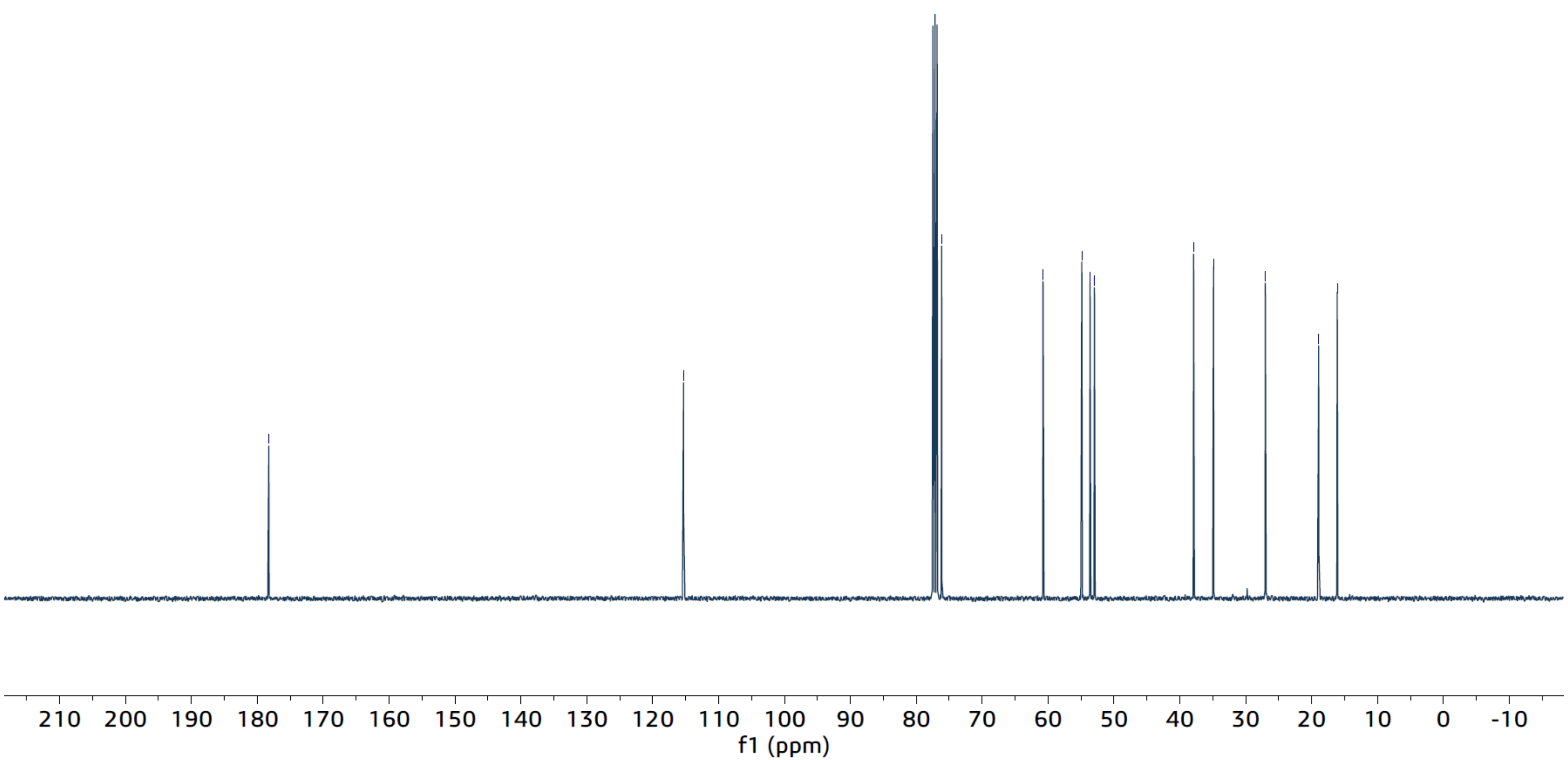

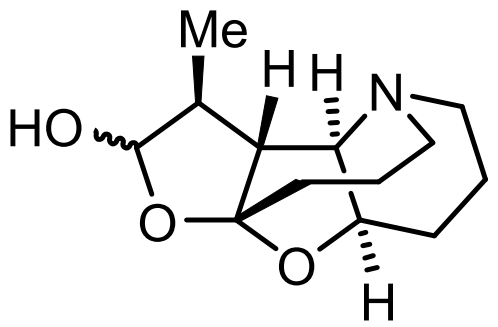

35 (dr 1.7:1)
^{1}H NMR (400 MHz, $CDCl_3$)

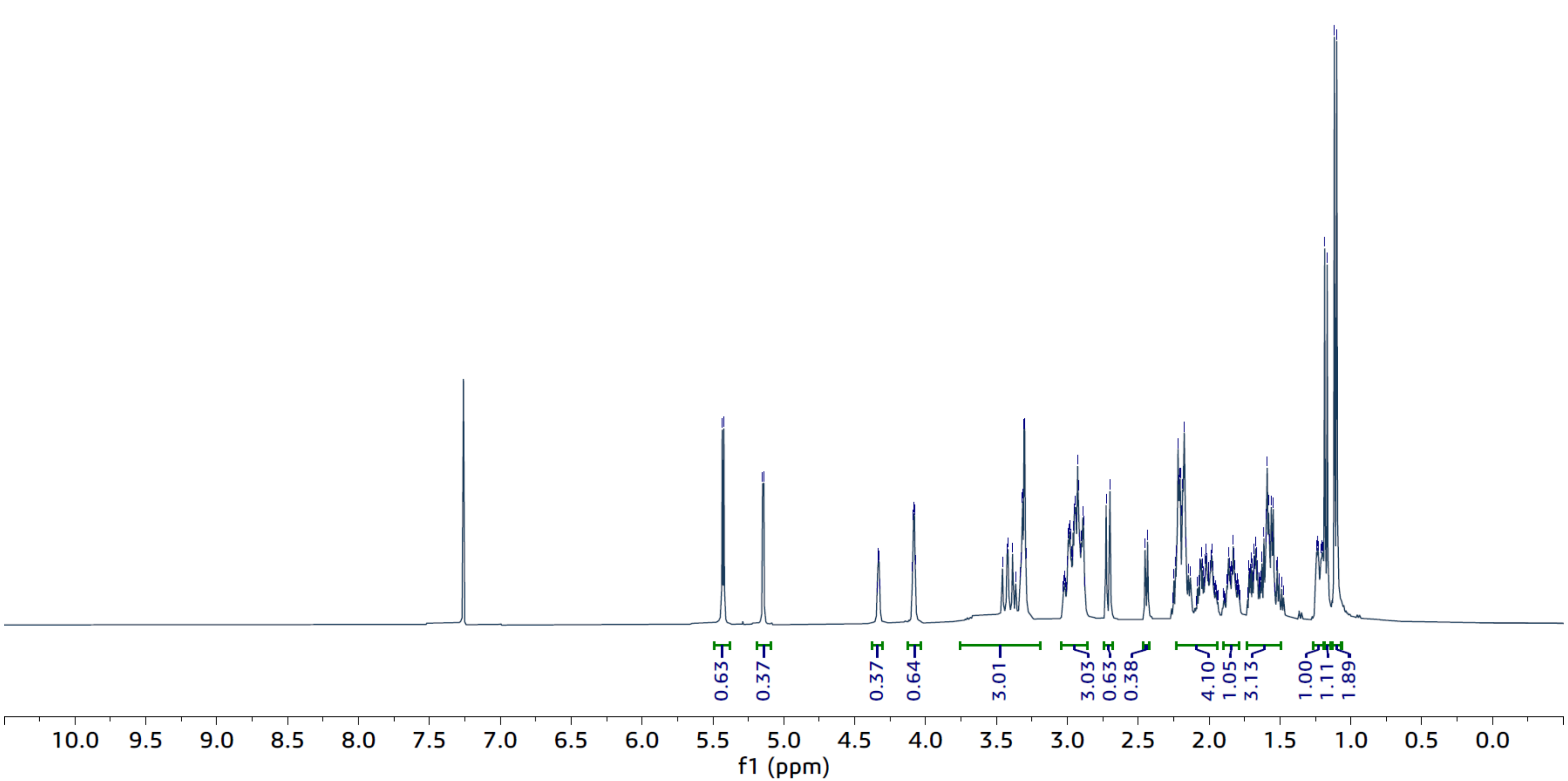

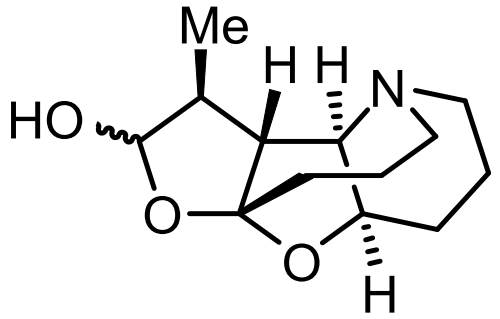

35 (dr 1.7:1)

^{13}C NMR (101 MHz, $CDCl_3$)

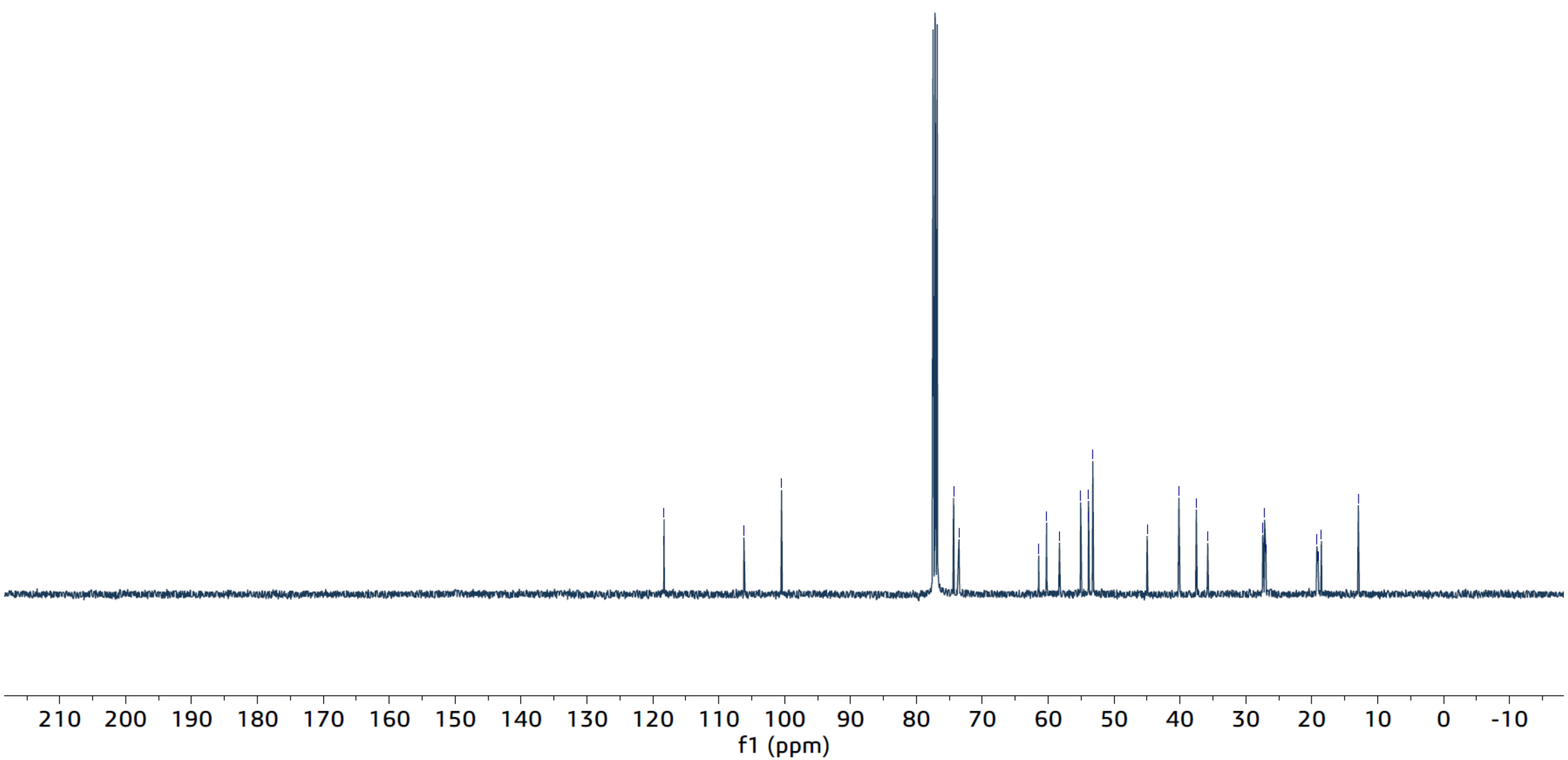

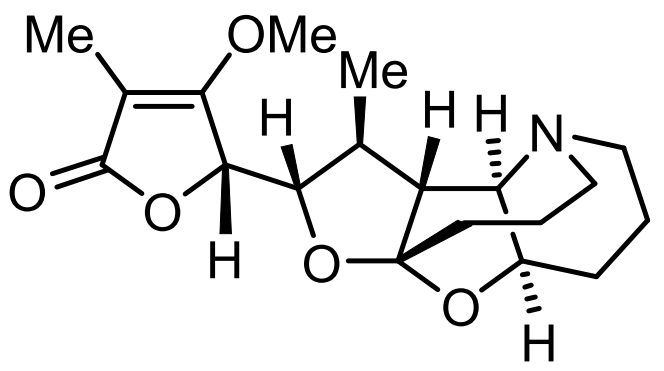

37b

^{1}H NMR (600 MHz, $CDCl_3$)

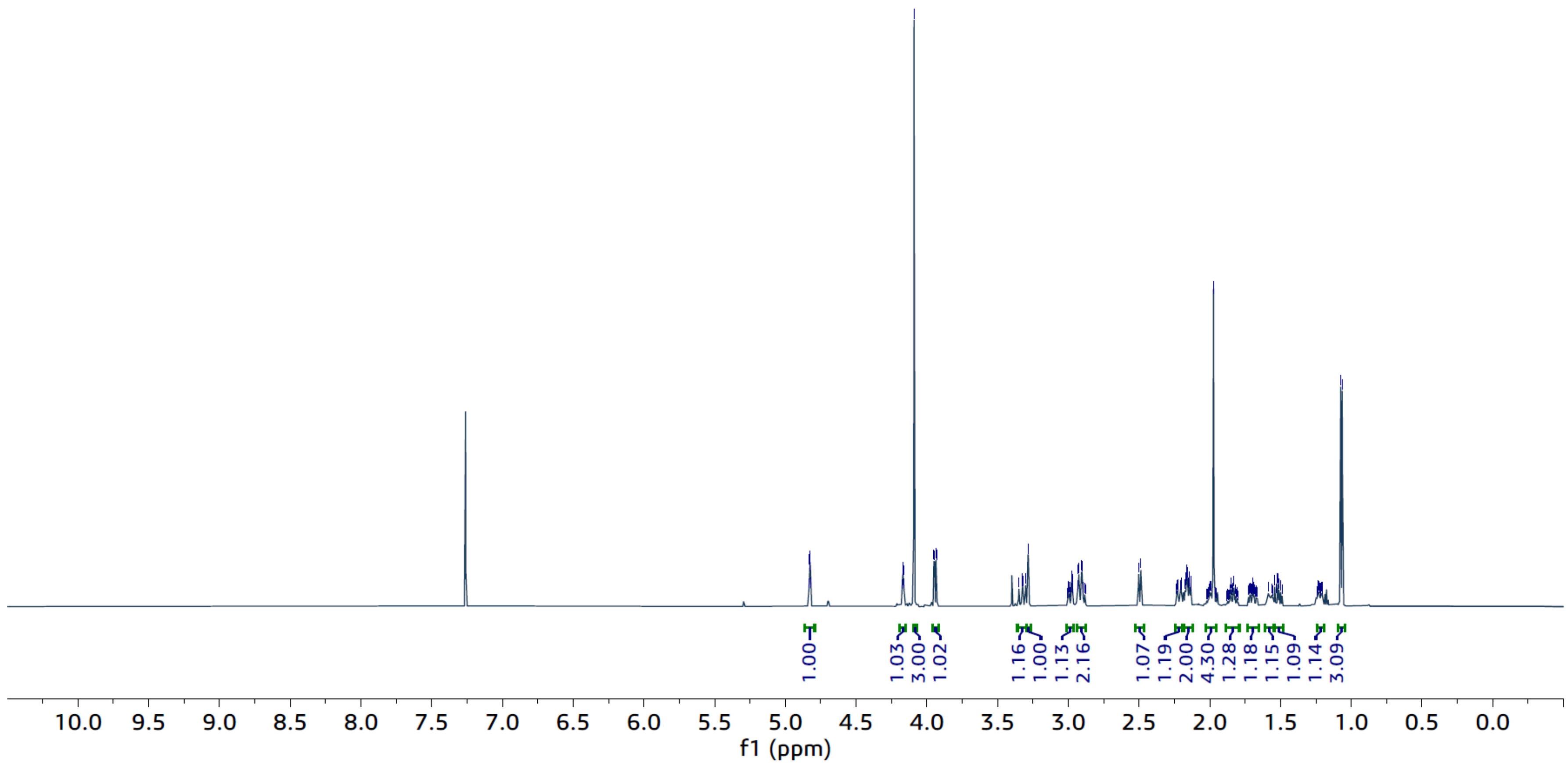

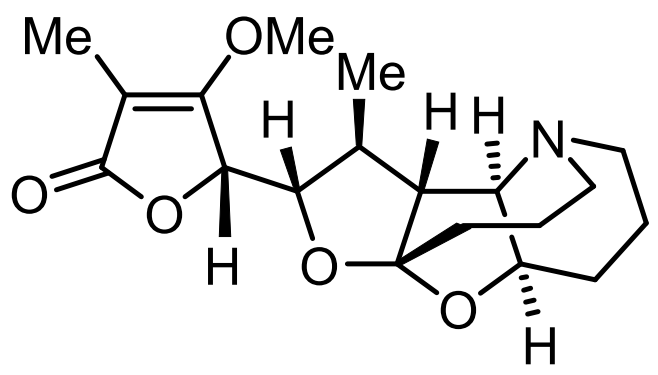

37b

^{13}C NMR (151 MHz, $CDCl_3$)

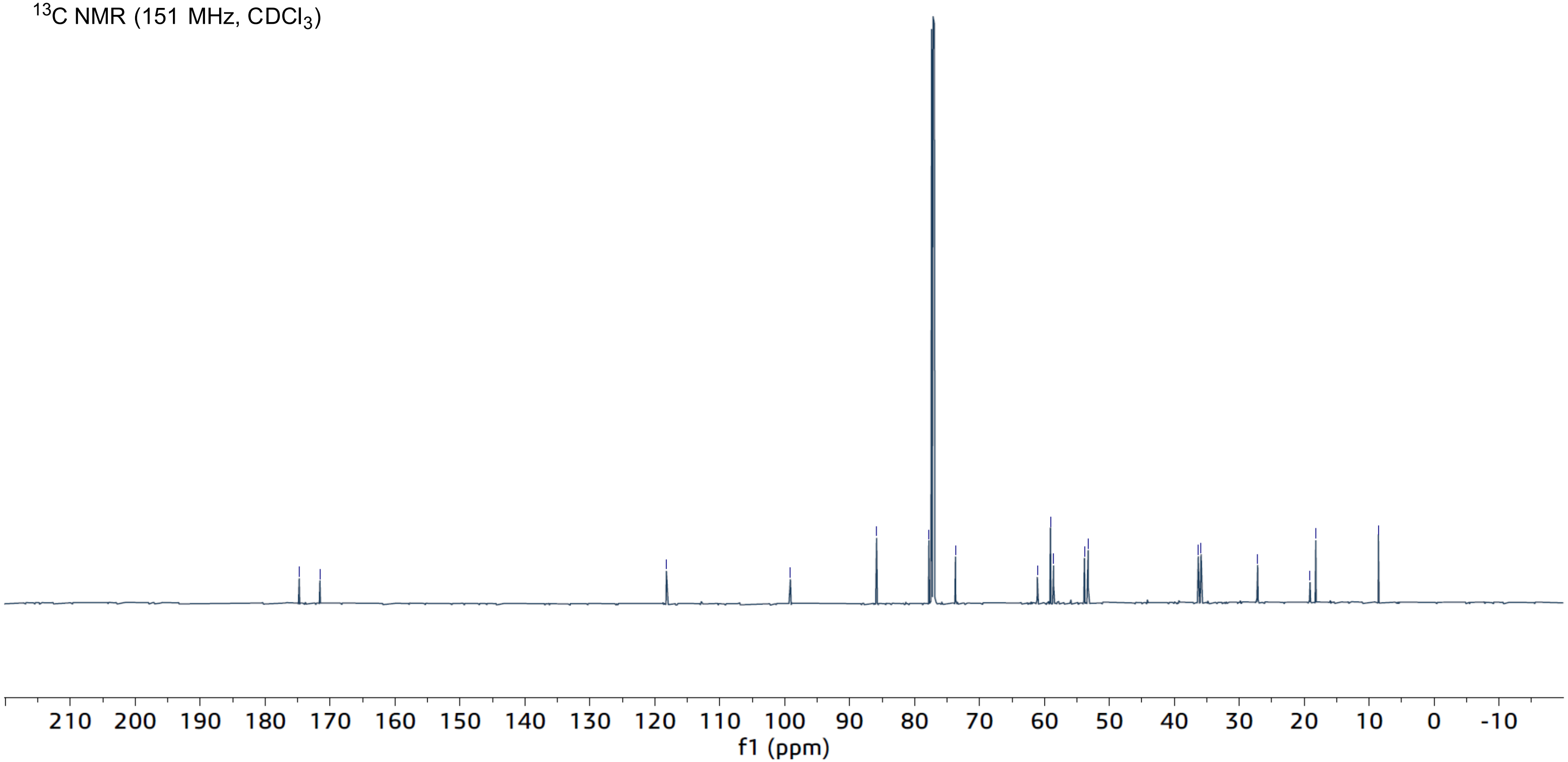

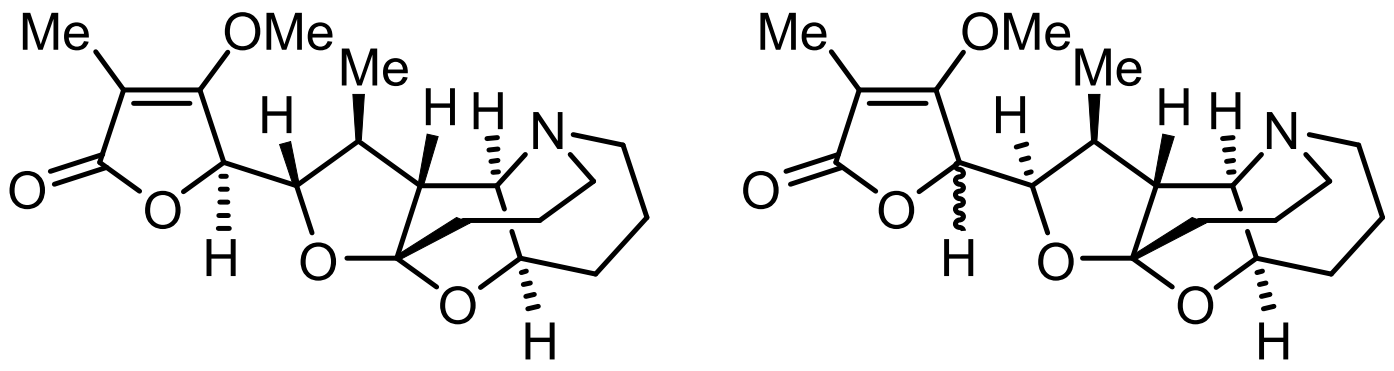

37a / 37c (dr 1.25:1)
^{1}H NMR (600 MHz, $CDCl_3$)

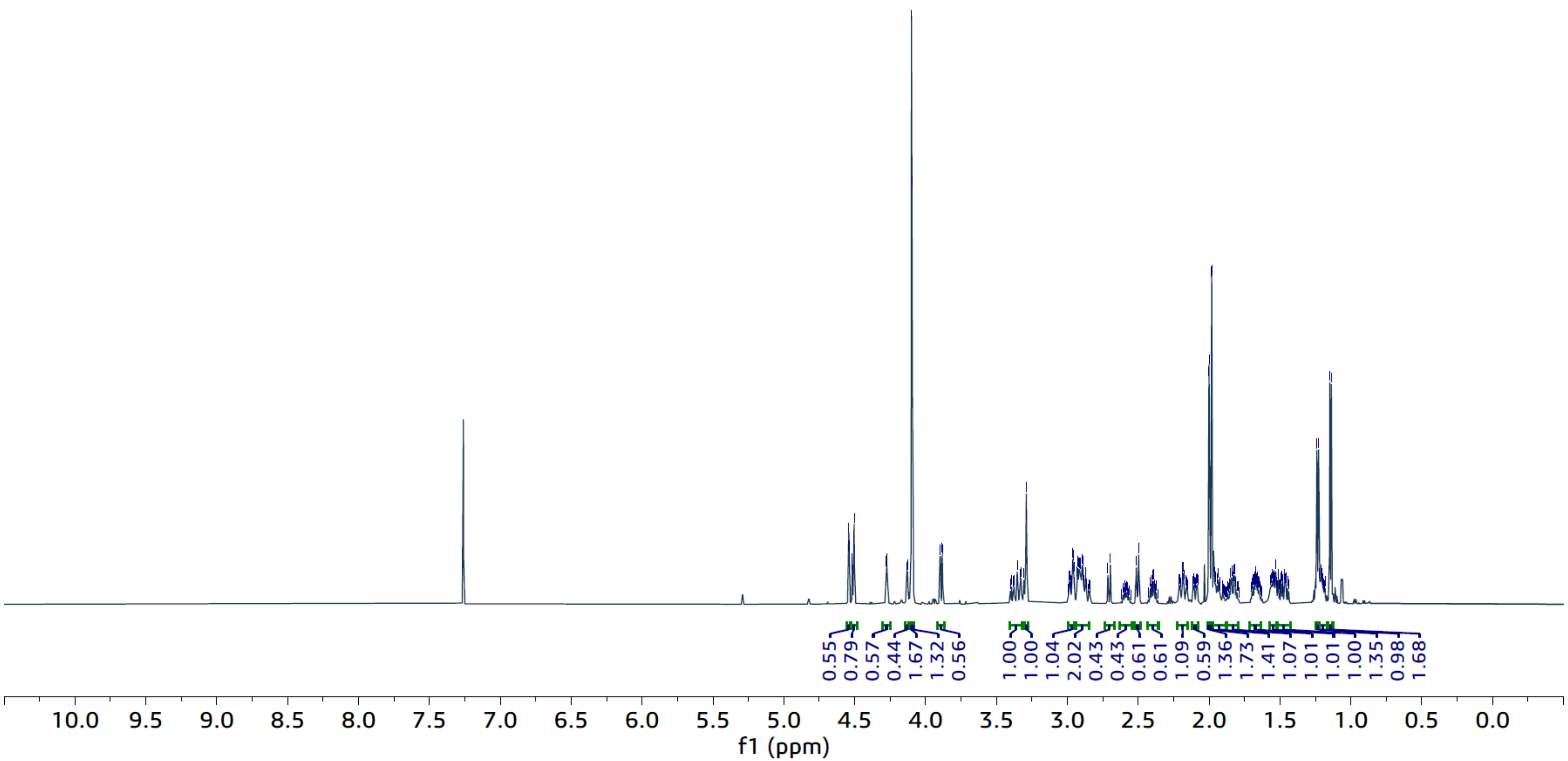

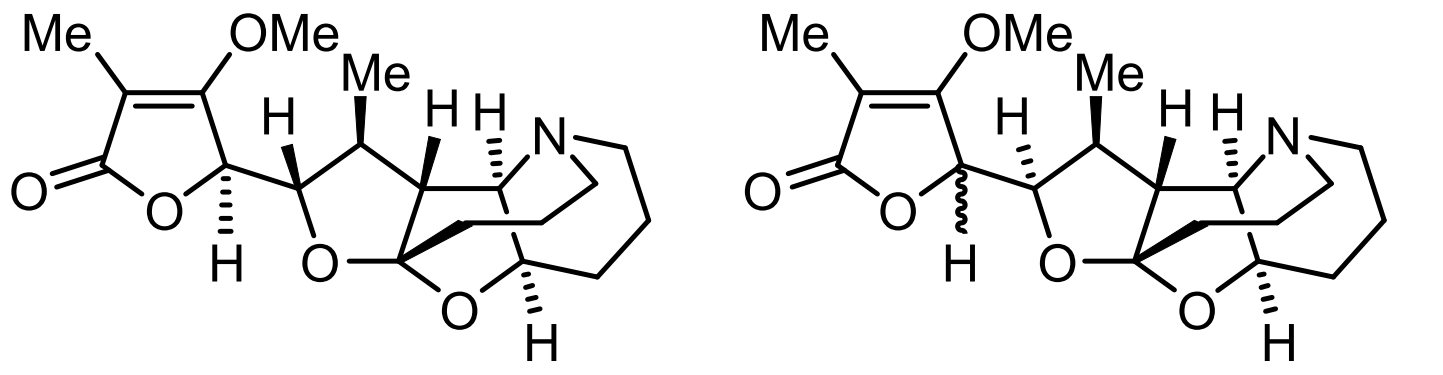

37a / 37c (dr 1.25:1)
^{13}C NMR (151 MHz, $CDCl_3$)

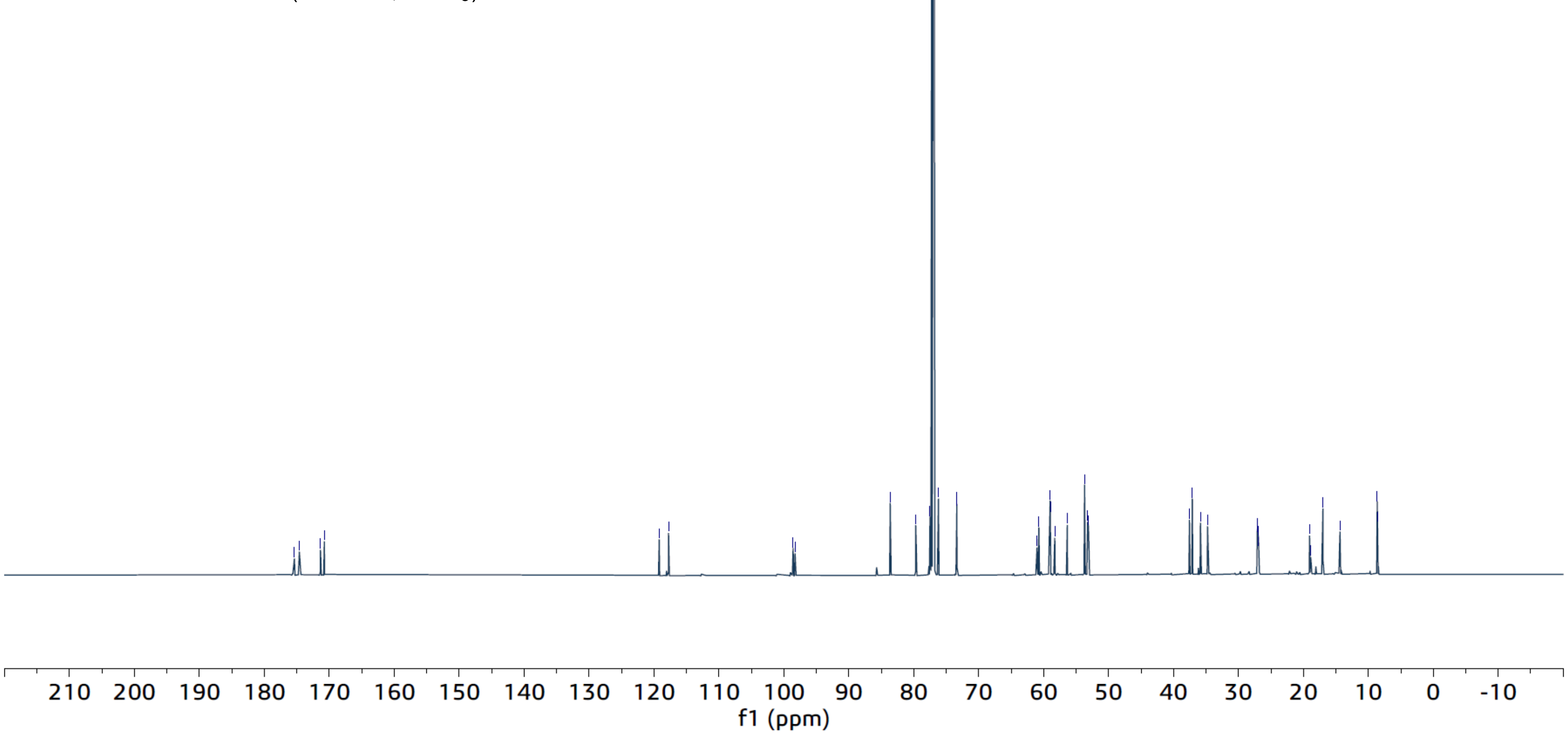

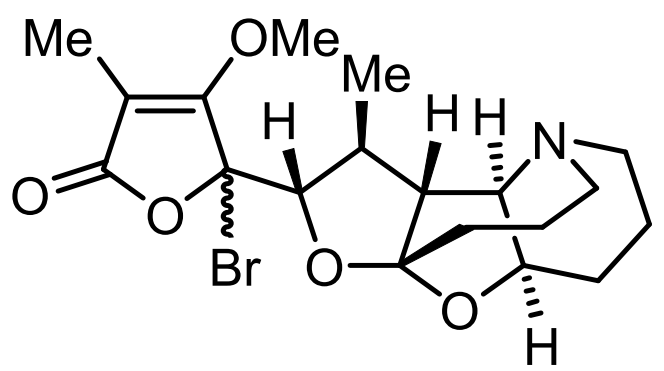

38 (dr 4.5:1)
^{1}H NMR (400 MHz, $CDCl_3$)

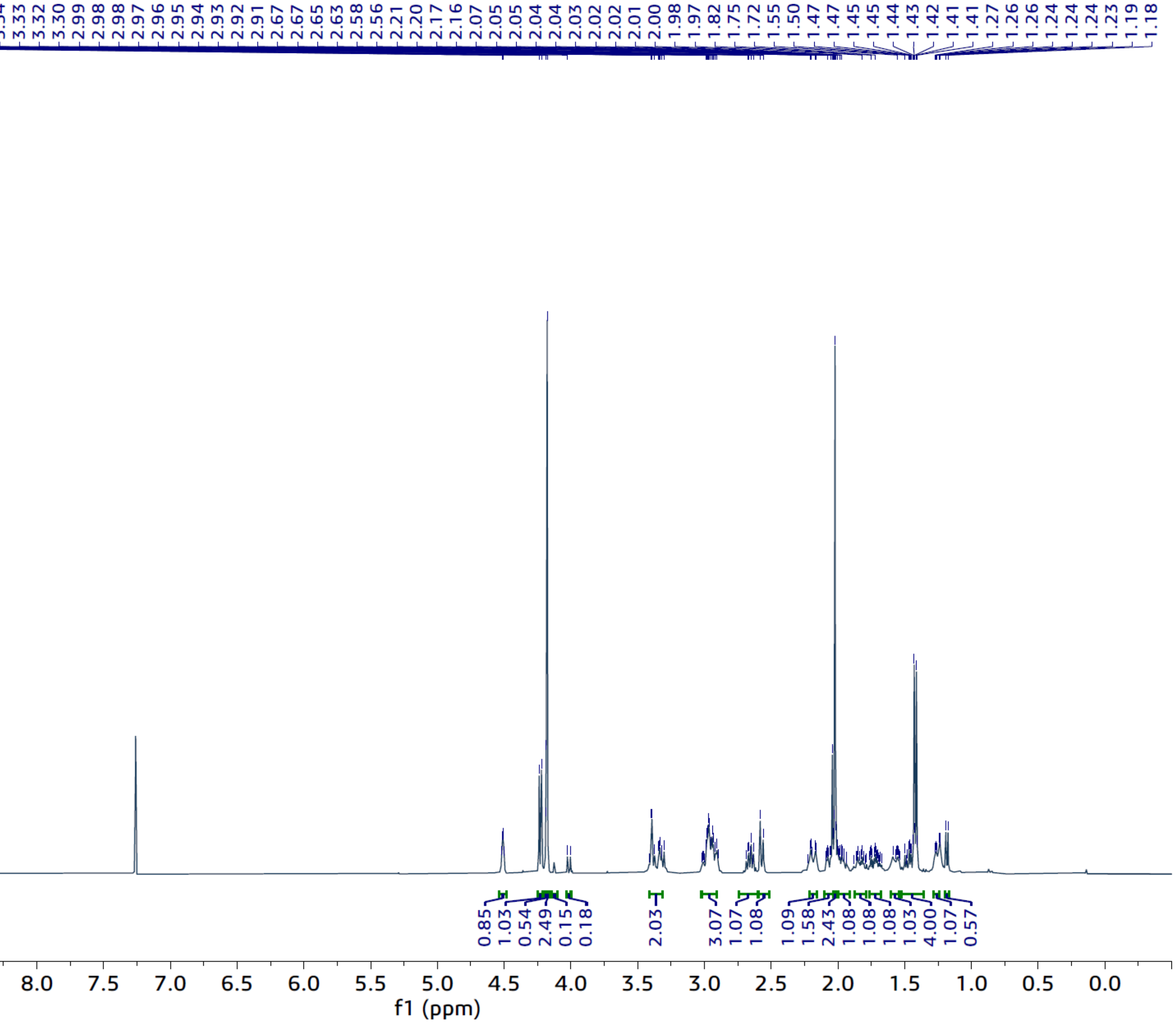

38 (dr 4.5:1)
^{13}C NMR (101 MHz, $CDCl_3$)

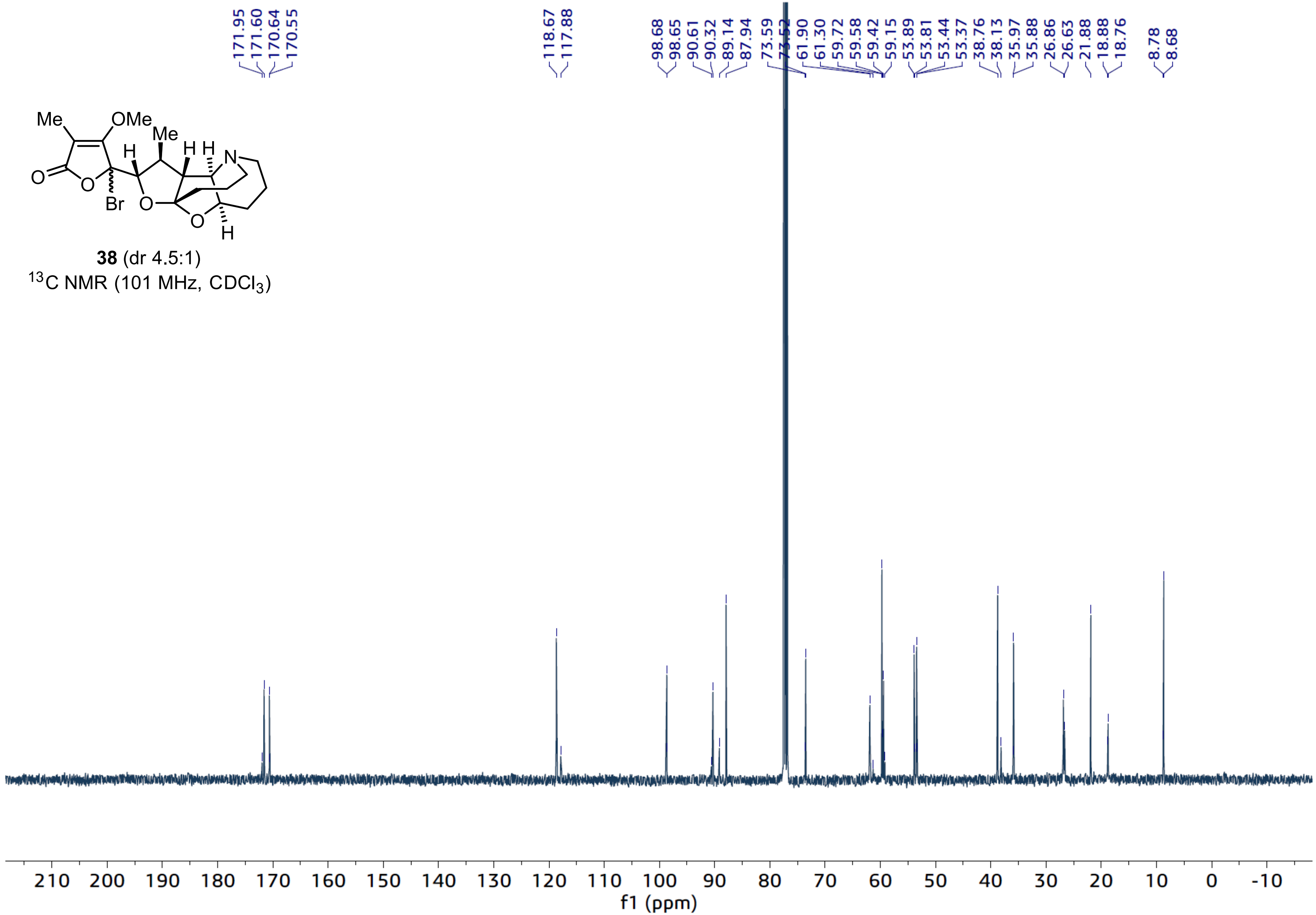

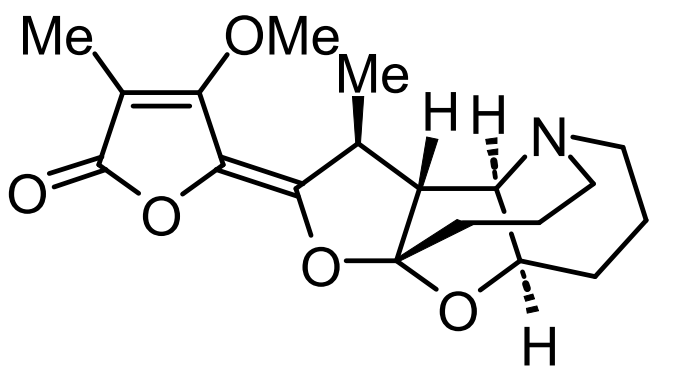

Stemocurtisine (**1**)
^{1}H NMR (600 MHz, $CDCl_3$)

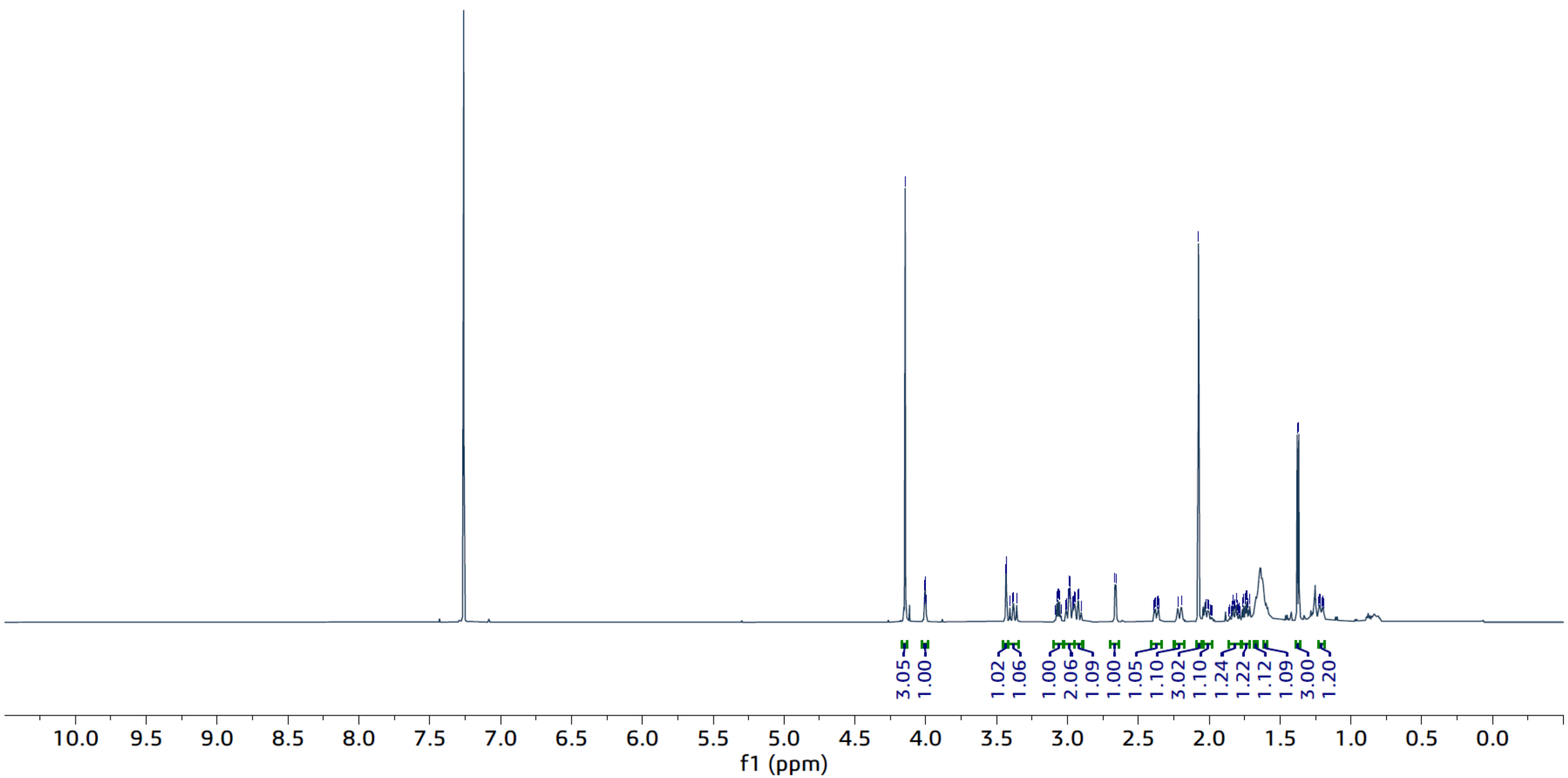

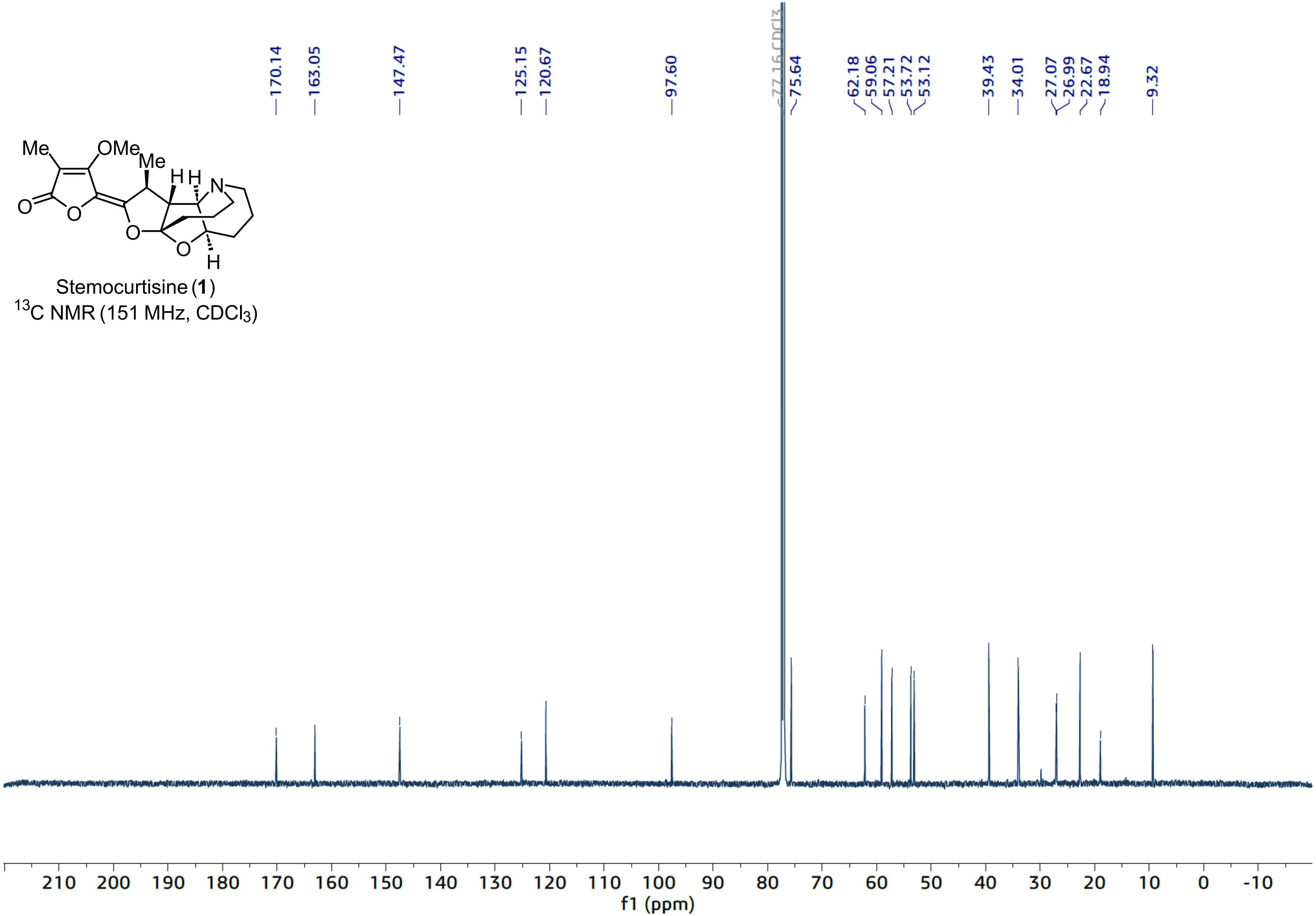

Stemocurtisine (**1**)
^{13}C NMR (151 MHz, $CDCl_3$)

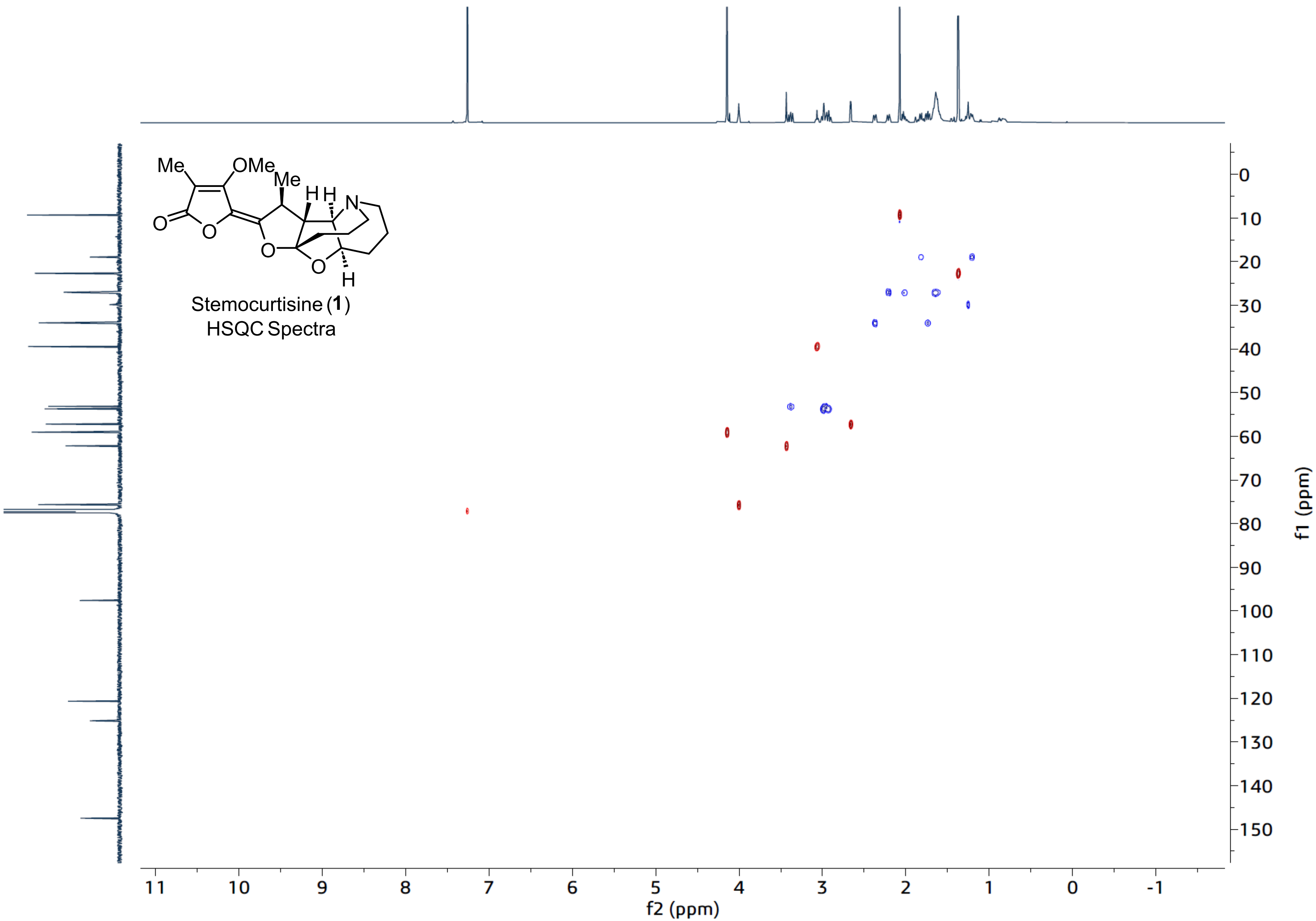

Me
OMe
Me
H H
N
O
O
O
O
H
Stemocurtisine (1)
HSQC Spectra
f1 (ppm)
f2 (ppm)

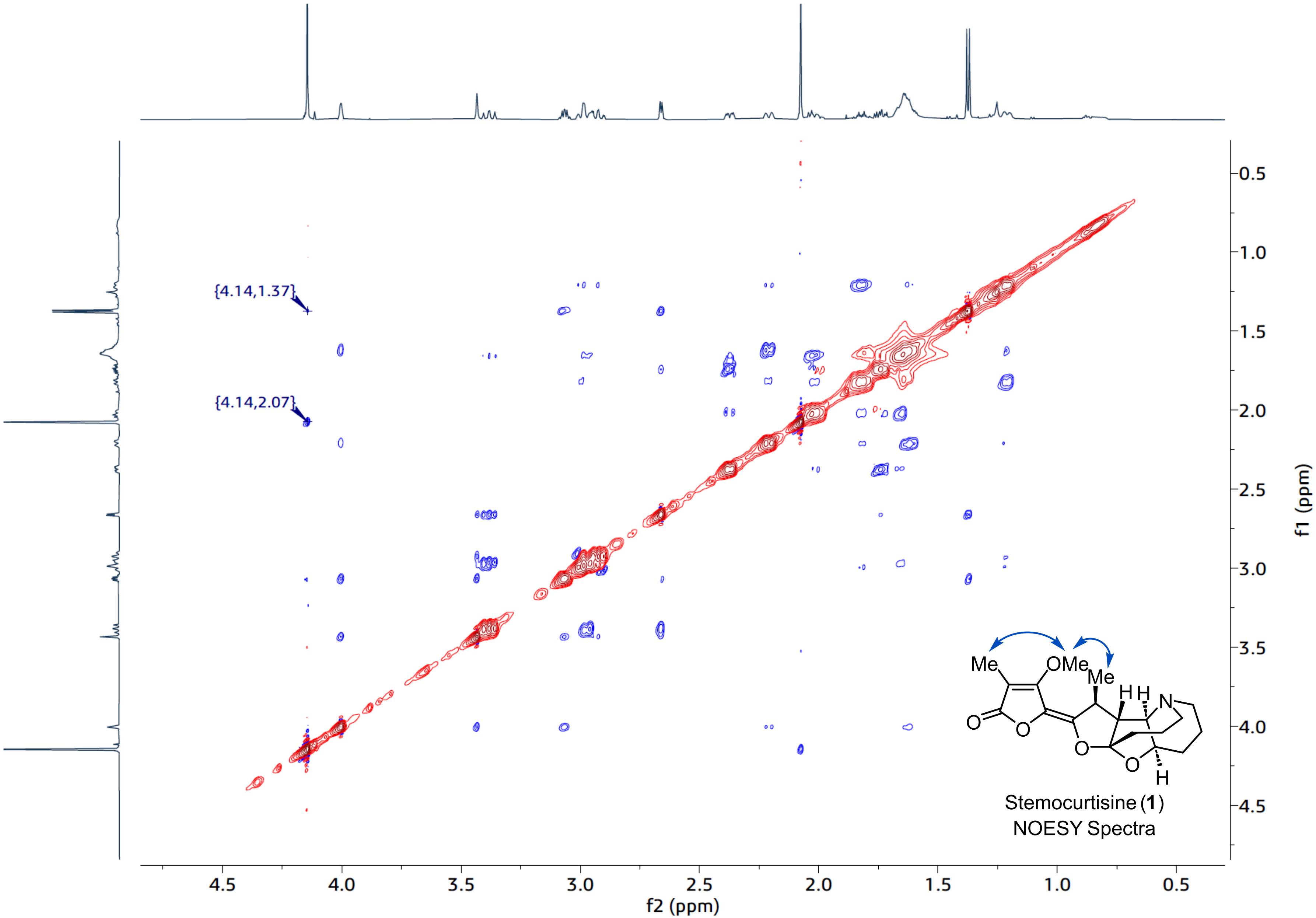
{4.14,1.37}
{4.14,2.07}
Me
OMe
Me
H H
N
O
O
O
O
H
Stemocurtisine (1)
NOESY Spectra
f2 (ppm)
f1 (ppm)
4.5
4.0
3.5
3.0
2.5
2.0
1.5
1.0
0.5
